# Long‐term outcomes of cribriform‐positive and cribriform‐negative prostate cancer treated with radical prostatectomy in the ProtecT trial

**DOI:** 10.1111/bju.70261

**Published:** 2026-03-27

**Authors:** Nikita Sushentsev, Anne Y. Warren, Richard Colling, Clare Verrill, Ekaterina Pazukhina, Oleg Blyuss, Alexey Zaikin, Tyler M. Seibert, Tristan Barrett, Jon Oxley, Ian G. Mills, Richard J. Bryant, J. Athene Lane, Jenny L. Donovan, David E. Neal, Freddie C. Hamdy

**Affiliations:** ^1^ Department of Radiology University of Cambridge Cambridge UK; ^2^ Department of Pathology Cambridge University Hospitals NHS Foundation Trust Cambridge UK; ^3^ Nuffield Department of Surgical Sciences University of Oxford Oxford UK; ^4^ Wolfson Institute of Population Health Queen Mary University of London London UK; ^5^ Department of Women's Cancer, Institute for Women's Health University College London London UK; ^6^ Department of Mathematics University College London London UK; ^7^ Department of Histopathology North Bristol Hospitals Trust Bristol UK; ^8^ Population Health Sciences, Bristol Medical School University of Bristol Bristol UK; ^9^ Department of Radiation Medicine and Applied Sciences University of California San Diego San Diego CA USA; ^10^ Department of Urology University of California San Diego La Jolla CA USA; ^11^ Department of Bioengineering University of California San Diego Jacobs School of Engineering La Jolla CA USA; ^12^ Department of Radiology University of California San Diego School of Medicine La Jolla CA USA; ^13^ Institute for Cognitive Neuroscience National Research University Higher School of Economics Moscow Russia; ^14^ Department of Pediatrics and Pediatric Infectious Diseases Institute of Child's Health, Sechenov University Moscow Russia

**Keywords:** prostate cancer, radical prostatectomy, cribriform adenocarcinoma, intraductal carcinoma, metastasis

## Abstract

**Objectives:**

To retrospectively analyse the results of the Prostate Testing for Cancer and Treatment (ProtecT; ClinicalTrials.gov identifier: NCT02044172) trial to establish the association between cribriform‐positive and ‐negative prostate cancer (PCa) and the 15‐year risk of metastasis or death from PCa in patients who underwent radical prostatectomy (RP).

**Patients and Methods:**

Between 1999 and 2009, the ProtecT phase 3 clinical trial enrolled 1643 men with clinically localised PCa who were randomised to receive active monitoring, RP, or radiotherapy. In this secondary analysis of the trial, a centralised histopathological review was conducted on available RP pathology slides to classify patients as cribriform‐positive if they had invasive cribriform carcinoma and/or intraductal carcinoma. The primary outcome was a composite of progression to metastatic disease or death from PCa. Exposures included age, prostate‐specific antigen density, RP Grade Group (GG), pathological T stage (pT), and cribriform status. Multivariable Cox proportional hazards regression models assessed 15‐year risk. Cumulative incidence curves were compared using the Gray test.

**Results:**

Of 480 men with RP specimens reviewed, 143 (30%) had cribriform‐positive disease and 337 (70%) had cribriform‐negative disease. All 21 metastatic or lethal events occurred exclusively in the cribriform‐positive group (15‐year cumulative incidence 14%). Within the cribriform‐positive cohort, risk was concentrated in patients with pT3b stage and/or GG ≥3 (15‐year cumulative incidence 27%). In multivariable analysis of cribriform‐positive patients, pT3b stage (hazard ratio [HR] 8.19, 95% confidence interval [CI] 2.39–28.10; *P* < 0.001) and GG 3 disease (HR 5.12, 95% CI 1.59–16.40; *P* = 0.006) were independent predictors of adverse outcomes. Conversely, cribriform‐positive patients with GG 2 and ≤pT3a had a 15‐year event rate of only 3%.

**Conclusion:**

In the ProtecT trial, the 15‐year risk of metastasis or death after RP was a binary outcome defined by cribriform status. The concentration of risk in men with cribriform‐positive, high‐grade and/or pT3b tumours identifies a target population for adjuvant therapy trials, while supporting management de‐escalation for most RP patients.

**Abbreviations**
ADT: androgen deprivation therapy
GG: Grade Group
HR: hazard ratio
ICC: invasive cribriform carcinoma
IDC: intraductal carcinoma
ISUP: International Society of Urological Pathology
PCa: prostate cancer
pT: pathological T stage
RP: radical prostatectomy
SVI: seminal vesicle invasion

## Introduction

The global prostate cancer (PCa) incidence and mortality are estimated to double by 2040 [1], underscoring the importance of early detection and appropriate management of potentially incurable and lethal disease. While active surveillance is appropriate for low‐risk and some intermediate‐risk cancers, radical prostatectomy (RP) and radiotherapy continue to offer primary curative options for localised PCa. In the 15‐year analysis of the Prostate Testing for Cancer and Treatment (ProtecT; ClinicalTrials.gov identifier: NCT02044172) phase 3 randomised trial, RP and radiotherapy both halved the risk of metastasis compared to active monitoring; however, this did not translate into reduced PCa‐specific mortality [2].

A recent review of diagnostic biopsies from the trial [3] clarified that the excess risk of metastasis was mostly confined to the 13% of patients harbouring cribriform PCa, a specific morphological phenotype within broader Gleason patterns 4 and 5 [4]. In these patients, primary radiotherapy with neoadjuvant androgen deprivation therapy (ADT) was associated with decreased 15‐year cumulative incidence of metastasis compared to active monitoring. Conversely, while primary RP significantly delayed metastasis onset in cribriform‐positive patients, their outcomes at 15 years were similar to patients assigned to active monitoring. These hypothesis‐generating results suggest that a subset of men with cribriform‐positive disease undergoing primary RP are at increased risk of long‐term metastasis and may therefore benefit from adjuvant treatment to improve their outcomes.

To better characterise this high‐risk group, we conducted a centralised review of all available RP specimens from the ProtecT trial. While our previous work [3] utilised diagnostic biopsy specimens to evaluate cribriform status, the present study leveraged definitive RP pathology to overcome the inherent sampling limitations of biopsies. By evaluating the 15‐year risk of metastasis or death from PCa among patients with cribriform‐positive and ‐negative disease at RP, we aimed to provide a more granular risk‐stratification to inform future trials of targeted adjuvant therapy or management de‐escalation in patients undergoing RP.

## Patients and Methods

### Study Design and Participants

The ProtecT trial is a randomised phase 3 clinical trial designed to compare the major conventional treatments for patients with clinically localised PCa detected through population‐based PSA testing. Recruitment to the main trial was undertaken from October 2001 to January 2009 in nine UK cities. The trial design [5], 10‐year [6], and 15‐year [2] outcomes have been published previously. All participants provided written informed consent. The study, which is still ongoing, is funded by the National Institute for Health and Care Research, sponsored by the University of Oxford, and approved by the East‐Midlands Multicentre Research Ethics Committee (01/4/025). The protocol is available in the Appendix S1.

### Randomisation and Surgical Management

Independent trial steering and safety committees provided oversight. Participants were randomised with minimisation by clinical site, sociodemographic, and clinical factors. Clinical management followed trial‐specific pathways with an annual research review [7]. In patients randomly assigned to receive RP, along with patients who declined randomisation but expressed preference for RP, the predominant approach was open retropubic RP with individual‐level quality assurance according to minimum standards [8]. Participants with a baseline PSA concentration of at least 10 μg/L or a biopsy Gleason score of at least 7 points were offered bilateral lymphadenectomy. Postoperatively, PSA concentrations were measured every 3 months for the first year, every 6 months for 2 years, and then yearly. Adjuvant radiotherapy was discussed and offered to patients with positive surgical margins and/or extracapsular disease. Participants underwent clinical review if their postoperative PSA concentrations reached ≥0.2 μg/L to discuss adjuvant radiotherapy [5].

### Histopathological Review

Available surgical biopsies were retrieved and independently reviewed by two expert genitourinary pathologists (A.Y.W. and R.C.), with discrepancies resolved by a third (C.V.). Pathologists were blinded to patient outcomes. Since the original histological reporting was conducted using the 2005 International Society of Urological Pathology (ISUP) recommendations, the contemporary 2019 ISUP recommendations were used for revised Gleason score and corresponding Grade Group (GG) assignment [9]. The most recent ISUP consensus definition [4] was used to classify patients as ‘cribriform‐positive’ if they had invasive cribriform carcinoma (ICC) and/or intraductal carcinoma (IDC). As high‐molecular‐weight‐keratin immunohistochemistry was not routinely available, IDC was diagnosed based on histopathological examination, defined as prostatic ducts filled up and distended by cribriform or solid carcinoma [10]. In addition to binary cribriform status, we assessed the percentage of cribriform disease, which included both cribriform Gleason pattern 4 (ICC/IDC) and Gleason pattern 5 glands comprised of cribriform structures with comedonecrosis [9]. Moreover, we distinguished small (≤0.25 mm in diameter) and large (>0.25 mm in diameter) cribriform size patterns according to a previously proposed quantitative definition [11].

### Outcomes and Exposures

The primary outcome was a composite of progression to metastatic disease (bony, visceral, or lymph node metastases on imaging, or PSA concentration >100 μg/L) and/or death from PCa, as adjudicated by an independent cause‐of‐death committee [12]. Exposures included age, PSA density (PSA divided by the RP sample weight), final GG, pathological T stage (pT), surgical margin status, cribriform status, percentage of cribriform disease, and size of cribriform structures from centralised surgical pathology review.

### Statistical Methods

Multivariable Cox proportional‐hazards models assessed 15‐year risk of metastasis or death from PCa to evaluate aetiological associations between clinicopathological variables and the primary outcome and thereby guide future research. Cumulative incidence curves were compared using the Gray's test to account for the presence of competing risks. A two‐sided *P* < 0.05 was considered statistically significant.

Statistical analysis was conducted in two main patient cohorts. The primary analysis was performed on the per‐protocol cohort, restricted to patients who were randomly assigned to RP and subsequently underwent the procedure as their initial treatment. The rationale for this subgroup analysis was two‐fold: first, to assess the efficacy of RP as a primary, upfront curative treatment in a cohort free from the potential confounders of delayed intervention or lack of randomisation; and second, to provide a cohort for calibrating diagnostic biopsy findings against definitive RP pathology, with a possibility to extrapolate these results to the other randomised groups.

A secondary, full treatment‐received cohort analysis included all patients who underwent RP at any point in the trial, irrespective of their initial treatment allocation. This approach was chosen to evaluate the long‐term effectiveness of RP and the prognostic value of individual histopathological features in a pragmatic population with divergent clinical pathways to RP, which is more representative of daily clinical practice.

## Results

**Fig. 1:**
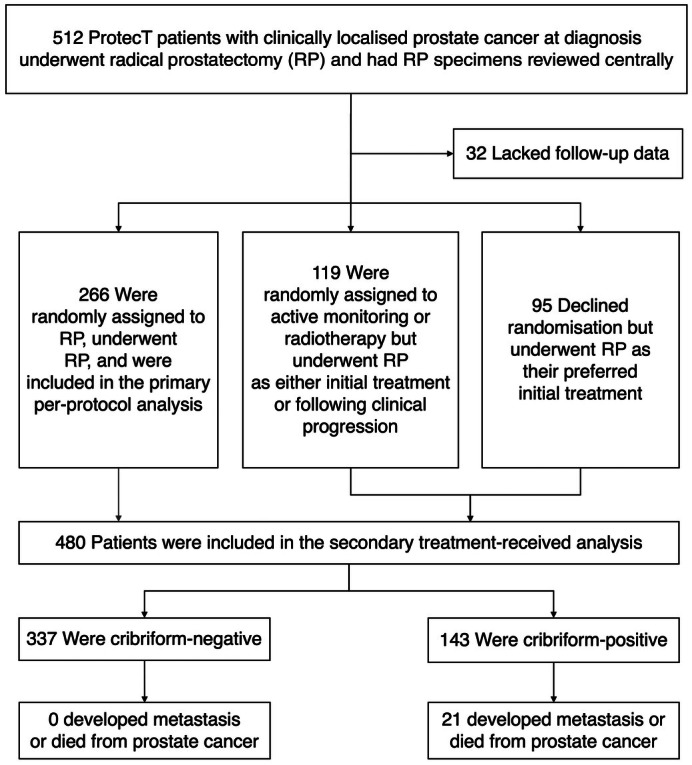
Study flowchart.

Of 761 trial participants who underwent RP as either allocated or preferred treatment, 512 (67%) had RP specimens available for centralised histopathological review, and 480 (63%) had sufficient follow‐up data to be included in the analysis (Fig. 1). Of the 480 patients, who comprised the full treatment‐received cohort, 266 were randomly assigned to and treated with upfront RP, comprising the per‐protocol cohort (Table 1). Further histological characteristics of patients from both cohorts are provided in Appendix S2. Importantly, while all nine trial sites were represented in this analysis, RP specimen availability varied by centre due to historical archival logistics (Table S1). Despite this variability, randomisation characteristics were comparable between patients with available and unavailable specimens (Table S2).

**Table 1:** Clinical characteristics of the per‐protocol cohort.

Characteristic	Per‐protocol cohort: all patients (*n* = 266)	Per‐protocol cohort: patients with metastatic or lethal disease (*n* = 7)
Age, years, mean (SD)	62 (5)	63 (5)
PSA concentration, μg/L, mean (SD)	6.7 (3.7)	5.8 (3)
Tumour GG at RP, *n* (%)		
GG 1	127 (48)	0
GG 2	109 (41)	0
GG 3	24 (9)	5 (86)
GG 4	3 (1)	1 (7)
GG 5	3 (1)	1 (7)
Tumour pT stage at RP, *n* (%)		
pT2	198 (74)	1 (14)
pT3a	60 (23)	3 (43)
pT3b	7 (2)	3 (43)
pT4	1 (1)	0
Tumour cribriform status at RP, *n* (%)		
Cribriform‐negative	198 (84)	0
Cribriform‐positive	68 (26)	7 (100)

**Fig. 2:**
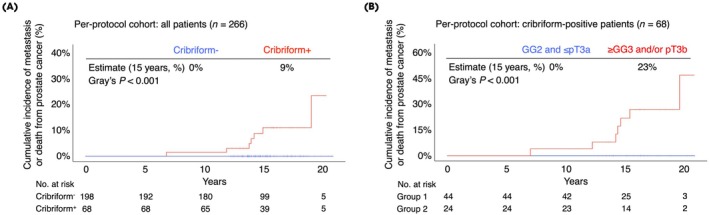
Primary outcome cumulative incidence analysis in the per‐protocol cohort. **(A)** Data are from a 15‐year cumulative analysis of the full per‐protocol cohort. **(B)** Data are from a 15‐year cumulative incidence analysis of two subgroups of cribriform‐positive patients from the per‐protocol cohort. Numbers at risk for Group 1 correspond to cribriform‐positive patients with GG 2 and ≤pT3a disease; similar numbers for Group 2 correspond to cribriform‐positive patients with GG ≥3 and/or pT3b disease. Two‐sided *P* values were calculated using Gray's test.

In the per‐protocol cohort, all seven instances of metastasis or death from PCa occurred exclusively in the 68/266 (26%) patients with cribriform‐positive disease at RP, corresponding to a 15‐year cumulative incidence of 9% (Fig. 2A). The binary risk was further concentrated within a specific subgroup of 24 cribriform‐positive patients who also had GG ≥3 disease and/or seminal vesicle invasion (SVI; pT3b stage). This subgroup, representing 9% of the cohort, experienced all seven events and therefore had a 23% cumulative incidence of metastatic or lethal disease at 15 years (Fig. 2B). Conversely, no events occurred in the remaining 198/266 (74%) cribriform‐negative patients.

In 123/266 (46%) patients from the per‐protocol cohort who had both RP and biopsy samples available for review, the sensitivity of systematic biopsy for predicting binary cribriform status against RP pathology was 39.5% (95% CI 25.0–55.6%; Table S3). However, for predicting the presence of cribriform‐positive, GG ≥3 and/or pT3b disease at RP, the sensitivity of targeted biopsy was higher at 52.9% (95% CI 27.8–77.0%; Table S4).

**Fig. 3:**
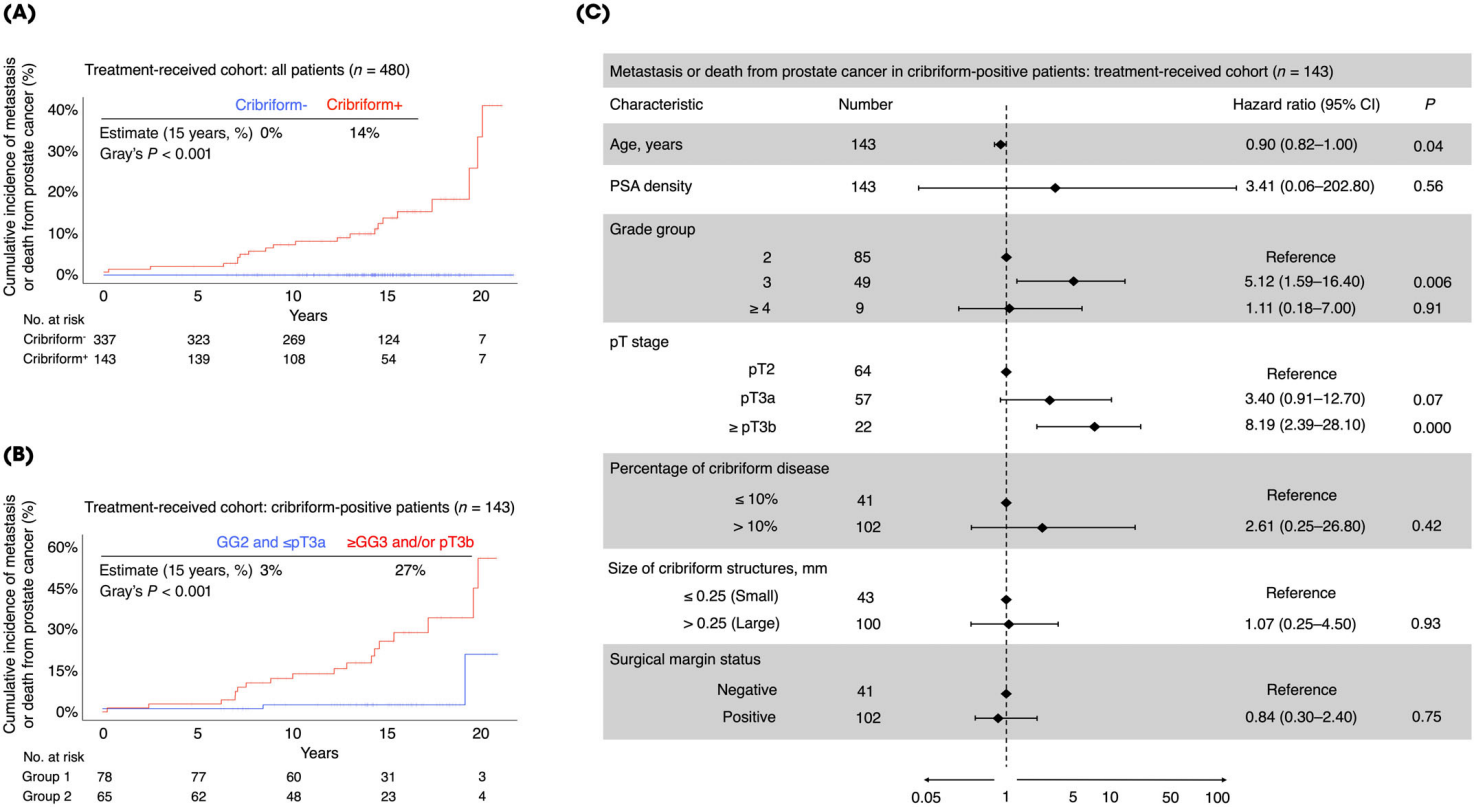
Primary outcome analysis in the treatment‐received cohort. **(A, B)** Data are from the treatment‐received cohort. Numbers at risk for Group 1 correspond to cribriform‐positive patients with GG 2 and ≤pT3a disease; similar numbers for Group 2 correspond to cribriform‐positive patients with GG ≥3 and/or pT3b disease. Two‐sided *P* values were calculated using Gray's test. **(C)** Multivariable Cox proportional‐hazard model plot for treatment‐received cohort, with diamonds indicating HRs and horizontal lines indicating 95% CIs, with corresponding two‐sided *P* values provided as appropriate.

To confirm the robustness of our main findings, we repeated the analysis in the full treatment‐received cohort (*n* = 480) that also included trial participants who underwent RP for reasons such as personal preference or clinical progression on active monitoring. Clinical characteristics of this cohort are presented in Table 2. As in the per‐protocol cohort, all 21 adverse events occurred exclusively among the 143/480 (30%) patients with cribriform‐positive disease at RP, yielding a 15‐year cumulative incidence of 14% (Fig. 3A). Again, the risk was concentrated in 65 cribriform‐positive patients with GG ≥3 and/or pT3b disease (14% of the cohort), whose 15‐year cumulative incidence of adverse events was 27% (Fig. 3B). Only two adverse events occurred in cribriform‐positive patients with GG 2 and ≤pT3a disease, yielding a 15‐year cumulative incidence of 3%. Multivariable analysis confirmed that pT3b stage (hazard ratio [HR] 8.19, 95% CI 2.39–28.10) and GG 3 disease (HR 5.12, 95% CI 1.59–16.40) were the only two independent predictors of adverse outcomes in cribriform‐positive patients in the treatment‐received cohort (*P* < 0.005 for both; Fig. 3C). Notably, even among cases with any Gleason pattern 5 component, metastatic or lethal disease was only seen in patients whose Gleason pattern 5 was of cribriform morphology (Fig. S1). Allowing for the lack of consistent availability basal cell immunohistochemistry, the distribution of patients with morphologically diagnosed ICC, IDC, and both patterns in the treatment‐received cohort is also provided in Table S5 for descriptive and hypothesis‐generating purposes.

**Table 2:** Clinical characteristics of the treatment‐received cohort.

	Treatment‐received cohort: all patients (*n* = 480)	Treatment‐received cohort: patients with metastatic or lethal disease (*n* = 21)
Age, years, mean (SD)	63 (5)	63 (5)
PSA concentration, μg/L, mean (SD)	6.3 (3.2)	5.9 (4)
Tumour GG at RP, *n* (%)		
GG 1	218 (45)	0
GG 2	193 (40)	5 (25)
GG 3	63 (13)	14 (67)
GG 4	6 (1)	1 (4)
GG 5	7 (1)	1 (4)
Tumour pT stage at RP, *n* (%)		
pT2	326 (68)	4 (19)
pT3a	129 (27)	8 (38)
pT3b	23 (5)	9 (43)
pT4	2 (<1)	0
Tumour cribriform status at RP, *n* (%)		
Cribriform‐negative	337 (70)	0
Cribriform‐positive	143 (30)	21 (100)

## Discussion

This secondary retrospective analysis of the ProtecT trial suggests that the 15‐year risk of metastasis or death following RP is exclusively confined to men with cribriform‐positive disease in their RP specimens. We also show that the risk is further concentrated among cribriform‐positive patients with GG ≥3 and/or pT3b disease, making them a specific target cohort for the investigation of post‐RP adjuvant therapy. Conversely, patients with cribriform‐negative disease at RP, who constituted 70% of the study cohort, had no metastatic or lethal events at 15 years.

These findings provide long‐term randomised evidence for the diagnostic value of the 2019 ISUP recommendations to report the presence and significance of cribriform morphology (both ICC and IDC) in RP specimens [9]. This analysis also underscores the comparative benefit of routinely reported tumour GG and pT stage over other exploratory metrics (percentage of cribriform disease, size of cribriform structures) for further risk stratification of cribriform‐positive patients. In general, cribriform histology was most prevalent among patients with high‐grade (GG ≥3) and pT3b disease, indicating an association between cribriform status and the overall burden of disease. Interestingly, nearly a third of pT3b tumours had direct SVI by cribriform cells, which may have increased the risk of residual cribriform disease in case of incomplete surgical excision. Conversely, 95% or more of pT3a cases had capsular invasion only by Gleason pattern 3 cells, a low‐risk phenotype not associated with metastatic potential. In addition, while 88% of pT3b cases in this study also had cribriform‐positive cancer, the same was true for only half of pT3a cases. Together, these findings highlight the need to prioritise improved SVI detection on MRI over pT3a prediction, which often either proves challenging in cases of limited extension or increases the risk of overtreatment if overcalled [13].

Another consideration for contemporary clinical practice is the known poor sensitivity of biopsy for determining cribriform status at diagnosis [14]. Although we show the low sensitivity of systematic biopsies for determining binary cribriform status in ProtecT, their sensitivity for detecting cribriform histology in patients with GG ≥3 and/or pT3b tumours was substantially higher, explaining significantly worse outcomes in men with biopsy‐positive cribriform disease observed previously [3]. As contemporary multiparametric MRI‐targeted biopsies on the whole detect more cribriform tumours compared to the systematic approach used in ProtecT [10], it is likely that the current MRI‐driven pathway also misses fewer cribriform cases of this high‐risk group. While this warrants a dedicated investigation in a contemporary cohort, the precision of image‐guided biopsies may be further improved using quantitative or molecular imaging techniques developed specifically to detect cribriform tumours [15, 16]. Encouragingly, a recent multicentre study found that all cribriform‐positive patients enrolled on contemporary active surveillance had MRI‐visible disease at baseline [17].

Taken together, our findings demonstrate that the entire burden of metastatic or lethal disease in the ProtecT RP cohort was confined to patients harbouring cribriform histology in their RP specimens. Although our analysis cannot establish causation, recent phylogenetic work showing a direct clonal link between primary cribriform foci and metastases provides a compelling biological basis for our findings [18]. These results, together with other studies of cribriform disease indicating its distinctly aggressive biological features [19, 20], position this morphology not just as an associated predictive marker, but as a possible driver of some cases of incurable and lethal PCa.

Finally, further identification of a high‐risk RP subgroup of patients with cribriform‐positive, high‐grade and/or pT3b disease establishes a state of clinical equipoise. While the rationale for using adjuvant ADT with or without radiotherapy in these patients may be attractive [21], its toxicity precludes widespread adoption without appropriate evidence, especially when early salvage treatment is a viable alternative. This specific cohort, therefore, represents a target population for future randomised trials determining the optimal post‐RP treatment strategy for high‐risk patients. Equally important, our analysis also shows that for most RP patients, including all cribriform‐negative and most cribriform‐positive patients with low‐grade, localised (pT2) and locally advanced (pT3a) disease, adjuvant therapy is likely unnecessary. In addition, while biochemical recurrence was not a designated endpoint in this trial, the cumulative incidence curves demonstrate a notably delayed onset of metastasis, predominantly occurring several years after RP. Because participants with rising postoperative PSA concentrations (≥0.2 μg/L) were routinely evaluated for standard salvage radiotherapy, this protracted trajectory suggests that isolated, untreated local failure is unlikely to be the sole driver of these late events. Rather, this high‐risk pathological phenotype likely represents a proxy for occult, micrometastatic systemic disease present at the time of RP. It is important to note that contemporary staging modalities, such as prostate‐specific membrane antigen positron emission tomography (PSMA PET), were not available during the ProtecT trial. In current clinical practice, the high rate of systemic progression in this specific cohort provides a strong rationale for utilising early postoperative molecular imaging to either detect metastatic spread earlier or distinguish local from distant failure at the time of recurrence.

This study has several limitations. It is a retrospective, secondary analysis of an ongoing clinical trial with a reported median follow‐up of 15 years. This study is therefore subject to the inherent constraints of the original trial design, including the lack of cribriform status as a randomisation variable. Furthermore, specific details such as nerve‐sparing status were not assessed formally in the analysis, precluding our ability to evaluate their potential influence on surgical margins or long‐term oncological outcomes. Moreover, our re‐estimation of biopsy sensitivity was based on a subset of the per‐protocol cohort for whom diagnostic biopsies were available for review; this may have introduced a selection bias, so the reported estimates should be interpreted with caution despite being consistent with literature [10, 14]. Furthermore, the low number of outcome events in the per‐protocol cohort precluded a separate multivariable analysis due to likely model overfitting, restricting this exploratory analysis to the larger treatment‐received cohort. Notably, as the original trial centralisation protocol yielded a higher volume of RP slides compared to diagnostic biopsy material, the size of the per‐protocol cohort in this study is higher than in the prior biopsy review, necessitating caution when directly comparing their results [3]. A further limitation is the incomplete availability of RP specimens (67%), which was due to historical variations in material centralisation across the nine trial sites. While this reflects logistical rather than clinical selection bias, it may impact the generalisability of the findings across the entire trial population. To address this, arrangements have been put in place to conduct a comprehensive centralisation of all remaining materials for the forthcoming 20‐year trial follow‐up, which will ultimately provide a full‐cohort validation of these results. Finally, while emerging evidence points to potential biological differences between ICC and IDC, we deliberately grouped these entities into a single ‘cribriform‐positive’ category. This binary approach, which we also undertook in the ProtecT biopsy review [22], reflects diagnostic practicality and aligns with the 2019 ISUP guidelines, which prioritise reporting the overall presence of cribriform architecture. While we provide the distribution of ICC and IDC across the study cohort for descriptive purposes, we acknowledge that as basal cell immunohistochemistry was not available in all cases, attempting such granular distinction solely on morphology is inherently prone to misclassification. Therefore, while these results are shown solely for hypothesis‐generating purposes, binary assessment of any cribriform morphology currently remains the most reliable and actionable strategy for clinical risk stratification.

## Conclusion

In conclusion, the presence of cribriform histology in the RP specimens of ProtecT trial participants identified a distinct high‐risk group associated with all metastatic and lethal events observed at 15 years. The observed low sensitivity of systematic biopsies for detecting this pattern means that the true prevalence of aggressive PCa was most likely underestimated across all arms of the ProtecT trial. Moreover, these hypothesis‐generating findings warrant the investigation of adjuvant and early salvage treatment strategies in men with RP treated, cribriform‐positive GG ≥3 and/or pT3b disease.

## Disclosure of Interests

Nikita Sushentsev reported receiving personal fees from Lucida Medical Ltd outside the submitted work. Richard Colling reported receiving grants from MDXHealth Commercial, UK Research and Innovation, and NHSX (for the ARTICULATE PRO study) outside the submitted work; serving as coauthor of the 2024 RCPath (UK) prostate cancer pathology reporting guidelines; receiving partial salary from the NHS; receiving a stipend from Clarendon; and working previously with PathLAKE and PathLAKE+. Clare Verrill reported receiving grants from the National Institute for Health and Care Research (NIHR) Oxford Health Biomedical Research Centre during the conduct of the study and serving as principal investigator of the ARTICULATE PRO study and working previously with PathLAKE and PathLAKE+ outside the submitted work. Tyler M. Seibert reported receiving grants from GE Healthcare and Blue Earth Diagnostics; receiving personal fees from Varian Medical Systems, MJH Life Sciences, GE Healthcare, Blue Earth Diagnostics, and Cortechs.ai; receiving nonfinancial support from Quibim; and receiving stock options for advisory board participation from Cortechs.ai outside the submitted work. Richard J. Bryant reported receiving grants from Cancer Research UK, The Urology Foundation, and the NIHR Health Technology Assessment outside the submitted work. Jenny L. Donovan reported receiving grants from the NIHR during the conduct of the study. David E. Neal reported receiving grants from Cancer Research UK during the conduct of the study. Freddie C. Hamdy reported receiving grants from the NIHR (ProtecT trial chief investigator) during the conduct of the study and receiving personal fees from Intuitive Surgical, Recordati, Pfizer, and Ipsen outside the submitted work. No other disclosures were reported.

## Supporting information


**Appendix S1.** The ProtecT trial supplement.

**Table S1.** Site‐specific distribution of RP specimens that were available for centralised histological review as part of this study. The notably higher representation of specimens from the Cambridge site is attributed to the fact that the centralised review was led by the Cambridge‐based research team. This provided the investigators with direct access to local pathology archives for retrospective retrieval of whole‐mount slides that had not been made available as part of the original centralisation exercise. It should be noted that no historical pathological data on cribriform morphology (ICC or IDC) was available for the unreviewed specimens, as these features were not part of the original trial's pathology proforma conducted according to 2005 ISUP recommendations.
**Table S2.** Distribution of randomisation characteristics between the ProtecT participants included in this analysis and those whose data were missing from this analysis. Note that the biopsy Gleason score provided in this table was derived from the original proforma using the 2005 ISUP guidelines. It should also be noted that no historical pathological data on cribriform morphology (ICC or IDC) was available for the unreviewed specimens, as these features were not part of the original trial pathology proforma completed according to 2005 ISUP recommendations.
**Table S3.** Sensitivity and specificity of systematic biopsies for assessing binary cribriform status compared to ground‐truth RP histopathological assessment.
**Table S4.** Sensitivity and specificity of systematic biopsies for detecting patients with cribriform‐positive, GG ≥3 and/or pT3b disease compared to ground‐truth RP histopathological assessment.
**Table S5.** Prevalence of cribriform‐positive patients in the treatment‐received cohort with ICC, IDC, or both ICC and IDC in their RP specimens. Please note that given the lack of basal cell immunohistochemistry (IHC) in all cases, the distinction between ICC and IDC was made based on morphological haematoxylin and eosin (H&E) assessment according to the ISUP consensus document. Given the limitations of this approach, these descriptive and hypothesis‐generating results should be interpreted with caution.
**Fig. S1.** Detailed histopathological characteristics. (A, B) Data are from the per‐protocol cohort. (C, D) Data are from the treatment‐received cohort. EPE, extraprostatic extension; GG, Grade Group; GP3‐5, Gleason pattern 3–5; SVI, seminal vesicle invasion. *Note that all patients with GG 1 disease are cribriform‐negative by definition.
