# Supplementary material for: Long‐term outcomes of cribriform‐positive and cribriform‐negative prostate cancer treated with radical prostatectomy in the ProtecT trial
Source: BJU Int. 2026 Mar 27;137(6):984–91. doi: 10.1111/bju.70261 (PMC13168917; doi:10.1111/bju.70261)
Supplement: Supplementary file 1 — Appendix S1. The ProtecT trial supplement. [file BJU-137-984-s001.pdf]

ProtecT trial supplement

This supplement contains the following items:

1. Original ProtecT trial protocol, final protocol, summary of changes.

2. Original ProtecT statistical analysis plan, final statistical analysis plan, summary of changes.

ProtecT study

Prostate testing for cancer and Treatment

Protocol Version 1, September 2001

HTA No 90/26/99

Principal Investigators:

FC Hamdy[1]

JL Donovan[2]

DE Neal[3]

Trial Co-ordinator:

JA Lane[4]

[1]Academic Urology Unit , University of Sheffield, Royal Hallamshire Hospital, Sheffield S10 2JF Tel: 0114 271 2154

[2]Dept of Social Medicine, University of Bristol, Bristol BS8 2PR Tel: 0117 9287214

[3]School of Surgical Sciences, University of Newcastle, Freeman Hospital, Newcastle NE7 7DN Tel: 0191 2843111

[4]Dept of Social Medicine, University of Bristol, Bristol BS8 2PR Tel: 0117 9287335

For advice regarding the conduct of the study contact: Dr Athene Lane, Study Co-ordinator, Dept of Social Medicine, University of Bristol, Canynge Hall, Whiteladies Road Bristol BS8 2PR Tel: 0117 9287335 or 9287248, Fax: 0117 9287292, email: athene.lane@bristol.ac.uk

For advice regarding clinical issues contact: Professor Freddie Hamdy, Academic Urology Unit, University of Sheffield, Royal Hallamshire Hospital, Sheffield S10 2JF Tel: 0114 271 2154, Fax: 0114 2712268, email: F.C.Hamdy@sheffield.ac.uk

Contents

**1**	**Introduction**		4
1.1		Background to study	4
1.2		Benefits to the NHS	4
**2**	**Trial design**		5
**3**	**Aims**		6
**4**	**Objectives**		6
**5**	**Study design**		6
**6**	**Ethical aspects**		6
6.1		Ethics	6
6.2		Ethics Committee Approval	6
6.3		Participant Consent	7
6.4		Investigator responsibilities	7
**7**	**Study population**		7
7.1		Participant enrolment	
**8**	**Inclusion and exclusion criteria**		7
**9**	**Case finding and recruitment**		8
9.1		Recruitment of general practices	8
9.2		Participant invitation procedures	8
**10**	**Prostate check clinic (PCC)**		9
10.1		Initial data collection at the PCC	9
		A. Research nurse	
		B. Participant	
10.2		Consent 3 'Cooling off' form for PSA test	10
10.3		Eligibility for diagnostic phase	11
**11**	**Diagnostic phase**		11
11.1		Procedure for diagnoses of localised prostate cancer	11
11.2		Criteria for localised prostate cancer	11
11.3		Ineligibility for randomisation at the diagnostic phase	11
11.4		Data collection at the diagnostic phase	12
11.5		Biopsy interpretation across centres	12
**12**	**Eligibility appointment**		12
12.1		Data collection at eligibility appointment	13
12.2		Incidentally arising localised prostate cancer	13
**13**	**Information appointment**		13
**14**	**Randomisation**		13
14.1		Minimisation variables	14
14.2		The main study: the three arm-trial	14
14.3		Alternatives to the three arm trial	14
14.4		Data collection at information appointment	14
14.5		Data collection after treatment allocation	14
**15**	**Treatment schedules**		15
**16**	**Active monitoring**		15
16.1		Treatment details	
16.2		Disease progression	
**17**	**Radical prostatectomy**		16
17.1		Treatment details	
17.2		Data collection	
17.3		Histopathological staging and evaluation	
17.4		Positive surgical margins	
17.5		Disease progression	

**18**	**Radical radiotherapy**		17
18.1		Treatment details	
18.2		CT planning for radiotherapy	
18.3		Volumes and dose reference point	
18.4		Organs at risk	
18.5		Simulation procedures	
18.6		Treatment technique	
18.7		Dose computation	
18.8		Dose specifications	
18.9		Treatment verification	
18.10		Data collection	
18.11		Clinical follow-up	
18.12		Disease progression	
**19**	**Recruitment flow**		20
**20**	**Research data collection**		20
20.1		Evaluation of case finding	19
20.2		Adverse events	19
20.3		Research data collection at six months	20
20.4		Research data collection annually	20
20.5		Clinical follow-up	20
20.6		Survival data	20
**21**	**Outcome measures**		21
21.1		Primary outcomes	21
21.2		Secondary outcomes	20
**22**	**Economic evaluation**		21
22.1		Data collection	23
22.2		Analyses	23
**23**	**Qualitative research**		23
**24**	**Data management and security**		24
**25**	**Management committees etc.**		24
25.1		Trial steering committee	24
25.2		Data monitoring and ethics committee	24
25.3		Regional management committee	24
25.4		Departures from protocol	24
25.5		Organisation of study documentation	25
25.6		Study monitoring	26
25.7		HTA monitoring	26
**26**	**Publications**		26
**27**	**Project Milestones**		26
**28**	**References**		27
	**Appendix 1**	Setting up a new clinical centre	
		Training for new centres	
	**Appendix 2**	Sample size and statistical analyses	29
		Additional sample size calculations	
		Planned sub-group analyses	
		Randomised participants	
		Secondary analyses	
	**Appendix 3**	Progression of participants in the study	32

Abbreviations CC = clinical centre, SMed = Dept of Social Medicine, Bristol

1. Introduction

1.1 Background to study

Prostate cancer is a major public health issue. The natural ageing of the population, combined with the continued and widespread use of improved diagnostic tests such as serum prostate specific antigen (PSA), are resulting in an increase in the numbers of men diagnosed with localised prostate cancer. In England and Wales, it is the second most common malignancy in men, with 6,179 new cases registered in 1971, rising to 17,210 in 1993[1]. Screening to identify prostate cancer while it is confined to the gland has provoked much public and scientific attention and there is intense debate about its role in improving men's health. While there are strong advocates of screening, the findings from most reviews of the scientific evidence conclude that there is insufficient evidence to recommend population screening because of the lack of evidence that prostate cancer screening would improve the quantity and quality of men's lives[2-5]. Particular concerns relate to the lack of knowledge about the natural history of screen-detected disease, and the lack of evidence about the effectiveness of treatments. In particular, no survival advantage has been shown for any major treatment, and each can result in damaging complications and outcomes, including incontinence and impotence for radical interventions and anxiety relating to the presence of cancer in "watchful waiting".

There have been several attempts to undertake randomised trials comparing two or more of the main treatments (radical prostatectomy, radiotherapy and watchful waiting), but each has suffered problems. Serious methodological flaws including failure to conduct an intention-to-treat analysis, pre-PSA detection of disease and high drop-out rates mean that it is not possible to rely on the two completed trials[6-8]. In the early 1990s, the UK MRC attempted to establish a trial comparing the three major treatments (PR06), which failed to recruit because of its reliance on incidentally diagnosed participants and the reported unwillingness of participants and clinicians to accept randomisation. A trial is currently underway in the US comparing early radical prostatectomy with observation (PIVOT),[9,10] but is experiencing difficulty in recruiting. There have also been more recent small-scale attempts to persuade participants to be randomised between the major treatments, but these have concluded that randomisation is not acceptable to men with prostate cancer [11,12].

1.2 Benefits to the NHS

Good evidence of treatment effectiveness should be available before there is widespread adoption of invasive treatments with potentially serious side effects. In localised prostate cancer, this is not the case. Despite the lack of evidence that radical treatment of early prostate cancer alters outcome, there is an increasing rate of detection in the general population through opportunistic PSA screening, and more men are offered treatment in the form of surgery and radiotherapy[13]. This represents an increasing burden on NHS resources, and is becoming a serious economic and ethical problem. Decisions are currently made by clinicians who tend to favour radical approaches, with patients who fear the consequences of living with an untreated cancer[13]. While the need for randomised controlled trials is not in doubt, difficulties in mounting such trials called for new methodological approaches which were employed in the Phase I feasibility study – methods which subject the clinical encounter itself to critical scrutiny and incorporates more fully the participant's perspective. The failure of other studies, including the MRC trial PRO6, which closed due to poor recruitment have been noted. There is a widespread view that participants are unwilling to be randomised to a non-radical treatment arm, a view that was shown in the feasibility study to be erroneous.

Currently, there is limited and poor quality evidence on which to base the decision about screening for prostate cancer[14,15]. The case-finding process will also provide much needed information about the

accuracy, acceptability, costs and workload implications of screening tests. There will be opportunities for linked studies such as comparing outcomes with controls (CAP study), and conducting basic science research to develop new methods of detection and treatment (Prompt). The evidence that will emerge from ProtecT and linked studies will influence the management of localised prostate cancer in the UK and world-wide.

2. Trial design

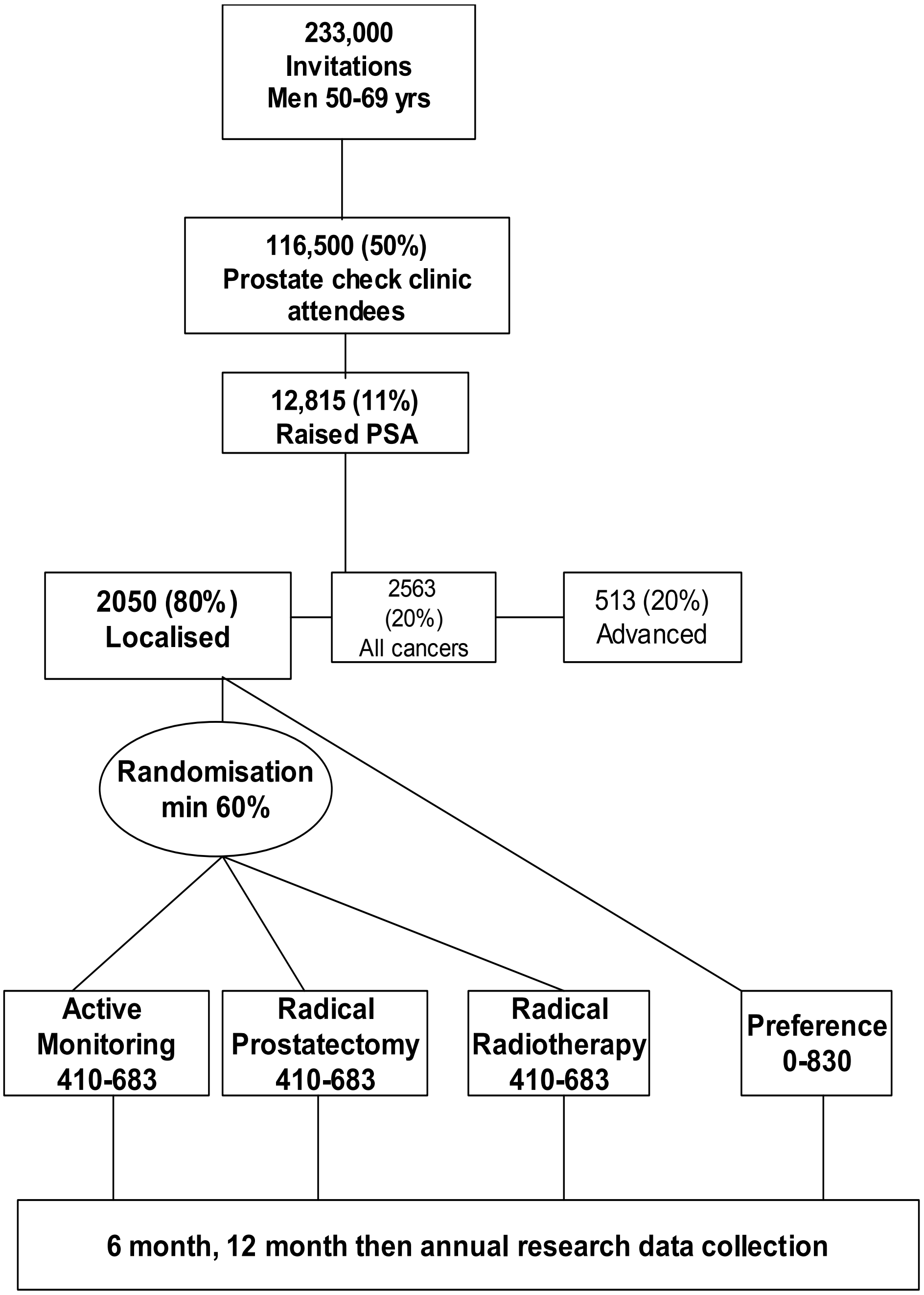

*Randomisation to 2 arm trial of radical treatments is also possible.

3. Aims

To evaluate the effectiveness, cost-effectiveness and acceptability of treatments for men with localised prostate cancer in a pragmatic randomised controlled trial. This trial will compare three treatments (active monitoring, radical prostatectomy and radical radiotherapy).

4. Objectives

1) To assess survival at 10 years and 15 years following treatment
2) To investigate a number of short and medium-term outcomes, including:
   - ◊ 5-year survival
   - ◊ disease progression (biochemical and clinical)
   - ◊ treatment complications
   - ◊ lower urinary tract symptoms
   - ◊ psychosocial impact of case-finding and treatment, including generic health status, quality of life and sexual function
3) To estimate the resource use and costs of case-finding, treatment and follow-up, and to compare costs and outcomes of treatment in terms of survival and health related quality of life.
4) To collect samples suitable for basic science research.

5. Study design

The treatment trial consists of two major components:

1) **Case-finding** - early detection of prostate cancer with participants invited from general practices to attend prostate check clinics to be informed about the uncertainties of treatment and the implications of testing
2) **A three-arm randomised trial of treatment** for participants with early localised prostate cancer

(NB: A 2-arm randomised trial of treatment and patient preference group can also be included)

6. Ethical aspects

6.1 Ethics

The study will be conducted according to the Declaration of Helsinki 1964, as revised in Tokyo 1975, in Venice 1983 and by the 41st World Medical Assembly, Hong Kong, September 1989.

6.2 Ethics Committee Approval (CC)

The principal investigator at each clinical centre (CC) will submit the protocol to the appropriate Local Ethics Committee for approval. The application for approval will include a copy of the participant consents, information sheets and other relevant materials. Approval has already been given by Trent MREC for ProtecT on 21st June 2001.

6.3 Participant Consent (CC)
Persons asked to participate in this research are entitled to choose whether or not to take part. Their decision will be voluntary and they will be competent to understand what is involved. Consent forms will be designed to assure the protection of their rights.

Participants will receive both written and verbal information. The written information has been approved by the medically qualified investigators. The verbal explanation to the participant will be performed by the research nurse under the supervision of the medically qualified investigators. The verbal explanation will cover all the elements specified in the written information provided for the participant. The participants will be informed of the aims, methods, anticipated benefits and potential hazards of the study including any discomfort it may entail.

The participant will be given every opportunity to clarify any points he does not understand and if necessary ask for more information. At the end of the discussion the participant will be given time to reflect. The participant is at liberty to withdraw their consent to participate at any time, without prejudicing their future medical care.

The research nurse will then obtain the participants freely given written informed consent for each stage of the study. Both investigators and participants retain a copies of the signed consent forms.

6.4 Investigator responsibilities (CC)
The principal clinical investigator at each centre will be responsible for the clinical conduct of the study staff. The clinical investigators will maintain a Trial Master File including a list and CVs of appropriately qualified persons to whom they have delegated significant trial-related duties. The investigator will be responsible that all such identified persons will be thoroughly familiar with the protocol and study procedures, as well as being aware of the principles of good clinical practice (GCP).

7. Study population

7.1 Participant enrolment
Participants will be recruited through general practices. They will all be men within the age range 50-69 years. All such persons within the practices will be invited to attend for a PSA test to detect prostate cancer. Those men who have confirmed localised prostate cancer will be invited to participate in the treatment trial.

8. Inclusion and exclusion criteria

Inclusion criteria
- Age 50-69 years on the date of the Prostate Check Clinic (PCC)
- Male gender
- Able to give informed written consent to participate
- Fit for any of the three treatments

Exclusion criteria
This trial is of pragmatic design. Therefore, exclusion criteria will be kept to the minimum possible. Participants will be excluded from entry if they have:
- Concomitant or past malignancies (other than a small treated skin cancer)
- Prior treatment for prostate malignancy

- Serious cardiac problems in the previous 12 months of the PCC, i.e. stroke, MI, heart failure
- Kidney dialyses or transplantation

9. Case-finding and recruitment of participants

9.1 Recruitment of general practices (CC)

Practices randomised in the Comparison Arm of ProtecT (CAP) study to receive ProtecT will be visited by the CAP recruitment coordinator. Dr David Jewell SMed (tel: 0117 928 7356). GPs will be briefed about the ProtecT study, given the protocol and asked for consent for the practice to take part in ProtecT. The lead nurse will subsequently visit the practice to establish suitable accommodation for the study clinics and to liase with practice staff.

9.2 Participant invitation procedures (CC and SMed)

The clinical secretary will go to the participating GP surgeries and download the name, address, date of birth, NHS number and GP practice identification number of all men aged 50-69 years onto the study laptop computer. If possible, the list of men will have been previously screened by the practice for those unsuitable to participate and noted on Access database. All individuals invited to participate in the trial will be allocated a unique study number. Address labels will be generated and invitations to join the study sent to men in manageable batches (Downloading protocol). Letters are mailed out from the general practice on the practice headed notepaper. The data downloaded from the practice computer will only be saved at the practice. As each new practice is visited a record will be made of the doctor's names, address and contact details, computer system and total list size as well as the date of the first invitations. This information will be entered on the clinical databases and copied to the Department of Social Medicine, Bristol (SMed).

The reply slips are returned to Social Medicine and the names and addresses of men indicating their willingness to join the study will be entered on the project database along with the date on which the reply slip was returned. Men who telephone and indicate either their willingness to participate or refusal are recorded in the same way as for letters. Those men who do not reply to the initial letter or who decline to participate on the reply slip will have no follow-up within ProtecT and their details will not be recorded on the main database.

Lists of men who are willing to participate are sent electronically via a secure network (NHSnet) to the clinical secretaries. Secretaries in clinical centres will arrange appointments for these men and manage the prostate check clinic (PCC) lists, including rearranging appointments where necessary. A participant information sheet outlining the study will accompany the appointment letter (Information Sheet 1). The dates and times of the clinics and attendees will also be entered into the study database. Persons who do not/can not attend their intended appointment will be contacted by telephone and a further appointment organised with details recorded on the database. Should they not attend on 2 occasions it will be assumed that they no longer wish to participate and this will be documented on the study database. The PCC lists of appointments and place of the clinics will also be recorded on the database.

10. Prostate check clinics (CC)

Recruitment and case-finding for localised prostate cancer will be performed mostly at the participating general practices, but also at the hospital. On attendance the research nurse (previously carefully instructed by the study team and working from a detailed script utilised in the training programmes) will provide verbal information on the study aims and design. Particular attention will be paid to the treatment phase of the trial. It will be made clear that only those who have localised prostate cancer will be required to participate in the treatment trial. It will be stressed that the treatment is allocated at random by computer, unless the participant refuses randomisation and expresses a preference for one treatment regime.

The clinical significance of prostate cancer will be discussed and it will be made clear that participation in the project is purely voluntary and the participant will be free to decline without prejudicing their future care. Participants will be given the opportunity to ask questions. Those who decline to participate will be free to leave and will be thanked for attending. They can have a PSA test if they so wish. They are notified of PSA results by post and letter posted to their GP (Lett ExcludGP).

The research nurse will ensure that all men willing to participate in the trial are eligible to do so (using the eligibility criteria detailed earlier and in the PCC Schedule). Those ineligible to participate will have the reason for ineligibility explained to them and they will be thanked for their attendance and support of the project. They can have a PSA test if they so wish. They are notified of the PSA results by post and letter is sent to their GP (Letter Excluded GP).

Men who wish to participate will be asked to give written, witnessed consent (Consent Forms 1 and 2). Men have a further 24-hour period after the PCC clinic during which time they must sign and return a further consent form (Consent form 3) to agree to the PSA test being processed. A copy of all signed Consent documents will be given to participants.

10.1 Initial data collection at the Prostate Check Clinic

A. Research nurse

1. Discusses study information, and requests consent to participate in the study (Consents 1 and 2 in Schedule for Prostate Check Clinic).
    - Consent form 1 is for consent to enter the study
    - Consent form 2 is for consent to take blood for the PSA test and future studies, including checking GP or hospital records
    - Consents are given to participant either on the day (*2 copies of consents in future Schedule*), or photocopied and posted with PSA results
    - A cross is placed in boxes of sections the participant does not consent to, initialised by the nurse

2. Completes the Schedule for Prostate Check Clinic containing:
    - baseline socio-demographic data; age, socio-economic status, ethnicity
    - baseline clinical data, e.g. previous urinary problems or PSA tests
    - exclusion criteria checklist
    - a checklist to discuss with participants describing the study

3. Completes a single page version of the data entry form (PCC Patient Summary Sheet) about the attendance at the clinic and outcome. The PCC sheet is entered onto the study computer at the earliest opportunity by the clinical secretary. Any potential problems with conducting a biopsy e.g.

allergies to penicillin, warfarin etc. are written on the reverse of the sheet as are any other comments regarding the man or the appointment.

4. Checks the participant's case notes if previous PSA tests have been performed and records the results on the form.

5. Records self-reported height and inside leg measurement of the men on the PCC Schedule and measures their weight, blood pressure and pulse. If the blood pressure is above a hypertensive level agreed with the current practice (identified by the lead nurse in initial visits) the participant will be advised to have the blood pressure checked again and the practice will be informed of the reading. *A box will be marked on the schedule* to indicate the man is classed as hypertensive by the practice guidelines to remind the need to notify the practice as well as the participant.

6. If men consent to the PSA test, the nurses takes blood (Dr Steven Oliver, SMed Tel: 0117 9287296 *FCH to write protocol*). This requires the taking of 40 mls of blood, which should be placed in a cool box during the clinic. 10mls is placed in EDTA, to be transferred to polypropylene vessels for freezing in the laboratory. Indelible markers should be used to write the study id number and the date of birth on the bottle. It is hoped to devise printed labels that can be used on the small tubes. At the end of the clinic, it must be spun and stored within 18 hours. Whole blood and 1-2ml aliquots of serum are stored at -80^{0}C in a logical documented order. The cap of the first bottle in a series of samples should be marked with a cross when they are placed in the boxes to assist location in cold storage.

7. It is envisaged that laminated sheets of relevant sections of the protocol be made for use in clinics.

B. Participants

1. Men complete a study questionnaire (MT1) on urinary symptoms (ICS*male*SF questionnaire[16]), general health status [SF-12[17], Hospital Anxiety and Depression scale[18] (HAD), EuroQol EQ-5D[19]] which they may return in the post if necessary, using a freepost envelope.
2. Men are given a questionnaire and a freepost envelope to complete at home on environmental exposures and prostate cancer, e.g. diet for the PROMPT study.

The questionnaires and schedules will be entered by SMed.

10.2 Consent form 3 ("Cooling off" consent) and notification of PSA results (CC)

Men have a further 24-hour period after the PCC clinic during which time they must sign and return a further consent form (Consent form 3) to agree to the PSA test being conducted on their blood sample. If men agree, the PSA test is conducted and the results entered onto the project database by the research nurse. Participants who telephone are asked to return their consent form by post. Men who do not complete the forms properly are requested to do so by post with the incomplete form posted back (Letter Consent3/retP). If participants do not return this form after being contacted by telephone or letter (Letter Consent3/NRP), or do not consent to PSA testing (Letter Consent3/refP), or do not sign the form, or are ineligible for ProtecT at the PCC, their blood specimens will be destroyed. This information is recorded on the study database.

All participants will receive the test results by post. The majority (~90%) of participants will have a normal PSA result (i.e. <3.0 ng/ml) and will exit the study (Letter NormP). The participant's GP will be informed of the test results (Letter NormGP).

All men with negative PSA results should have their ProtecT study data returned to SMed for data entry and storage at this stage, i.e. PCC schedule, MT1 questionnaire, Consent3, grouped inside PCC schedule and recorded on the front of the PCC schedule.

10.3 Eligibility for diagnostic phase of the study

- Men with a raised PSA result[20] (≥3.0ng/ml) from the PCC test

11. The diagnostic phase (CC)

11.1 Procedure for diagnoses of localised prostate cancer

All men with a raised PSA result (section 10.3) are invited to attend the Urology department of the clinical centre (Letter ab-lowP). The participant's GP will also be informed (Letter ab-lowGP). The dates of the appointment and the attendance will be recorded on the study database.

The Patient Summary Sheet will be reviewed prior to biopsy e.g. for medication and whether to collect additional blood if 40mls were not obtained at the PCC.

At this appointment they will have a:

- TRUS-guided biopsy (10 cores)[21-23] under antibiotic prophylaxis according to local protocols
- physical examination including digital rectal examination (DRE)
- second PSA test (however, it is emphasised that for the trial subsequent action will be taken on the basis of the PCC PSA test)

11.2 Criteria for localised prostate cancer (CC)

Men with clinically localised disease (T1-T2, NX, M0) defined according to the 1997 TNM classification[21] are eligible to participate in the treatment trial. Staging will be performed by the clinician involved, according to local protocols.

If the PCC PSA is 10-19ng/ml an isotope bone scan is performed to exclude advanced metastatic disease and have a second biopsy without prior free/total PSA testing if biopsy 1 is negative.

All diagnostic tests and staging must be completed before the eligibility appointment, preferably within 4 months from the PCC date.

11.3 Ineligibility for randomisation determined at the diagnostic phase (CC)

1. Men with high PSA measurements (≥20ng/ml) will be dealt with urgently by the urologist, outside the ProtecT study, and the GP informed (Letter ab-highGP). Those found to have advanced disease are treated according to conventional practice, and exit ProtecT. A letter is sent to the participant's GP (Letter AdvanGP). *Information on disease grade and staging will be required for the CAP and PROMPT studies for all those with cancer who are ineligible for ProtecT.*
2. Men with skeletal metastases shown by the bone scan will be excluded (LetterAdvanGP).
3. Men with high grade prostatic intraepithelial neoplasia (HGPIN) are offered a repeat biopsy immediately, as ~50% will have an associated invasive prostatic adenocarcinoma[24]. A letter is sent to the participant's GP.
4. Men with a negative biopsy will have their free/total PSA ratio measured in Sheffield on a monthly basis samples to be sent on dry ice, preferably Tuesday–Thursday (A Milford Ward tel 0114 271 4566, email, amw@immqas.org.uk) with study centre and idno, name, forename, DOB,

date of sample. Letters are sent to the participant and their GP (Letter neg-biopsy/PSAhighP, Letter neg-biopsy/PSAhighGP).

- Those men with a free/total PSA ratio of <0.12 will be offered an immediate repeat biopsy.[25-26] (Letter neg-biopsy/PSAhighGP–bx, Letter neg-biopsy/PSAhighP-bx)
- Those men with a free/total PSA ratio of ≥0.12 without HGPIN will be given the opportunity to be monitored by PSA testing. A letter is sent to the participants GP and the participant informing them that they do not have prostate cancer, suggesting that following discussion with participant, PSA at 12 mths (PSA1 <10ng/ml) in primary care or 6 mths (PSA1 10-19 ng/ml) in secondary care, with the GP to send results to urologist if they wish.

11.4 Data collection at the diagnostic phase (CC)

The research nurse completes a single page data entry form (Diagnostic Events Proforma) for all men with a raised PSA which is then entered onto the study computer at the earliest opportunity by the clinical secretary. This proforma records the clinical stage and grade of the disease, including Gleason scores, and whether other tests have been performed, e.g. bone scans or additional PSA tests. It is possible that there will be several appointments or events and results e.g. bone scans during the diagnostic phase, and data collection must occur at each appointment or event.

Participants are asked to complete the study questionnaire (Questionnaire MT2) containing similar measures used in the PCC clinic questionnaire (MT1). The questionnaire will be entered by SMed.

If localised prostate cancer is diagnosed and the participant is excluded for any reason e.g. for health grounds, then this must be fully documented.

All men not proceeding to the eligibility appointment for whatever reason should have their ProtecT study data returned to SMed for data entry and storage, i.e. PCC schedule, MT1, MT2 questionnaires, Consent3, diagnostic proforma grouped inside PCC schedule and recorded on the front.

11.5 Biopsy interpretation across clinical centres

Quality control will be ensured by regular exchange of material for cross evaluation and confirmation of biopsy interpretations, led by Pathology Management Committee, expert pathologists from the clinical centres (Dr M Robinson, Newcastle, Dr J Goepel, Sheffield and Dr P Harnden, Leeds).

12. Eligibility appointment for participants with confirmed localised prostate cancer (CC)

Men with confirmed localised prostate cancer are invited to attend an eligibility appointment with the study urologist (Letter DiagP and to their GP, Letter DiagGP). **All diagnostic tests and staging must be completed before the eligibility appointment.** This is a relatively short appointment in which the urologist explains the diagnosis, gives the participant an information sheet (Information Sheet 2) and invites the participant to attend a longer 'information' appointment with the research nurse (or the urologist if the participant requests) up to and no longer than 10 days later (unless the participant requests a delay). The feasibility study has shown that this two-stage process is effective and efficient: men are often shocked by the diagnosis and need time to reflect on this and the advantages and disadvantages of the treatments before the trial randomisation ('information') appointment.

12.1 Data collection at the eligibility appointment (CC)

The urologist completes the eligibility schedule (Schedule for Eligibility appointment), which records the clinical stage of the disease, whether the man is fit for all three treatment options and a checklist of issues discussed with the participants. Even if the participant has a strong preference for treatment an information appointment should be arranged. If the information appointment does not happen, an information schedule is completed recording his preference. The Schedule will be entered by SMed.

The whole consultation is recorded on tape unless the participant declines to allow the taping. At the end of the appointment, the tape is marked with the participants study number and placed in the locked cabinet identified for the study at the clinical centre. These tapes are sent to Lucy Brindle, Training co-ordinator (SMed) with the questionnaires and schedules on a regular basis. The tapes are booked in, transcribed and stored in a locked filing cabinet in SMed.

12.2 Incidentally arising cases of localised prostate cancer

Men with an established diagnosis of clinically localised prostate cancer, who present to the clinician through conventional appointments could be offered inclusion into the trial. The clinician involved should explain the principles of the study, and engage them into accepting an information appointment, after signing an initial consent form. Information appointment should be tailored to these patients, as they would have not received the same amount of information as standard ProtecT patients.

NB This will be subject to additional ethical approval to be obtained for the participant information materials.

13. Information appointment (CC)

The main purpose of this information appointment is to provide the participant with sufficient information to allow him to decide whether or not he is willing to join the main trial:

The randomised controlled trial of active monitoring versus radical prostatectomy versus radical radiotherapy (3 arms).

The nurse-researcher emphasises the need for a trial of treatments, describes the advantages and disadvantages of each of the three treatments and explains the purposes of randomisation. The information content and delivery has been determined by the feasibility study and the nurse will work to a detailed but flexible script. Men should not undergo randomisation unless they are able to view all treatments as reasonably equivalent and at that stage randomisation should then proceed. The men can directly request the two arm trial of surgery and radiotherapy if they are unable to view the active monitoring equivalently to the radical arms, but evidence from the feasibility study shows that this should be uncommon if the men are fully informed. The two arm trial is not used for the primary intention to treat (ITT) statistical analyses for the study (Appendix II).

14. Randomisation (CC and SMed)

The participants give their written consent to be randomised (Consent form 4) and a copy of the form is given or posted to the participant (NB Consent 4 is not the acceptance of treatment allocated from the randomisation). Once the participant has given written consent to be randomised, the nurse will telephone SMed (Dan Dedman, Data Manager, Tel: 0117 9287272 or Lead Secretary, Andrea Wilson, 0117 9287248) for the treatment allocation which will be performed in SMed.

14.1 Minimisation variables

- Participants' age on the date of attendance at the PCC (and DOB for confirmation)
- Gleason score (<7 or ≥7)
- Average result of PCC and 1st Biopsy PSA tests (<10 or ≥10 ng/ml)

The participant is told the allocation and asked whether he accepts it. He may want time to think or to talk to other clinicians.

14.2 The main study: the three-arm trial

The primary intention in the information appointment is to recruit informed participants to the main three-arm trial comparing active monitoring, radical prostatectomy and radical radiotherapy.

14.3 Alternatives to the main three-arm trial

If the participant refuses the three arm trial from the outset in the information appointment because he does not wish to receive active monitoring, the nurse should attempt to gain consent for randomisation to the two arm trial (radical radiotherapy or surgery) - 'direct' randomisation to the two arm trial. Randomisation is performed as described above.

If the participant is allocated to active monitoring in the three arm trial and refuses the allocation, they can then be offered the possibility of the two arm trial of surgery versus radiotherapy ('indirect' secondary randomisation to the two arm trial). Randomisation is performed as described above.

If randomisation is unacceptable to the participant, a participant-led preference for a treatment option will be reached without randomisation. These participants will be the 'preference group'.

14.4 Data collection at information appointment (CC)

The nurse will complete a Schedule for the Information Appointment which records a checklist of issues discussed with participants, and the decision reached regarding randomisation and the allocation for the participant. If the participant refuses to be randomised this is recorded. If further appointments are required, then the new appointment date is also recorded on the Schedule.

The whole consultation is recorded on tape unless the participant declines to allow the taping. At the end of the appointment, the tape is marked with the participants' study number and placed in the locked cabinet identified for the study at the clinical centre. These tapes are sent to Lucy Brindle, as described in 12.1.

After randomisation or electing a preference for a treatment option, participants will be given a short questionnaire to complete at home and return in a pre-paid envelope.

[JD TO PREPARE Questionnaire MT3]

14.5 Data collection after treatment allocation

All men are asked to be followed up, whichever treatment decision is reached, including preference participants. A letter is sent to the participant's GP indicating his treatment allocation (LetterRCTGP) or option for treatment preference (Letter PrefGP).

All men who have been randomised or have elected a preference should have their ProtecT study data returned to SMed for data entry and storage, i.e. PCC schedule, MT1 and 2 questionnaires, Consent3, Diagnostic proforma, Eligibility and Information schedules recorded on the front of the PCC schedule.

15. Treatment schedules (CC)

All randomised participants will receive a detailed patient information booklet regarding the risks and benefits of the treatment regime to which they have been allocated or have chosen, and the processes involved, including clinical follow-up.

16. Active monitoring

Men undergoing active monitoring will return for an appointment three months after randomisation to refine their plan of management. Only staff connected with the study should undertake these appointments. PSA results, any additional tests or review appointments will be recorded on the treatment schedule at each appointment. There will be an annual check review of participant notes by the lead urologist to sign off the annual review. *The AM protocol may be reviewed by the DMEC.*

16.1 Treatment details

Their personal plan of management will be decided jointly by the participant and urologist or research nurse, but is likely to include:

- PSA every three months in year 1, then 3-6 months at clinical or participant request
- Rapid 'review' appointments in the event of symptoms (urinary or systemic) or unstable PSA
- Digital rectal examination (DRE) at the review appointment conducted by urologist

At each visit, PSA results will be plotted and examined for any evidence of a rise that might indicate disease progression. Other factors that may cause increased levels, e.g. infection will also be investigated. The aim of active monitoring is to detect disease progression as early as possible, preferably while the tumour is still localised, but also to allow those whose disease remains stable to avoid intervention. Progression will be suspected if:

a) Any rise in PSA level >20% between consecutive measures at any time during follow-up will action an immediate repeat PSA to confirm whether this is a real rise or related to the variability of PSA measurement and a further measure three months later to determine the pattern of rise. Participant to be informed of confirmatory PSA and reassured that it will be reviewed in 3 months. If still raised 3 months later, a 'review' appointment will be arranged.

b) Any rise in PSA level of 50% or greater in any 12 month period confirmed by repeat tests will require a 'review' appointment with the study urologist to discuss the implications of the rise and treatment options.

c) Any indication of the appearance of symptomatic systemic disease will also necessitate a 'review' appointment with the study urologist to discuss the implications and treatment options.

16.2 Disease progression and review appointment

If progression is suspected, the participant will be re-staged. In the 'review' appointment, the study urologist or research nurse will review the pattern of PSA levels in absolute and relative terms, and consider the participant's clinical stage and the disease grade. Participants will be fully informed

about their disease grade, clinical stage and the treatment options, risks and possible outcomes. A decision about treatment will be based on full participant information and joint decision-making.

Treatment options for those with clinically confined disease will include:

- Remaining on active monitoring
- Election for radical surgery or radiotherapy

Treatment options for those with disease no longer confined to the prostate gland will include:

- Remaining on active monitoring with no immediate intervention, but fast-track treatment of any related symptoms that may occur, for example transurethral resection of the prostate for bladder outflow obstruction, hormonal manipulation and/or radiotherapy for painful metastases
- Electing for early hormone ablation and continued monitoring

17. Radical prostatectomy

Participants undergoing radical prostatectomy will be listed for surgery optimally within 2 weeks, and no longer than 2 months, unless specifically requested by the participant for personal reasons.

Accurate per-operative data will be collected, including details of any complications. Standards of performance by the surgeons will be reviewed continually by the steering group.

17.1 Treatment details

Participants will have the procedure explained in detail, as well as possible morbidity and complications. Participants will be admitted 24 hours prior to the surgery, which will only be performed by the surgeons involved in the trial. The steering group will ensure that these surgeons have a high level of expertise in performing the procedure by auditing retrospectively the results of their last 25 cases prior to involving them in the study. Alternatively, surgeons may elect to visit a centre of excellence in either Europe or USA to receive training. Pelvic lymphadenectomy and radical prostatectomy will be performed following the conventional anatomical retropubic approach as described by Walsh[27]. The decision to undertake a nerve-sparing operation will be at the discretion of individual surgeons, depending on individual cases and after discussion with the participants. Prospective collection of data, outcome, rates of complications and results will be used for continuous monitoring of the quality of surgery performed. This is to ensure that the other treatment options are compared with the best possible surgical outcome. During surgery, participants will have their node status assessed:

a) Those with a PSA less than 10 ng/ml and a Gleason score <8 will undergo pelvic lymphadenectomy and radical prostatectomy.

b) Those with a PSA of 10 ng/ml or more and/or a Gleason score of 8 or over will undergo a frozen section biopsy of the pelvic lymph nodes prior to prostatectomy. If the lymph nodes are positive, the participant's further management will be at the surgeon's discretion.

17.2 Data collection

Accurate operative details will be recorded using the Radical Prostatectomy Schedules (Surgeon) and (Researcher), including:

length of the operation, blood loss, technical difficulties, unilateral or bilateral neurovascular bundle preservation, intra-and per-operative complications, the presence of urinary leaks or bleeding post-

operatively, length of hospital stay and occurrence of any immediate post-operative complications, as well as general recovery from the surgery.

The participant will be discharged home and re-admitted 1-2 weeks later for trial without catheter. Continence will be assessed accurately at this stage, and if satisfactory, the participant will be allowed home, for further clinical follow-up 3 months later.

17.3 Histopathological staging and evaluation

This will be performed in a unified manner, with collaboration between respective histopathologists in the centres involved, using conventional tissue handling and histopathological criteria. Quality control will be ensured by regular exchange of material for cross evaluation, led by expert uro-pathologists from the lead clinical centres (Dr MC Robinson, Newcastle, Dr J Goepel, Sheffield).

17.4 Positive surgical margins

This will be defined following conventional histopathological criteria. The association with capsular invasion and seminal vesicle involvement will be documented carefully. Adjuvant treatment (radiotherapy *and/or* hormonal manipulation) for these participants will be at the discretion of individual surgeons after discussion with the participant, and will be guided by PSA levels after the surgery.

17.5 Disease progression during follow-up

Biochemical progression will be defined as serum PSA rising to 0.2 ng/ml or more (on 3 consecutive readings) after being undetectable post-operatively. If progression is diagnosed, a transrectal ultrasound and biopsy of any suspicious local recurrence will be undertaken, as well as an isotope bone scan. If local progression is confirmed by biopsy, adjuvant local radiotherapy will be offered. If distant metastases are confirmed, adjuvant androgen ablation will be offered. In both scenarios, participants will also be offered monitoring following discussion about the risks involved and possible outcomes, particularly in view of the potential long latent period of time before the appearance of clinical progression.

18. Radical radiotherapy

Participants undergoing radiotherapy will receive 3-D conformal external beam radiotherapy. Radiotherapists with a special interest in uro-oncology will be responsible for this treatment, and their results audited retrospectively before and prospectively throughout the trial. Dr David Dearnaley (Academic Unit of Radiotherapy at the Institute of Cancer Research, London) is on the study TSC, and will critically appraise progress of treatments administered within the trial.

18.1 Treatment details

All participants will be treated using 3D conformal methods. Participants will not be eligible to receive prostate brachytherapy, either as sole treatment or as a boost following external beam treatment because this treatment is as yet unproven.

18.2 CT Planning requirements for radiotherapy

CT planning scan should be done 4 weeks before the commencement of radiotherapy. Participants will be treated in the supine position. The bladder will be comfortably full, (participant to drink about 750 mls 1 hr pre-scan) and the participant should be asked to empty the rectum as free of faeces and flatus as possible. No oral, rectal or intravenous contrast should be used. Positioning/immobilisation will be using current departmental methods. Reproducibility of the positioning of the participants will

be maintained using orthogonal laser beams or an equivalent methods. The tumour, clinical and planning target volumes will be defined on CT scans, which will be taken at 5 mm intervals (4 mm slice thickness). Scans will be taken from the bottom of the sacro-iliac joints to the penile urethra (usually 1 cm below ischial tuberosities will be adequate).

18.3 Volumes and dose reference point

These will be outlined on CT scans taken in the treatment position as above. Outlining should be done at least 12 (not necessarily contiguous) CT slices, so that beam portal may accurately conform to the shape of the prostate, plus or minus seminal vesicles. The gross tumour volume (GTV) can be accurately defined on CT images, the clinical target volume (CTV) and planning target volume (PTV) are more difficult to define accurately and computer generated region growing algorithms are recommended to define the required margins between 1 and 1.5 cm. Volumes will be defined according to the ICRU report[28].

Two groups of participants will be defined:

- **Group L** (low risk of seminal vesicle involvement)

  clinical stages T1b/c or T2a with (PSA + [(*Gleason score –6) x 10]) <15
- **Group M** (moderate or high risk of seminal vesicle involvement)

  clinical stages T1b/c or T2a with (PSA + [(*Gleason score –6) x 10]) $\geq$15 and clinical stage T2b

Gross tumour volume (GTV) will be defined on the basis of clinical and radiological staging as 1) either prostate and base of seminal vesicles or 2) to include prostate and all of the seminal vesicles. No deliberate attempt will be made to include lymph nodes, as adjuvant lymph node irradiation has not been shown to be beneficial.

Using a 6 field plan it has been found that the 80% isodose (i.e. total dose 72 Gy) gives about 0.5 cm margin around the GTV. To minimise the rectal volume included in phase II boost vol $GTV_2 = CTV_2 = PTV_2$.

		Phase I	Phase II
**Group L**	**GTV**	Prostate & base SV (GTV_1)	Prostate only (GTV_2)
	**CTV**	GTV_1 + 0.5*	GTV_2
	**PTV**	CTV_1 + 0.5-1.0#	GTV_2
**Group M**	**GTV**	Prostate & SV (GTV_1)	Prostate only (GTV_2)
	**CTV**	GTV_1 + 0.5*	GTV_2
	**PTV**	CTV_1 + 0.5-1.0#	GTV_2

*margin to allow for macroscopic tumour spread, #margin to allow for accuracy of treatment planning and delivery process which will be specified by each centres individually

18.4 Organs at risk

Normal tissues outlined will include bladder, rectum and femoral heads together with the body contour. The normal tissues will be outlined and considered as solid organs. Bladder should be outlined from base to dome. The rectum should be outlined from the anus taken at the level of the ischial tumerosities **or** 1 cm below the lower margin of the PTV, whichever is more inferior, to the

recto sigmoid junction. This will give length of approximately 12 cm in most cases. Any additional bowel in the treated volume should be outlined separately.

18.5 Simulation procedures

All treatment fields should normally be simulated for phase 1 standard dose treatments. After simulation the shape of the multileaf collimator (MLC) leaves or cerroband blocks should be indicated on simulator films or DRRs. The phase II radiotherapy boost will be standardly be given with a 6 field technique (but 4 fields may be used when utilising cerroband blocks). In the simulated position of the isocentre should be determined using orthogonal anterior and lateral fields. The lateral field will be of the same dimension as used in the treatment plan, the anterior will be of the same length as the 6 treatment fields but will be standardised at a width of 10 cm (no contrast materials are required during simulation).

18.6 Treatment technique

Phase I: 3-field techniques should use anterior and left and right posterior oblique, or left and right lateral fields. 4-field techniques should use anterior/posterior and right and left lateral fields.

Phase II: The radiotherapy boost will standardly be given with a 6 field technique using left and right anterior oblique, left and right posterior oblique and left and right lateral fields when treatment is delivered using an MLC. The angle of the oblique fields will usually be at 35-40^0 from the lateral beams. Alternatively, a 4-field technique using anterior/posterior and right and left lateral bands can be used with cerroband blocks.

No area in the rectum of bladder outside the PTV should receive more than 74 Gy, and the maximum dose per fraction is 2 Gy (i.e. < or + 100% isodose).

18.7 Dose computation

Three dimensional dose distributions should be produced. Beam's eye view representations of PTV and organs at risk will be reproduced for each treatment beam and additionally in the mid-axial plane. If there is marked variation in participant contour further axial distributions should be obtained 2 cm from the cranial and caudal field edges. Ideally, a mid-plane sagital dose distribution should be produced.

18.8 Dose specification

Dose prescription to participants will be whenever possible >70 Gy in 2 Gy fractions to be defined at the isocentre. Participants will then receive a further 10Gy in 5 fractions to the phase II boost volume again defined at the isocentre. All fields will be treated daily on a linear accelerator of 5 MeV or greater. The planned overall treatment time will be 7.5 weeks. For participants receiving treatment using an MLC a maximum delay of 5 treatment days may be permitted during therapy to allow for technical difficulties. If for technical reasons a delay for longer than this period is likely a maximum of five treatments may be given with unshaped fields. If it is likely that there will be longer periods of delay then shape blocks will be made. MLC or shaped blocks must be used for all boost treatments.

Minimum and maximum (area of at least 2 cm^2) dose within the defined PTV would normally be 95% and 105% respectively. Hot spot dose outside the PTV will not exceed 105%. Dose to organs at risk outside the PTV will not exceed the prescribed dose to the isocentre. Cumulative dose to the femoral heads should not exceed a maximum dose of 55 Gy to and area of $\geq$ 2 cm^2.

Dose corrections will be made for the femoral heads either on a pixel by pixel or using standardised value of bone density.

Dose volume histograms evaluating dose to GTV, PTV and organs at risk (rectum, femoral heads and bladder will be collected wherever possible).

18.9 Treatment verification

Portal or check films will be taken during treatment according phase I (3 or 4 field). When portal imaging devices are available daily images will be taken during week 1 and thence at weekly intervals. When using film at least 2 images will be taken during the fist week of treatment. The six field boost treatment will be verified using the lateral port and an orthogonal anterior film to ensure accuracy and position of the isocentre.

Port films will be compared to simulator images (or digitally reconstructed images from CT). Treatment accuracy to within 2-3 mm is to be obtained whenever possible and positioning errors of 5 mm and greater are unacceptable. Corrections of participant positioning and appropriate resimulation will be employed if errors greater than this magnitude are apparent before the next radiotherapy fraction is delivered.

18.10 Data collection

The Radiotherapy Schedule (radiotherapist) and Radiotherapy Schedule (researcher). Quality Audit to be devised in conjunction with Dr D Dearnaley.

18.11 Clinical follow-up

Participants will be seen one month after completion of treatment, and thereafter 3-monthly for the first year, 6-monthly for the second year and then annually thereafter until disease progression (see below).

18.12 Disease progression

Disease progression will be defined as in the ASTRO guidelines i.e. 3 consecutive rises in PSA level, each at least one month apart[29].

19. Recruitment flow

Each centre can see approximately 240 PCC attendees per month when the centre is fully established. 22 participants each month will have a raised PSA (11%) and will require a biopsy. 4 localised cases of prostate cancer will be identified per month (48 per year) and will be eligible for randomisation and follow-up.

20. Research data collection and clinical follow-up (CC and SMed)

All participants diagnosed with localised prostate cancer will be undergo research data collection (comprehensive cohort principle). This includes those who were randomised, chose a preference for a treatment arm, as well as those who sought therapies outside the three treatment arms offered by ProtecT, e.g. brachytherapy, and who are treated in private practice.

20.1 Evaluation of case finding

Records will be kept of the response rates and the accuracy of the tests at each stage of the study. The positive predictive value of the PSA test will be calculated using histological confirmation as the 'gold standard'. The numbers and specific tests required by men with initially abnormal PSA levels (e.g.

confirmatory PSA, TRUS with or without biopsy, bone scan) will be carefully documented to evaluate the urological workload caused by case-finding, including those ineligible for randomisation.

20.2 Adverse events (CC)

Adverse events including treatment complications resulting from any of the three treatment arms will be recorded for each participant by the nurse on a Adverse Events proforma for entry into the computer database. The proforma will list events that could be experienced (e.g. blood clots, rectal or bowel injury or symptoms, infection, death) whilst also allowing the nurse to add comments on other effects and relevant issues.

20.3 Research data collection at six months (SMed)

Research data collection will take place 6 months after the date of the first information appointment, involving a postal questionnaire on resource use as well as the instruments used at baseline on anxiety and depression, urinary symptoms, sexual function and treatment related quality of life.
Questionnaires (MT4) will be posted to participants after any changes of address or contact status are reviewed on the database.

20.4 Research data collection at 12 months, and annually (CC)

Full research data collection will take place at 12 months, and thereafter annually, after the date of the first information appointment.

Participants will be seen by one of the study nurses who will complete the Researcher 12 month or Annual Data Collection schedule based on the notes from the database and hospital notes as well as the participant interview. Participants will complete a version of the study questionnaire (MT5), similar to that completed at six months.

20.5 Clinical follow-up (CC)

Clinical follow-up will take place at 3-monthly intervals in the first year, and at clinical discretion thereafter. Clinical follow-up will be delivered by the specialist undertaking the delivery of the treatment arms. These appointments will involve assessment of response to treatment, management of any complications, and investigation of any apparent disease progression. Any clinical follow-up is recorded as an event on the study clinical database.

20.6 Survival data

All men participating in the study will be flagged at the UK Office of National Statistics Central Register to ensure that the primary outcome of the study, time ascertained at 10 years, can be analysed. Notification of mortality and cancer incidence amongst study participants will be achieved through flagging and through links with hospital pathology and clinical services in the ProtecT catchments and participating GP practices. Clinical centre staff will return to the general practices to obtain the NHS number of those men who attended the PCC to allow automated flagging.
Notifications from NHSCR will be entered in the Dept of Social Medicine on the study database.

21. Outcome measures

21.1 Primary outcome

The primary outcome is survival time up to 10 years following the first information appointment.

21.2 Secondary outcomes

- Short-term outcomes (6 months/one year): disease progression; treatment complications; urinary symptoms, quality of life, sexual function, and anxiety, depression and other psychosocial effects
- Medium-term (5 years): as above, plus survival
- Long-term (10 years & 5 yearly thereafter): as above, plus survival

These outcomes will be evaluated in the following ways:

1. Survival – a small group will be convened before each major analysis of survival to scrutinise death certificates and investigate/confirm the true cause of death. [*Blinded to treatment received]*
2. Disease progression - using PSA, DRE, ultrasonography, biopsy, bone scans.
3. Treatment complications –immediate and delayed treatment complications including blood loss, rectal/bowel injury/symptoms, sexual dysfunction, urethral stricture, incontinence, and concerns about living with an untreated cancer will be collected in clinical schedules and participant questionnaires developed in the feasibility study.
4. General health status - measured by validated instruments: the SF-12, a subset of the SF-36[17], and EuroQol EQ-5D[18].
5. Anxiety and depression - measured by the Hospital Anxiety and Depression Scale[18].
6. Urinary symptoms - measured by the ICSmaleSF questionnaire[26], which includes voiding and incontinence scores, nocturia, frequency and urinary-specific quality of life.
7. Sexual function - measured by the ICSsex questionnaire[30].
8. Quality of life related to prostate cancer treatment – measured using the Functional Assessment of Cancer Therapy (FACT) with the prostate cancer subscale[31].
9. Qualitative evaluation of outcome - assessed by in-depth interviews with samples of participants in each arm of the trial and also the preference groups.
10. Resource use (NHS, social service and personal). Routine hospital and primary care data sources with additional questions in clinical and participant questionnaires.

22. Economic evaluation (SMed and Manchester)

The economic evaluation will be led by Dr Linda Davies, Reader and Director in Health Services Research, Manchester Royal Infirmary. The economic evaluation will be conducted from the societal viewpoint as costs associated with the treatment and care of cancer participants may fall on participants, carers, social services and society in general, as well as on the NHS. The evaluation will also be performed using a long run perspective: this is most appropriate to any change in national practice. In this trial, all participants will essentially be receiving a higher level of care than would be usual practice. The aim within this trial is not to determine the efficiency of these treatments relative to current practice nor to determine the efficiency of prostate cancer treatment relative to other forms

of health treatment. Rather, the aim is to provide an internal comparison of the three forms of treatment and to assign costs to the case finding process.

The precise form of the economic evaluation will depend upon the outcomes of the trial. Initially outcomes of the alternative forms of treatment will be compared and consideration will be given to performing a cost-effectiveness or cost-utility analysis. If, for example, there are differences only in survival, a cost-effectiveness analysis will be performed using years of life gained as the measure of outcome. If there are, additionally, differences in quality of life then a cost-utility analysis will be performed, with Quality-Adjusted Life-Years (QALYs) being used as the measure of outcome. These will be formed by combining information about survival with the EuroQol EQ-5D data collected annually, and participant utilities/willingness to pay data. The economic evaluation will be conducted for each of the one year, 5-year and 10-year main study assessments.

22.1 Data collection

During the trial, the direct costs falling on health services, participants, carers and social services as a result of treatment will be identified and collected. Although case-finding costs will be the same in all arms of the trial, and are therefore not relevant to the decision about which treatment to perform, information about the costs associated with case-finding will undoubtedly be useful to policy makers and will therefore be collected during the study. Physical resource use information collected will include: hospital stay, staff time, consumables, diagnostic tests, drugs, capital equipment, GP time and travel, participant and carer travel, out-of-pocket expenses, and any use of social services. Information about the indirect costs and benefits associated with time lost, from both work and leisure, will also be collected. These indirect costs will be presented separately. Routine information systems will be used wherever possible to collect information about both hospital and community services resource use. Resource and cost data from published literature and observational data sources will also be collected to assess whether there are differences between the trial population and routine practice. Where routine systems are available, resource use data will be collected for all participants. Where routine data systems are not available a combination of participant-held diaries and participant and carer questionnaires will be used to assess resource use on a sample of participants from across the centres over the recruitment period. Wherever possible, unit cost data generated within the hospital will be used to value resource use. Pro-rata salary will be used to value staff time. Unit costs of health and social services will be used as a source for the valuation of community/primary care services[32]. Time lost from work will be valued on the basis of average wages, lost leisure time will be evaluated at a proportion of time lost from work.

22.2 Analyses

The analysis from the viewpoint of society will not include any transfer costs/payments. Discounting will be undertaken at 6% (with the discount rate varied during the sensitivity analysis). The economic data collected as part of the trial will be analysed to assess the mean costs, survival and health related quality of life for the specific trial population over the timeframe of the trial (1, 5, 10 and 15 years). This will give a reliable estimate of the relative value for money of the different treatments for the specific population, trial centres and trial protocol. A sensitivity analysis will be undertaken (particularly given that much of the data will be collected in a somewhat artificial trial situation) and attention will be given to generalising the results obtained beyond the trial.

23. Qualitative research (SMed)

The qualitative studies will be co-ordinated by Lucy Brindle (tel: 0117 9287362)

- development and implementation of training methods, including tape-recording of information appointments and rapid feedback to ensure high levels of randomisation
- detailed study of men's experiences of undergoing each of the treatments
- case studies of 10 men from the PCC to the final diagnoses evaluation of the implementation and acceptability of the active monitoring treatment programme
- views and perceptions of urologists participating in the study in conjunction with Dr Gavin Daker-White (University of the West of England)

subject to additional ethics approval:

- *assessment of the best form and delivery of information for incidentally diagnosed men*
- *reasons of men for refusing to participate in case finding*

24. Data management and security

A unique file identified by the study number will be maintained for participants. All data recorded on paper relating to the participant will be located in these files. A list will be maintained at each centre of staff with authorisation to make alteration to the study records, including the study database.

Data obtained on paper will also be entered onto and maintained on a 'Microsoft Access' database. Information capable of identifying individuals and the nature of treatment received will be held in the database with passwords restricted to ProtecT study staff. Data from computerised sources will be converted to 'Access' databases and hard copies will be maintained in the relevant participants file e.g. PSA results, in locked filing cabinets. Information capable of identifying participants will not be removed from SMed or clinical centres or made available in any form to those outside the study. Data moved electronically from clinical centres to the SMed will only be sent by secure NHSnet networks and encrypted.

25. Management and ethical considerations

A Trial Steering Committee and a Data Monitoring and Ethics Committee will oversee the ProtecT trial. Written records will be taken of each meeting and copies held by the study coordinator.

25.1 Trial Steering Committee

- Independent chair (Professor M Baum, London)
- Dr D Dearnaley (clinical oncologist/radiotherapist, London)
- Dr J Adolfsson (external urologist, Sweden)
- Dr P Albertsen (external urologist, USA)
- Dr M Robinson (uro-pathologist, Newcastle-upon-Tyne)
- Professor K Woods (HTA Commissioning Board)
- Principal investigators (Professors Hamdy, Donovan, Neal)

- ProtecT study senior statistician (Dr T Peters, Bristol)
- ProtecT study Coordinator (Dr A Lane, Bristol)
- Observers from the NCCHTA, National Screening Committee and the office of the National Cancer Portfolio Director

If the CAP project is fully funded, Professors F Schröder and S Frankel from the CAP Project will join the TSC to discuss any common issues arising.

25.2 Data Monitoring and Ethics Committee (DMEC)

- Independent chair (Professor A Grant, trialist, Aberdeen)
- Professor P Fayers (statistician, Aberdeen)
- Professor Killian Mellon (academic urologist, Leicester)
- Dr Michael Sokal (expert oncologist/radiotherapist, Nottingham)

The DMEC will be convened at any point when there are questions of safety or ethics in any part of the trial and will be the only body responsible for instigating an interim analysis of study data. They will review the safety and disease progression of participants in each treatment arm and preference participants. Recommendations from the DMEC regarding the stopping rules for the study will be taken to the TSC for ratification. The DMEC will meet every six months, commencing 6-months from the first TSC meeting. A major meeting of the DMEC is planned at the time of the analysis of the average 5-year follow-up data. The TSC can invite the DMEC Chair to attend the TSC if deemed appropriate.

25.3 Regional Management Committees

Each hub centre (Universities of Bristol, Newcastle and Sheffield) will have a regional management committee comprised of the principal investigator based at that hub centre, urologists, radiotherapists, pathologists and lead nurses for each of centres associated with that hub. Dr Steven Oliver, Prodigal study coordinator will also attend the Bristol committee. Regular meetings (about every 3 months) of these committees will assist in monitoring the progress of the study at each centre. Written records will be maintained of these regional meetings and a copy sent to the study co-ordinator.

25.4 Departures from protocol

It is important to keep participant withdrawals from the trial to a minimum but;

- a participant may be withdrawn from the study by their general practitioner or the study team at any time should it be considered detrimental to the participant to continue.

- a participant may withdraw from the study at any time without prejudice to his subsequent treatment.

Participants who fail to attend appointments will be contacted by telephone and letter, to encourage them to attend, to arrange alternative appointments and to determine reasons for withdrawal. Reasons for withdrawal will be fully documented on the study database and adverse event forms completed if applicable.

If participants switch to different treatment arms post randomisation this will be analysed by a per protocol secondary analyses. If data is missing on participants, a sensitivity analyses will be conducted, but this is less powerful than having the correct data.

25.5 Organisation of study documentation

All clinical centres will have an investigators' Trial Master File which will include all relevant information and documentation for the trial. This will include the protocol, LREC approval, financial agreements, CVs of all staff involved in the trial, and any correspondence or emails received pertaining to the study. It will be the responsibility of the lead nurse and clinical secretary at each site to maintain this file.

25.6 Study monitoring

The study will be monitored by the study co-ordinator though reports, visits and examination of the study database. Visits to PCC may occasionally be made by the research study team.

25.7 HTA monitoring visits

The HTA will make annual monitoring visits regarding the conduct and progress of the study. The meeting will take place at one of the research hubs. The first meeting is scheduled for the end of 2002.

26. Publications

Brief six monthly reports will be produced for the HTA. Papers will be prepared for publication in general and urological peer-reviewed journals. The findings will also be presented at national and international conferences. The primary analyses will be undertaken when there is an average 10-year follow-up (i.e. end of year 13). During the study, a number of other publications are expected, including five year survival analysis, the effectiveness of the training programme on randomisation rates, the accuracy of PSA tests, urological workload in terms of confirmatory tests required following PSA testing, short-and medium-term outcome following each of the treatments.

27. Project milestones

YEAR 1

- Continue full-scale recruitment in Sheffield, Newcastle and Bristol
- Train three new centres from September 2001 to March 2002 (Birmingham, Cardiff, Edinburgh)
- Train three new centres from March to September 2002 (Leeds, Leicester and Middlesbrough) if requirements are complied with, e.g. availability of 3-D conformal radiotherapy
- Initiate 6-monthly meetings of steering group to evaluate recruitment, co-ordination between centres and data quality control. To continue throughout length of study
- Follow-up of all participants to be continued throughout lifetime of study

YEAR 2

- Continue full-scale recruitment in Sheffield, Newcastle, Bristol and three centres commencing in year 1, with increasing recruitment in the next three centres

YEARS 3 TO 5

- Continue full-scale recruitment in all nine centres – to be completed at the end of year 5
- Analysis of short-term outcome in terms of psychosocial factors, urinary symptoms, sexual dysfunction, rates of treatment complications and quality of life in the treatment groups

28. References

1. Majeed A, Babb P, Jones J, Quinn M. *BJU Int* 2000; 85:1058-1062
2. Wasson JH, Sushman CC, Bruskewitz RC, et al. *Arch Fam. Med* 1993;2:487-93.
3. Editorial. UK experts advise against prostate cancer screening. *Lancet* 1997;349 (9050):1-2.
4. Woolf SH. *BMJ* 1997;314:989-90. 1993;71:375-7.
5. Neal DE, Donovan JL. *Lancet Oncology* 20001: 17-24..
6. Paulson DF, Lin GH, Hinshaw W, Stephani S. *J Urol* 1982;128:502-5
7. Graversen PH, Nielsen KT, Gasser TC, et al. *Urology* 1990;36:493-8
8. Madsen P, Peder H, Gasser TC, Corle DK. *Scand J Urol Nephrol* 1988; 110: 95-100
9. Wilt TJ, Brawer MK. *J Urol* 1994;152:1910-4
10. Wilt TJ, Brawer MK. *Semin Urol* 1995;13:130-6
11. O'Reilly P, Martin L, Collins G. *BMJ* 1999 Jun 5;318(7197):1556
12. Livesey,JE; Cowan,RA; Brown,CW. Clinical Oncology 2000; 12:63
13. Donovan JL, Frankel SJ, Faulkner A, Selley MA, Gillatt D, Hamdy FC. *BMJ* 1999; 318:299-300
14. Chamberlain J, Melia J, Moss S. *NHS R&D HTA Programme: Health Technology Assessment*, 1997, 1 (3)
15. Selley S, Donovan JL, Faulkner A, Coast J, Gillatt D. *NHS R&D HTA Programme: HTA*, 1997; 1 (2)
16. Donovan JL, Peters TJ, Abrams P, Brookes ST, De La Rosette JJ, Schafer W. *J Urol* 2000;164:1948-55
17. Brazier JE, Harper R, Jones NMB, et al. *Br Med J,* 1992; **305**:160-164.
18. Zigmond AS, Snaith RP. The Hospital Anxiety and Depression Scale. *Acta Psychiat Scan,* 1983; **67**:361-
19. The EuroQol Group. EuroQol - a new facility for the measurement of health... *Health Policy* 1990; 16:199-208.
20. Schröder FH, van der Cruijsen-Koeter I, de Koning HJ, et al. *J Urol* 2000 Mar;163(3):806-12
21. Babaian RJ, Toi A, Kamoi K, et al. *J Urol* 2000;163:152-7
22. Presti JC Jr, Chang JJ, Bhargava V, Shinohara K. *J Urol* 2000;163:163-6; discussion 166-7
23. Borboroglu PG, Comer SW, Riffenburgh RH, Amling CL. *J Urol* 2000;163:158-62
24. Hermanek P, Hutter RVP, Sobin LH et al*, TNM Atlas. Fourth edition*. UICC: Springer, 1997
25. Häggman MJ, Macoska JA, Wojno KJ, Osterling JE. *J Urol* 1997; 158: 12-22
26. Catalona WJ, Partin AW, Slawin KM, et al. *JAMA* 1998 20;279:1542-7
27. Patel D, White PA, Milford Ward A. *BJU Int* 2000 Apr;85(6):686-9
28. Walsh PC. *Campbell's Urology*. Ed. Walsh PC, Retik AP, *et al*. WB Saunders. Philadelphia. pp 2865-2886
29. Lahtinen T, Tenhunen M, Vayrynen M. *Radiother Oncol* 1993 Aug;28(2):174-6.
30. Consensus statement: guidelines for PSA following radiation. *Int J Rad Onc Biol Phys* 1997 15;37(5):1035-41.
31. Frankel SJ, Donovan JL, Peters TJ et al. *Journal of Clinical Epidemiology*, 1998; 51 (8): 677-685.
32. Esper P, Mo F, Chodak G et al. *Urology* 1997; 50 (6): 920-8.
33. Netten A and Curtis L. *Unit costs of health and social care, 2000.* PSSRU, University of Kent, 2000.

34. Garcia-Closas M, Lubin JH. *Am J Epidemiology* 1999; 149: 689-92
35. Lubin JH, Gail MH. *Am J Epidemiology* 1990; 131: 552-66

APPENDIX 1: Setting up a new clinical centre

Resources have been provided by the NHS HTA Programme to support all research costs. Each clinical centre will be sub-contracted to one of the major research hubs (Newcastle, Sheffield and SMed). Resources will be obtainable by quarterly invoice in arrears to the hub centres. First wave centres will commence in September 2001; second wave in March 2002.

1. Lead nurse and secretary to be appointed as close to day 1 as possible. The lead nurse will be employed by the research hub, but all other staff will be appointed through the Trust.
2. Appointments and setting up of study to be assisted by the close involvement of the lead nurses from the research hubs (Peter Holding {PH}, Sheffield or Teresa Mewes {TM}, Newcastle). Assistance will also be provided by the ProtecT training co-ordinator, LB (SMed).
3. Clinical centre's lead nurse (CCLN) to shadow PH or TM in the research hub for two days. Birmingham, Cardiff, Leicester and Leeds to Sheffield; Edinburgh and Middlesbrough to Newcastle.
4. CCLN to identify first practice, set up lab staff/procedures, TRUS/biopsy clinics, office procedures etc.
5. CCLN to schedule first prostate check clinic (PCC) appointments hourly in first instance. Two days' worth, then stop for discussion with PH or TM.
6. Two-day training programme for new nurses at SMed, new secretaries and urologists if possible.
7. Two new WTE 'F' grade nurses to be appointed by month 3. 0.5 WTE secretary also to be appointed. 1 WTE 'F' grade nurse to be appointed by month 9 to help with follow-up and PCCs.
8. LB, PH, TM, AL, Jenny Donovan (JD)and Freddie Hamdy (FH) to work with urologists and lead nurses over the first few months to ensure they are aware of the study details, budget arrangements and provide training for the eligibility appointments and the information appointments where participants request second opinions.
9. PCC appointments will be extended to 45 minutes during the training period. A minimum of 60 appointments per week, each lasting approximately 30 minutes, is expected when the centre is working at full capacity (i.e. after six months).

Training new centres for the information appointment and randomisation

Full training for nurses and urologists will be based on the findings of the feasibility study. It is expected that at least 70% of participants will consent to randomisation following training. Training will include:

1. Initial 2-day course outlining the study procedures, need for a treatment trial, evidence about treatments, concepts and practicalities of randomisation, and practice in the delivery of the study information.
2. Observing information appointments led by training coordinator nurses in the research hubs (Sheffield and Newcastle) - 'mentoring'.
3. Consenting to the tape-recording of 'information' appointments and for these to be studied by (LB)/(JD).
4. Receiving feedback and further training based on the analysis of the tape-recordings.

5. Accepting that the randomisation rate will be monitored and consenting to further tape-recordings and feedback during the progress of the study.

The training programme developed by JD, FH, LB and AL is based on the feasibility study. The training team will consist of JD, FH, LB, PH, TM and AL.

If the randomisation rate is <60% after the first 6 months, every effort will be made by the ProtecT study team to increase recruitment to the acceptable rate required for the study sample size (minimum 60%). If this rate can not be reached an alternative centre will have to be found to replace the centre.

APPENDIX 2: Sample size calculations and statistical analyses

The latter part of the feasibility study showed that each centre can see approximately 200 prostate check clinic attenders and thus detect approximately 4 localised cases per month (48 per year). The table below indicates the numbers of eligible cases expected based on five years of recruitment in nine centres in total (i.e. six new centres in addition to the current three), assuming that each new centre will require six months of training and will operate at 0.5 efficiency over first 12 months.

Centres	Feasibility	Year 1	Year 2	Year 3	Year 4	Year 5
Current 3	150	144	144	144	144	144
4+5+6		72	144	144	144	144
7+8+9			100	144	144	144
Total	150	216	388	432	432	432
Cum.	150	366	754	1186	1618	2050

Sample size could be considered in terms of survival time or the proportion expected to survive after 10 years' follow-up. Although the former is preferable since it corresponds to the primary analysis, given the high proportions surviving for 10 years the power of these two approaches is virtually the same. Given the availability of data in the literature on the 10-year survival and the greater transparency of such specifications, the following calculations are therefore presented in these terms. Evidence suggests that 10-year survival is around 85% for all treatments. To demonstrate equivalence between the treatments with two-sided 5% alpha, we have 85-90% power to rule out differences of between 7.5% and 8% with 400 randomised to each arm. The power implications of our expected sample sizes are displayed in the tables below, assuming two different rates of randomisation (two-sided 5% alpha):

(a) Assuming 60% randomised

Recruitment by	Total cases expected	Randomised per arm	Power to rule out difference of	
			8%	7.5%
End year 4	1618	320	80	75
Mid year 5	1834	367	85	81
End year 5	2050	410	89	85

(b) Assuming 70% randomised

Recruitment by	Total cases expected	Randomised per arm	Power to rule out difference of	
			8%	7.5%
End year 4	1618	377	86	81
Mid year 5	1834	427	90	86
End year 5	2050	478	93	90

Additional sample size implications

An analysis for safety purposes is planned for the DMEC at the end of year 8, when there will be an average of 5 years of follow-up for all recruited men. We assume that survival is likely to be around 95% at this time. With 410 in each arm (60% randomised), we will have 85-90% power to rule out differences of 4.5-5.0%. With 478 in each arm (70% randomised), we will have 90-95% power to rule out differences of 4.5-5.0%. A (secondary) analysis of complete follow-up at 5 years is planned for the end of year 10. If we assume that survival is around 90% at this time, with 410 or 478 in each arm we will have 80-85% and 85-90% power, respectively, to rule out differences of 6.0-6.5%. It should be borne in mind that while in absolute terms these differences are smaller than those for the primary analysis, they are relatively large in relation to the proportion surviving overall. Thus, as would be expected, the analysis at 5 years will be less sensitive, but important for safety and monitoring purposes as well as clinical interest.

The primary analysis of follow-up is planned to occur at the end of year 13 when an average of 10 years' follow-up will be available (see power calculations above). An analysis of complete follow-up at 10 years will occur at the end of year 15.

Planned subgroup analyses

It is also important to consider the implications of the sample size on essential *a priori* sub-group analyses: disease grade, age, PSA levels and clinical stage. The most crucial of these relates to disease grade based on Gleason score (the sum of Gleason grading from the two highest grade biopsy cores). Data from the feasibility study indicate that within this participant group with localised disease, between 60% and 70% will have low grade (Gleason score ≤6), and 30-40% high grade (Gleason score 7+) disease. We explore the power implications at the end of the full five year recruitment, with the range reflecting the possible randomisation rates[34,35].

In addition, we give the power if we were to continue recruiting to the end of seven and nine years. Our assumptions are for an analysis based on average 10 year follow-up: no overall treatment effects (i.e. the odds ratio of dying is 1), 10 year survival in low grade disease is 90%, 10 year survival in high grade disease is 75% (i.e. the odds ratio of dying is 3 for high grade compared with low grade disease). Powers given in the table are for an interaction odds ratio of 2, comparing a combination of the radical treatments with monitoring (i.e. effectively a 2:1 randomisation ratio) with presumed mortality rates following radical treatments of 16% and 14.5% (60% and 70% respectively) for the low grade group. This is partly to maximise power but also because it is addressing the most important question: is survival particularly worse in the monitored high grade group?

	Recruitment		
Gleason grade	Five years	Seven years	Nine years
60% low grade<br>40% high grade	60-67%	76-82%	86-91%
70% low grade<br>30% high grade	55-62%	70-77%	81-87%

This implies that for reasonable power, this analysis requires at least seven years' recruitment. If we were to continue to recruit over this time period, an analysis of 10 year average follow-up could occur at the end of year 14.

Randomised participants

All comparative primary analyses of the randomised men will be conducted on an intention-to-treat basis, comparing the three groups as randomised. For the primary analysis of survival (for an average of 10 years' follow-up), Cox proportional hazards regression will be used to obtain hazard ratios and confidence intervals, adjusting for stratification/minimisation. Pairwise contrasts will be corrected for multiple comparisons where necessary.

Secondary analyses

Secondary analyses will include Cox regression for time to disease progression and logistic regression for survival at 10 years. Adjustments will also be made for major imbalances between the arms by introducing appropriate covariates into the regression models. Planned subgroup analysis will be conducted by stratified analyses for descriptive statistics and formally by including interaction terms in the relevant regression models. Further secondary analyses will involve per-protocol analyses by taking into account any departures from the original allocated treatment. Secondary analyses will also explore the impact of inaccurate clinical staging prior to treatment for observational studies comparing the treatment options. Clinical staging is acknowledged to be inaccurate, with approximately 25% of cases found not to be localised to the prostate when full operative staging is carried out. We will thus have the most accurate (pathological) staging only in one treatment arm (radical surgery), but it is likely that similar levels of upstaging will be occurring in the other arms.

Appendix 3: Progression of participants through ProtecT study

Note: X is an exit point, participant does not continue in the ProtecT study.

Participant states pre-PCC

Returns invitation letter or telephones

1. Yes
2. No = X
3. Refuses = X

PCC list of appointments

1. Attends
2. Did not attend, rebook and then put as refusal after 2 times = X
3. Refuses = X

States at end of PCC clinic

1. Refuse Consent form 1 points 1-4 = X and blood destroy if PSA done, once men have result
2. Refuse Consent 2 blood tests = X and blood destroy if PSA taken done, once men have result
3. Refuse Consent 2 points 2 /3, continue in ProtecT, but flag not use blood for other studies
4. Ineligibility = X and blood destroy if PSA taken once men have result
5. Eligible and consent 1 and 2 obtained

States after return of Consent form 3

1. Refuses consent = X
2. Consent given for PSA test
3. Exit ProtecT study as negative PSA = X
4. Eligible for diagnostic phase as raised PSA

NB 1-3 return all participants questionnaires and schedules to SMed inside PCC Schedule

States after diagnostic phase

1. Localised prostate cancer, PSA 3-19ng/ml and fit to continue
2. Participant refusal to attend biopsy
   appointment, rebook 2x , if no = X
3. Advanced cancer = X
4. HGPIN alone on biopsy = X
5. Biopsy negative, no HGPIN, PSA free/total <0.12, 2nd biopsy = clinical and patient decision

NB: Some of these men will have prostate cancer on subsequent biopsies. The decision to offer more biopsies will depend on repeat PSA, and at the discretion of the clinician with a fully informed patient. If they have localised prostate cancer later, they can be included in ProtecT.

6. Biopsy negative, no HGPIN, PSA free/total ≥0.12 = X
7. PSA 10-20ng/ml, bone scan, metastases = X
8. PSA 10-20ng/ml, bone scan, localised
9. Ill-health exclusion = X
10. Other exclusion = X

NB 2-10 return all participants questionnaires and schedules to SMed inside PCC Schedule

States after Information appointment

1. Randomised to three arm
2. Randomised to two arm trial directly
3. Randomised to three arm, refuses AM, randomised to 2 arm
4. Randomised to three arm, refuses and expresses preference
5. Preference option, no randomisation
6. No decision, further appointment(s) arranged

NB if remains undecided for 3 months from date of first information appointment, call AM as preference *de facto*, could still be randomised at later date, and proceed to follow-up as for AM.

7. Withdrawn = X

NB 1-7 return all participants questionnaires and schedules to SMed inside PCC Schedule

Practice close-out

a) On completion of each practice, SMed and CC will audit data to ensure completeness of data records for those participants

b) Practice will receive a summary for men from that practice in terms of numbers of raised PSA, cancers detected of localised and advanced nature etc.

c) Data once cleaned from a practice can be archived and stored off–site or scanned

ProtecT study

Prostate testing for cancer and Treatment

Protocol v 5.5

28th February 2022

ISRCTN20141297

HTA No 96/20/99

Principal Investigators:

FC Hamdy[1]

JL Donovan[2]

DE Neal[3]

Trial Co-ordinator:

JA Lane[4]

[1]University of Oxford, Nuffield Dept of Surgery, Oxford OX3 9DU Tel: 01865 617126
[2]Bristol Medical School, University of Bristol, Bristol BS8 2PR Tel: 0117 9287214
[3]University of Oxford, Nuffield Dept of Surgery, Oxford OX3 9DU Tel: 01865 617126
[4]Bristol Medical School, University of Bristol, Bristol BS8 2PR Tel: 0117 9287335

© Copyright: The University of Oxford and Oxford University Hospitals NHS Foundation Trust 2016

Study Title: The ProtecT trial – Evaluating the effectiveness of treatments for localised prostate cancer

Internal Reference Number / Short title: ProtecT trial

Ethics Ref: MREC 01/4/025

Date and Version No: 28/02/2022, 5.5

Chief Investigator: Professor Freddie Hamdy, University of Oxford, Nuffield Department of Surgical Sciences, John Radcliffe Hospital, Headington, Oxford, OX3 9DU tel: +44 1865 617123; fax: +44 1865 617125; email: Freddie.Hamdy@nds.ox.ac.uk

Investigators: Co-Principal Investigators:
Professor Jenny Donovan, University of Bristol, Population Health Sciences, Bristol Medical School, 39 Whatley Road, Bristol, BS8 2PS; Tel: +44 117 3314599; email: Jenny.Donovan@bristol.ac.uk.
Professor Emeritus David Neal, University of Oxford, Nuffield Department of Surgical Sciences, John Radcliffe Hospital, Headington, Oxford, OX3 9DU; tel: +44 1865 617126; email: david.neal@nds.ox.ac.uk.

Trial Co-Ordinator:
Professor Athene Lane, University of Bristol, Population Health Sciences, Bristol Medical School, 39 Whatley Road, Bristol, BS8 2PS;; tel: +44 117 9287335; email: athene.lane@bristol.ac.uk.

Sponsor: University of Oxford

Funder: NIHR Health Technology Assessment Programme

Chief Investigator Signature:

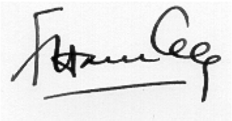

There are no conflicts of interest.

Confidentiality Statement

This document contains confidential information that must not be disclosed to anyone other than the Sponsor, the Investigator Team, HRA, host organisation, and members of the Research Ethics Committee, unless authorised to do so.

© Copyright: The University of Oxford and Oxford University Hospitals NHS Foundation Trust 2016

CONTENTS

1. SYNOPSIS 5
2. ABBREVIATIONS 6
3. BACKGROUND AND RATIONALE 7
   3.1 Background to Study and Results of 10 Year Analyses 7
   3.2 Benefits to the NHS 8
Actual trial recruitment and randomised numbers (Fig 1b) 10
5. AIMS 11
6. OBJECTIVES 11
7. STUDY DESIGN AND METHODS 11
8. ETHICAL ASPECTS 12
   8.1 Ethics 12
   8.2 Initial Ethics Committee Approval (CC) 12
   8.3 Participant Consent (CC) 12
   8.4 Investigator responsibilities (CC) 12
9. STUDY POPULATION AND PARTICIPANTS 13
   9.1 Participant Enrolment 13
10. INCLUSION AND EXCLUSION CRITERIA 13
11. RECRUITMENT OF PARTICIPANTS 13
   11.1 Recruitment of General Practices (CC and Bristol) 13
   11.2 Participant Invitation Procedures (CC and Bristol) 14
   11.3 Participant Visit Schedule (CC) 14
   12.1 Initial Data Collection at the Prostate Check Clinic (PCC) 15
      A. Research Nurse 15
      B. Participants 16
   12.2 Consent Form 3 ("Cooling Off" Consent) and Notification of PSA Results (CC) 16
   12.3 Eligibility for Diagnostic Phase of the Study 17
13. THE DIAGNOSTIC PHASE (CC) 17
   13.1 Diagnosis of Localised Prostate Cancer 17
   13.3 Criteria for Trial Eligibility when PCC Or Biopsy PSA is 10-19.99ng/ml 18
   13.4 Criteria for Trial Eligibility when PCC PSA is >19.99ng/ml 19
   13.5 Data Collection at The Diagnostic Phase (CC) 19
Eligible for ProtecT trial 20
   13.6 Detection of Localised Cancer at Biopsies Subsequent to Monitoring in Primary Care 21
   13.7 Biopsy Classification and Interpretation across Clinical Centres 21
14. ELIGIBILITY APPOINTMENT FOR PARTICIPANTS WITH CONFIRMED LOCALISED PROSTATE CANCER (CC) 21
   14.1 Eligibility Criteria for Randomisation (CC) 22
14.2 Data Collection at The Eligibility Appointment (CC) 22
15. INFORMATION APPOINTMENT (CC) 22
16. RANDOMISATION (CC AND BRISTOL) 23
   16.1 Minimisation Variables 23
   16.2 The Main Study: the Three-Arm Trial 23
   16.3 Alternative to Randomisation 23
   16.4 Data Collection at Information Appointment (CC) 23
   16.5 Data Collection after Treatment Allocation 24
17. TREATMENT (INTERVENTION) PROTOCOLS (CC) 24

© Copyright: The University of Oxford and Oxford University Hospitals NHS Foundation Trust 2016

18. ACTIVE MONITORING....24
18.1 Treatment Details and Follow-Up Pathway....24
18.2 Assessing PSA Changes over Time....25
18.3 Review Appointment and Annual Bone Scans....25
19. RADICAL PROSTATECTOMY....26
19.1 Treatment Details....26
19.2 Data Collection....27
19.3 Histopathological Staging and Evaluation....27
19.4 Positive Surgical Margins....27
19.5 Follow-Up Pathway....27
20.1 Treatment Details....28
20.2 CT Planning Requirements for Radiotherapy....29
20.3 Volumes and Dose Reference Point....29
20.4 Organs at Risk....30
20.5 Simulation Procedures....30
20.6 Treatment Technique....30
20.7 Dose Computation....30
20.8 Dose Specification....30
20.9 Treatment Verification....31
20.10 Quality Assurance and Data Collection....31
20.11 Follow-up Pathway....32
21. RECRUITMENT FLOW....33
22. RESEARCH DATA COLLECTION (CC AND BRISTOL)....33
22.1 Evaluation of Detection (CC and Bristol)....33
22.2 Adverse Events (CC)....34
22.3 Pathology Findings (CC)....34
22.4 Follow-Up Timescale and Participant Group....34
22.5 Research Data Collection of Treatments....34
22.6 Participant Data Collection at Six, 12 Months and Annually....35
22.7 Research and Other Data Collection at 12 Months and Annually (CC)....35
22.8 Survival Data....36
22.9 Extended Research Follow-up from 2018 to March 2022 & COVID-19 amendments....36
22.10 Extended Research Follow-up from April 2022 to March 2027....37
23. CLINICAL FOLLOW-UP (CC)....38
24. OUTCOME MEASURES....38
24.1 Primary Outcome....38
24.2 Secondary Outcomes....38
25. ECONOMIC EVALUATION....39
25.1 Data Collection....40
25.2 Analyses....40
26. QUALITATIVE RESEARCH....40
28. MANAGEMENT, MONITORING AND STUDY ORGANISATION....41
28.1 Trial Steering Committee 2012....43
28.2 Data Monitoring Committee (DMC 2012)....44
28.3 Regional Management Committees....44
28.4 Study Management Committee Meetings....44
28.5 Specialist Sub-group Meetings....44
28.6 Management Executive Committee....45

© Copyright: The University of Oxford and Oxford University Hospitals NHS Foundation Trust 2016

28.7 Departures from Protocol 45
28.8 Organisation of Study Documentation 45
28.9 Study Monitoring and SMART 46
28.10 HTA Monitoring Visits 46
29. PUBLICATION POLICY 46
30. QUALITY ASSURANCE PROCEDURES 46
31. ETHICAL AND REGULATORY CONSIDERATIONS 47
In extended follow up from 2018 toMarch 2022 47
31.1 Declaration of Helsinki 47
31.2 Guidelines for Good Clinical Practice 47
31.3 Approvals 47
31.4 Reporting 47
31.5 Participant Confidentiality 47
31.6 Expenses and Benefits 47
32. FINANCE AND INSURANCE 47
32.1 Funding 47
32.2 Insurance 48
33. PROJECT MILESTONES 48
year 1 48
year 2 48
years 3 to 5 48
APPENDIX 1: SETTING UP A NEW CLINICAL CENTRE 51
Training new centres for the information appointment and randomisation 52
APPENDIX 2: SAMPLE SIZE CALCULATIONS AND STATISTICAL ANALYSES (2012) AND UPDATED FOR EXTENDEND FOLLOW-UP (2018 TOMarch 2022) 53
Sample size implications 53
Data analysis plan 54
Primary analysis 54
Secondary analyses 55
Description of Statistical Methods (2018 toMarch 2022) 56
The Number of Participants 57
Analysis of Outcome Measures 57
APPENDIX 3: PROGRESSION OF PARTICIPANTS THROUGH PROTECT STUDY 58
APPENDIX 4: AMENDMENT HISTORY 60

FIGURES

Figure 1: Trial Design Flowchart 9
Figure 2: The Clinical Diagnostic Process Before Eligibility Is Determined 20
Figure 3: Active Monitoring Follow-Up Pathway 26
Figure 4: Radical Prostatectomy Follow-up pathway 28
Figure 5: Radiotherapy Follow-Up Pathway 33

Notes

1. All SOPs and additional protocols are available from the study coordinator
2. Study website: http://www.bristol.ac.uk/population-health-sciences/projects/protect/

© Copyright: The University of Oxford and Oxford University Hospitals NHS Foundation Trust 2016

1. SYNOPSIS

**Study Title**	The ProtecT trial – Evaluating the effectiveness of treatments for localised prostate cancer	
**Internal ref. no. / short title**	The ProtecT trial	
**Study Design**	Randomised Controlled Trial	
**Study Participants**	Around 110,000 recruited between 2001-2009	
**Planned Sample Size**	Around 2,600 in extended follow-up	
**Planned Study Period**	Until 31/03/2027 to include a 20- year median analysis	
	**Objectives**	**Outcome Measures**
**Primary Objective**<br>Definite or probable prostate cancer specific mortality (and intervention-related mortality) at a median of 20 years following randomisation	Ascertainment by independent cause of death committee using vignettes compiled from medical note reviews and routine data	Ongoing<br><br>On notification of death
**Secondary Objectives**<br>At a median of 20 years		Ongoing (unless stated otherwise)
Overall survival	Death certificate data	On notification of death
Disease progression and metastasis<br><br>Treatment complications	Indicators of disease progression and complications of treatment from routine NHS data (PHE). Verification by clinical record reviews where necessary	Routine data
General health status, quality of life, symptoms (urinary, sexual etc), depression and psychological state	Validated PROMs for symptoms/treatment complications: ICSmaleSF, ICIQ, EPIC; cancer-specific QoL: EORTC QLQC-30 and overall QoL: EQ-5D-3L	Posted annually to participants or completed online up to 15 years' individual follow up or December 2022 when completed
Qualitative evaluation of outcome	In-depth interviews with sample of participants in each arm and preference groups	Completed
Cost-effectiveness of treatments	Resource use from hospital and primary care and participant questionnaires	Completed
Samples for basic science research (ProMPT) study	Annual blood sample (c. 50 mls) at NHS prostate cancer appointments	Completed

© Copyright: The University of Oxford and Oxford University Hospitals NHS Foundation Trust 2016

2. ABBREVIATIONS

CC	Clinical Centre
CI	Chief Investigator
CRF	Case Report Form
CTRG	Clinical Trials & Research Governance, University of Oxford
GCP	Good Clinical Practice
GP	General Practitioner
HRA	Health Research Authority
ISD	Information Services Division Scotland
MRC	Medical Research Council
MRI	Magnetic Resonance Imaging
NHS	National Health Service
NICE	National Institute for Health and Care Excellence
NIHR	National Institute of Health Research
OU	Oxford University
PCC	Prostate Check Clinic
PCT	Primary Care Trusts
PEDW	Patient Episode Data for Wales
PHE	Public Health England
PI	Principal Investigator
PIL	Participant/ Patient Information Leaflet
PROM	Patient Reported Outcome Measure
PSA	Prostate Specific Antigen
QoL	Quality of Life
R&D	NHS Trust R&D Department
REC	Research Ethics Committee
SOP	Standard Operating Procedure
TSC	Trial Steering Committee
UOB	University of Bristol

© Copyright: The University of Oxford and Oxford University Hospitals NHS Foundation Trust 2016

3. BACKGROUND AND RATIONALE

3.1 Background to Study and Results of 10 Year Analyses

Prostate cancer is a major public health issue. The natural ageing of the population, combined with the continued and widespread use of improved diagnostic tests such as serum prostate specific antigen (PSA), are resulting in an increase in the numbers of men diagnosed with localised prostate cancer. In England and Wales, it is the second most common malignancy in men, with 6,179 new cases registered in 1971, rising to 17,210 in 1993.[1] Screening to identify prostate cancer while it is confined to the gland has provoked much public and scientific attention and there is intense debate about its role in improving men's health. While there are strong advocates of screening, the findings from most reviews of the scientific evidence conclude that there is insufficient evidence to recommend population screening because of the lack of evidence that prostate cancer screening would improve the quantity and quality of men's lives.[2-5] Particular concerns relate to the lack of knowledge about the natural history of screen-detected disease, and the lack of evidence about the effectiveness of treatments. In particular, no survival advantage has been shown for any major treatment, and each can result in damaging complications and outcomes, including incontinence and impotence for radical interventions and anxiety relating to the presence of cancer in "watchful waiting".

There have been several attempts to undertake randomised trials comparing two or more of the main treatments (radical prostatectomy, radiotherapy and watchful waiting), but each has suffered problems. Serious methodological flaws including failure to conduct an intention-to-treat analysis, pre-PSA detection of disease and high drop-out rates mean that it is not possible to rely on the two completed trials.[6-8] In the early 1990s, the UK MRC attempted to establish a trial comparing the three major treatments (PR06), which failed to recruit because of its reliance on incidentally diagnosed participants and the reported unwillingness of participants and clinicians to accept randomisation. A trial is currently underway in the US comparing early radical prostatectomy with observation (PIVOT),[9,10] but is experiencing difficulty in recruiting. There have also been more recent small-scale attempts to persuade participants to be randomised between the major treatments, but these have concluded that randomisation is not acceptable to men with prostate cancer.[11,12]

Updated background (2022)

The management of clinically localised and opportunistic screen-detected prostate cancer remains a highly controversial public health issue. In the UK in 2014, more than 45,000 new cases of prostate cancer were diagnosed, around 300,000 men were living with a prostate cancer diagnosis, and there were over 11,000 deaths.[13]

The NIHR ProtecT trial was designed to evaluate the comparative effectiveness and cost-effectiveness of active monitoring, radical prostatectomy and radical radiotherapy for clinically localized prostate cancer in terms of mortality, disease progression and patient-reported outcomes and experiences. Participants were recruited to ProtecT in nine UK centres between 2001 and 2009.[14] Over 220,000 men aged 50-69 years were invited for testing, 100,444 attended, 3,221 were diagnosed with prostate cancer. Of these, 2,664 had clinically localized disease, of whom 1,643 were randomised, and 997 selected their own treatment. In addition, 267 men with advanced prostate cancer and 290 men excluded from randomisation for other reasons were also followed up and there were also 279 men with a PSA of above 20 ng/ml. The primary outcome, prostate cancer-specific mortality at a median of 10 years' follow-up,[15] was reached on 23rd November 2015, in parallel with completion of a full 6-year follow-up for patient reported outcome measures (PROMs)[16] and a longitudinal qualitative study of men's experiences.

The ProtecT publications at a median of 10 years have had substantial impact and global attention, with over 300,000 downloads to date, 250 citations in less than 12 months, and almost 400 media stories, and included in the 12 most notable NEJM papers of 2016.[17] The clinical outcomes paper was listed as 22nd of 100 most

© Copyright: The University of Oxford and Oxford University Hospitals NHS Foundation Trust 2016

impactful publications in 2016 according to Altmetric.[18] The US Task Force and NICE are now using these findings to revise their recommendations on the diagnosis and management of prostate cancer. There is evidence of a change in clinical practice in the UK, (UK National Prostate Cancer audit (NPCA) https://www.npca.org.uk/about/ and NPCA reports 2014-2019) and through clinical guidelines, clinical practice has changed accordingly worldwide.

The primary outcome, prostate cancer-specific mortality at a median of 15 years' follow-up, was reached on 23rd November 2021, in parallel with completion of 12-years follow-up for patient-reported outcome measures (PROMs). These findings are still confidential, pending submission to a leading journal. However, a meeting with the Trial Steering Committee in January 2022 confirmed these median 15-year results underpin the need for further follow-up for a median of 20 years. This additional follow up of clinical primary and secondary outcomes only (not PROMs) will further extend understanding of the impacts of treatment and provide essential information for policy and treatment decision-making for men newly diagnosed with localised prostate cancer.

3.2 Benefits to the NHS

Good evidence of treatment effectiveness should be available before there is widespread adoption of invasive treatments with potentially serious side effects. In localised prostate cancer, this is not the case. Despite the lack of evidence that radical treatment of early prostate cancer alters outcome, there is an increasing rate of detection in the general population through opportunistic PSA screening, and more men are offered treatment in the form of surgery and radiotherapy.[19] This represents an increasing burden on NHS resources, and is becoming a serious economic and ethical problem. Decisions are currently made by clinicians who tend to favour radical approaches, with patients who fear the consequences of living with an untreated cancer.[19] While the need for randomised controlled trials is not in doubt, difficulties in mounting such trials called for new methodological approaches which were employed in the Phase I feasibility study – methods which subject the clinical encounter itself to critical scrutiny and incorporates more fully the participant's perspective. The failure of other studies, including the MRC trial PRO6, which closed due to poor recruitment have been noted. There is a widespread view that participants are unwilling to be randomised to a non-radical treatment arm, a view that was shown in the feasibility study to be erroneous.

Updated background (2022)

Currently, based on the median 10-year findings from ProtecT, men, clinicians and policy-makers know that a man diagnosed with clinically localised prostate cancer will have a very low risk of dying from prostate cancer (~1%) whether they have radical treatment or active monitoring, but an increased risk of developing metastases with active monitoring compared with surgery or radiotherapy. What we do not know is whether or when this additional rate of developing metastases in active monitoring will be translated into an increased risk of dying of prostate cancer earlier than if they had surgery or radiotherapy, or whether the even higher risk of dying from other causes including cardiovascular disease and other cancers will intervene.

In addition, there were some small (but not significant) differences between surgery and radiotherapy at 10 years in terms of primary treatment failure and biochemical progression which may or may not translate into significant differences in metastases or progression. If this occurs by the median 15-year follow up, this would also change policy and practice. If these small differences favouring surgery turn into significant differences in the analyses with more years of follow-up, then the higher levels of sexual dysfunction and urinary incontinence could form a better trade-off. In active monitoring, there is a gain immediately from avoiding a loss of sex life and retaining continence, without knowing whether the higher risk of metastases will result in a

© Copyright: The University of Oxford and Oxford University Hospitals NHS Foundation Trust 2016

shortening of life expectancy and the impact of the development of metastases and their treatment on quality of life.

The median 15 year analyses will provide patients, clinicians, and policy makers with more information y about the 'trade-off' between oncological outcomes for disease progression and side-effects of treatments in the short- and medium-term, longer follow-up to a median of 20 years is essential to provide definitive information about mortality and disease progression.

Original text: 4. TRIAL DESIGN (Figure 1)

Original trial figure (Fig 1a)

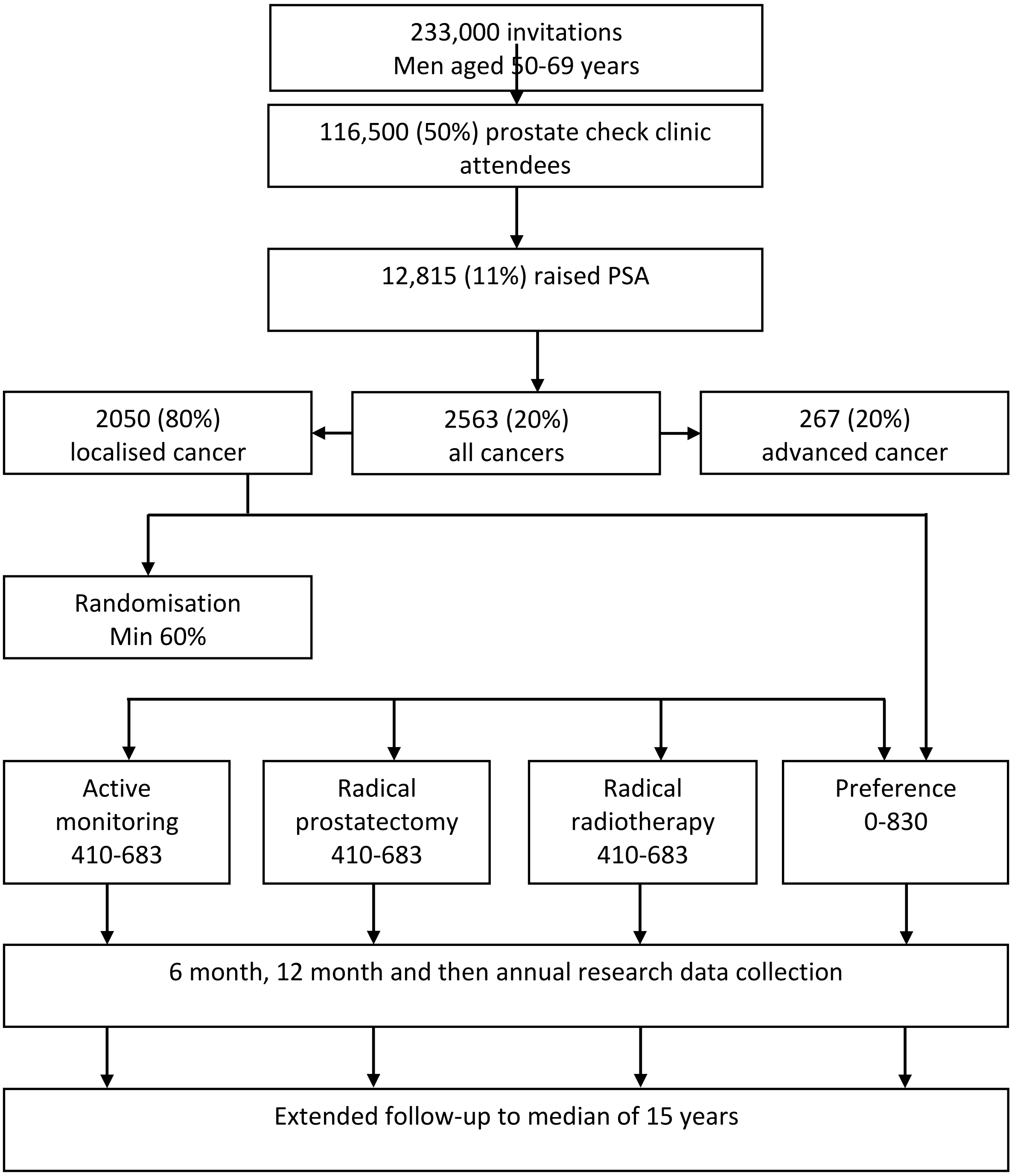

© Copyright: The University of Oxford and Oxford University Hospitals NHS Foundation Trust 2016

Actual trial recruitment and randomised numbers (Fig 1b)

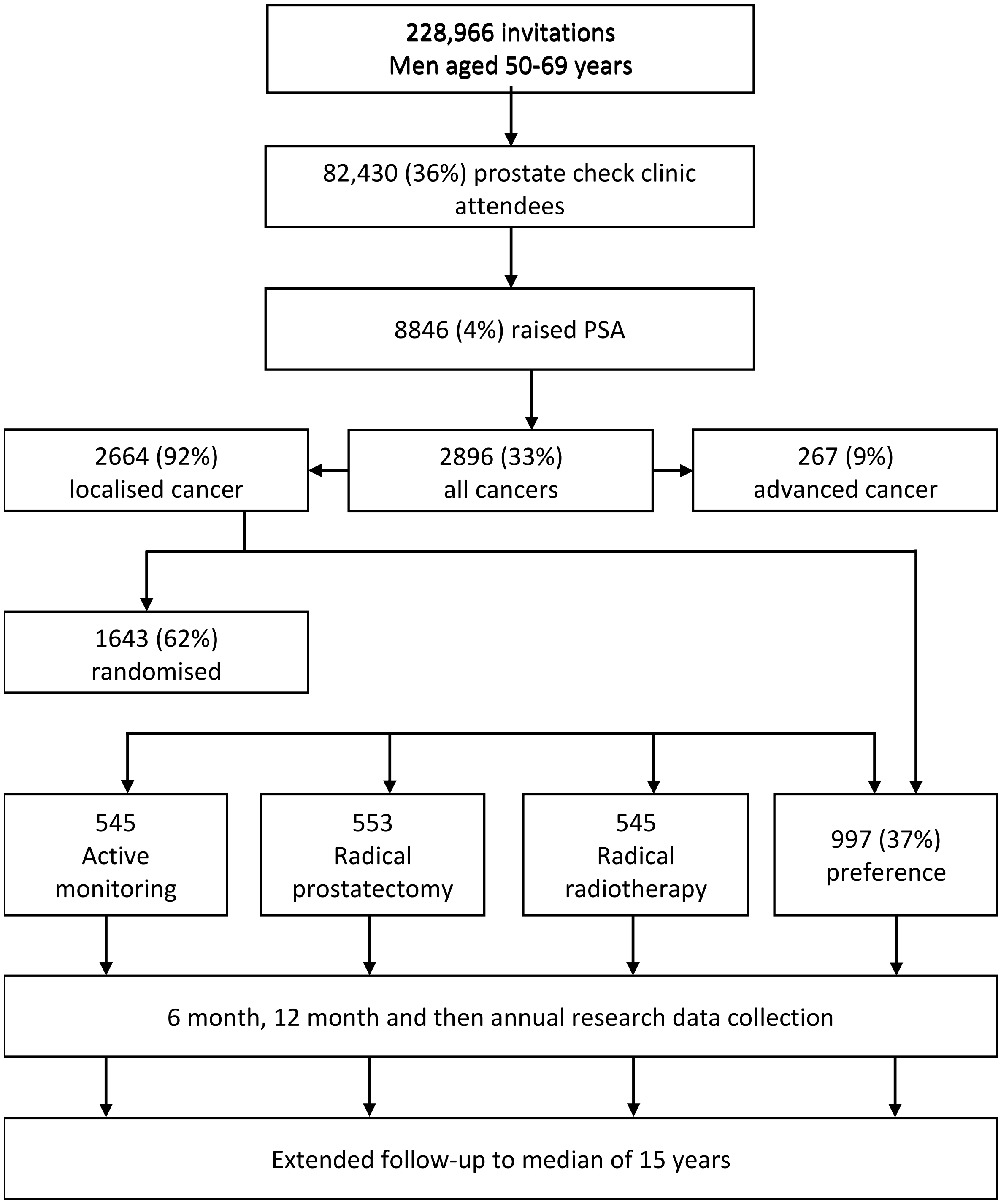

© Copyright: The University of Oxford and Oxford University Hospitals NHS Foundation Trust 2016

5. AIMS

To evaluate the effectiveness, cost-effectiveness and acceptability of treatments for men with localised prostate cancer in a pragmatic randomised controlled trial. This trial will compare three treatments (active monitoring, radical prostatectomy and radical radiotherapy). The cost-effectiveness analysis was completed during the 10-year median analysis in 2016.

6. OBJECTIVES

1) To assess definite or probable prostate cancer specific mortality (including definite or probable intervention related mortality) at a median of 10, 15, and 20 years following randomisation**.**
2) To investigate a number **of secondary outcomes**, including:
   - ◊ overall survival
   - ◊ disease progression (biochemical and clinical)
   - ◊ treatment complications
   - ◊ lower urinary tract symptoms (completed by individual participants up to 15 years)
   - ◊ psychosocial impact of detection and treatment, including generic health status, quality of life and sexual function (completed by individual participants up to 15 years)
3) Completed: To estimate the resource use and costs of case-finding, treatment and follow-up, and to compare costs and outcomes of treatment in terms of survival and health related quality of life up to the 10 year median analysis.
4) Completed: To collect samples suitable for basic science research (ProMPT study).

7. STUDY DESIGN AND METHODS

The treatment trial consists of two major components:

1) Early detection of prostate cancer with participants invited from general practices to attend prostate check clinics to be informed about the uncertainties of treatment and the implications of testing
2) A three-arm randomised trial of treatment for participants with localised prostate cancer

The 10 year median follow-up was published in 2016. Extended follow-up will continue until March 2027 to allow a 20 year median analysis. Follow-up will be predominantly passive (routine data and medical note review) and is described in section 22.10. Participant questionnaires will cease to be collected at the end of 2022.

© Copyright: The University of Oxford and Oxford University Hospitals NHS Foundation Trust 2016

8. ETHICAL ASPECTS

See revised section 30.1

8.1 Ethics

The Investigator will ensure that this study is conducted in accordance with relevant regulations and with Good Clinical Practice.

8.2 Initial Ethics Committee Approval (CC)

The principal investigator at each clinical centre (CC) will submit the protocol to the appropriate Local Ethics Committee for approval. The application for approval will include a copy of the participant consents, information sheets and other relevant materials. Approval has already been given by Trent MREC for ProtecT on 21st June 2001.

8.3 Participant Consent (CC)

Persons asked to participate in this research are entitled to choose whether or not to take part. Their decision will be voluntary, and they will be competent to understand what is involved. Consent forms will be designed to assure the protection of their rights.

Participants will receive both written and verbal information. The written information has been approved by the medically qualified investigators. The verbal explanation to the participant will be performed by the research nurse under the supervision of the medically qualified investigators. The verbal explanation will cover all the elements specified in the written information provided for the participant. The participants will be informed of the aims, methods, anticipated benefits and potential hazards of the study including any discomfort it may entail.

The participant will be given every opportunity to clarify any points he does not understand and if necessary ask for more information. At the end of the discussion the participant will be given time to reflect. The participant is at liberty to withdraw their consent to participate at any time, without prejudicing their future medical care.

The research nurse will then obtain the participants' freely given written informed consent for each stage of the study. Both investigators and participants retain copies of the signed consent forms.

8.4 Investigator responsibilities (CC)

The principal clinical investigator at each centre will be responsible for the clinical conduct of the study staff. The clinical investigators will maintain a Trial Master File including a list and CVs of appropriately qualified persons to whom they have delegated significant trial-related duties. The investigator will be responsible that all such identified persons will be thoroughly familiar with the protocol and study procedures, as well as being aware of the principles of good clinical practice (GCP) (MRC Guidelines for Good Clinical Practice in Clinical Trials, MRC 1998). The Lead Nurse shall be appointed by the investigator at each centre and shall have responsibility for the efficient operation of the study to GCP guidelines (SOP Team Management).

© Copyright: The University of Oxford and Oxford University Hospitals NHS Foundation Trust 2016

9. STUDY POPULATION AND PARTICIPANTS

9.1 Participant Enrolment

Recruitment was completed in 2009.
Participants will be recruited through general practices. In each centre, PCTs will be mapped and half the practices randomised to enter the ProtecT study. They will all be men within the age range 50-69 years. All such persons within the practices will be invited to attend for a PSA test to detect prostate cancer. Those men who have confirmed localised prostate cancer will be invited to participate in the treatment trial.

10. INCLUSION AND EXCLUSION CRITERIA

Inclusion criteria

- Age 50-69* years on the date of preparation of the list at the general practice of potential participants
- Male gender
- Able to give informed written consent to participate
- Fit for any of the three treatments and with a life expectancy of 10 years
- Registration with the participating general practice on the date of the PCC (registration with another practice after entry to ProtecT is not an exclusion criteria)

*Invitation of age range 45-49 years pilot was conducted following MREC and LREC approval in one centre. Trent MREC approval 7th May, 2003.

Exclusion criteria
This trial is of pragmatic design. Therefore, exclusion criteria will be kept to the minimum possible. Participants will be excluded from entry if they have:

- Concomitant or past malignancies (other than a small treated skin cancer)
- Prior treatment for prostate malignancy
- Serious cardiac or respiratory problems in the previous 12 months of the PCC, i.e. stroke, MI, heart failure, COPD
- Kidney dialyses or transplantation
- Bilateral hip replacement
- Previous entry to the ProtecT study at a prior general practice

The presence of blood borne infections is not an exclusion criteria.

11. RECRUITMENT OF PARTICIPANTS

Recruitment was completed in 2009

11.1 Recruitment of General Practices (CC and Bristol)

Practices randomised to receive ProtecT will be contacted by the primary care researcher or the UK coordinator for general practices. The GPs and practice manager will be briefed about the ProtecT study, given the protocol and asked for consent for the practice to take part in ProtecT. An information pack will be sent out to each practice randomised to Protect, including details of the ProtecT website. The lead nurse will subsequently visit the practice to establish suitable accommodation for the study clinics and to liaise with practice staff. It is advised that practices are approached initially 3 months in advance of starting clinics. Practices may invoice for costs incurred in preparing the list of eligible men.

© Copyright: The University of Oxford and Oxford University Hospitals NHS Foundation Trust 2016

11.2 Participant Invitation Procedures (CC and Bristol)

There is a Setting up at new General Practices SOP. The clinical secretary will go to the participating GP surgeries and download the name, address, date of birth, NHS number and GP practice identification number of all men aged 50-69 years onto the study laptop computer. If possible, the list of men will have been previously screened by the practice for those unsuitable to participate and noted on the Access database. All individuals invited to participate in the trial will be allocated a unique study number by Bristol. Address labels will be generated and invitations to join the study sent to men in manageable batches (**Downloading protocol for clinical centres**). Letters are mailed out from the general practice on the practice headed notepaper. The data downloaded from the practice computer will only be saved at the practice. As each new practice is visited a record will be made of the doctor's names, address and contact details, computer system and total list size as well as the date of the first invitations on the downloading proforma. This information will be entered on the clinical databases and the proforma sent to the research coordinating centre (Bristol).

The reply slips are returned to Bristol and the names and addresses of men indicating their willingness to join the study will be entered on the project database along with the date on which the reply slip was returned. Men who telephone and indicate either their willingness to participate or refusal are recorded in the same way as for letters.

Those men who do not reply to the initial letter or who decline to participate on the reply slip will have no follow-up within ProtecT and their details will not be recorded on the main database.

Lists of men who are willing to participate are sent electronically via a secure network (NHSnet) to the clinical secretaries every two weeks. Secretaries in clinical centres will arrange appointments for these men and manage the prostate check clinic (PCC) lists, including rearranging appointments where necessary. A participant information sheet outlining the study will accompany the appointment letter (Information Sheet 1). The dates and times of the clinics and attendees will also be entered into the study database. Persons who do not/can not attend their intended appointment will be contacted by telephone and a further appointment organised with details recorded on the database. Should they not attend on 2 occasions it will be assumed that they no longer wish to participate and this will be documented on the study database. The PCC lists of appointments and place of the clinics will also be recorded on the database.

11.3 Participant Visit Schedule (CC)

- All participants: Prostate Check Clinic
- If raised PSA: TRUS and Biopsy
- If PSA PCC is 10-19.99 ng/ml and prostate cancer detected: Bone scan
- If localised prostate cancer: Eligibility appointment
- If eligible for randomisation:
  - Information appointment
  - Clinical follow-up: Active monitoring arm every 3 months in year 1 (see details section 16), other arms clinically determined (NHS care in extended follow-up and thereafter).
  - Post-treatment: Research follow-up annually 12 months after randomisation
  - Extended follow-up: See section 22.9 for details from 2018 to March 2022

© Copyright: The University of Oxford and Oxford University Hospitals NHS Foundation Trust 2016

12. PROSTATE CHECK CLINICS (PCC)

There is a PCC SOP. Recruitment will be performed mostly at the participating general practices, but also at the hospital. On attendance the research nurse (previously carefully instructed by the study team and working from a detailed script utilised in the training programmes) will provide verbal information on the study aims and design. Particular attention will be paid to the treatment phase of the trial. It will be made clear that only those who have localised prostate cancer will be requested to participate in the treatment trial. It will be stressed that the treatment is allocated at random by computer, unless the participant refuses randomisation and selects a treatment regime.

The clinical significance of prostate cancer will be discussed and it will be made clear that participation in the project is purely voluntary and the participant will be free to decline without prejudicing their future care. Participants will be given the opportunity to ask questions. Those who decline to participate will be free to leave and will be thanked for attending. They can have a PSA test if they so wish under current NHS recommendations, preferably with their GP. A letter is posted to their GP.

The research nurse will ensure that all men willing to participate in the trial are eligible to do so (using the eligibility criteria detailed earlier and in the PCC Schedule). Those ineligible to participate will have the reason for ineligibility explained to them and they will be thanked for their attendance and support of the project. They can have a PSA test if they so wish under current NHS recommendations, preferably with their GP. A letter is sent to their GP (Letter Excluded GP).

Men who wish to participate will be asked to give written, witnessed consent (Consent Form 1). Men have a further 24-hour period after the PCC clinic during which time they must sign and return a further consent form (Consent form 3) to agree to the PSA test being processed. A copy of all signed Consent documents will be given to participants.

Men are additionally asked whether they would like to participate in the ProMPT study (Prostate cancer Mechanisms of Progression and Treatment MREC 01/4/061). Men do not have to take part in ProMPT to enter ProtecT. The ProMPT study aims to establish the molecular pathology and mechanisms of tumour progression, develop novel treatment strategies and to evaluate novel markers and treatment approaches. There is an information sheet for the ProMPT study and consent form (Consent form 2), including consent for research on DNA. Trent MREC approved this study on 17 January 2002 and is ongoing to December 31st 2020.

12.1 Initial Data Collection at the Prostate Check Clinic (PCC)

A. Research Nurse

1. A SOP for the PCC details the full data collection procedures and nurse responsibilities.

2. Discusses study information, and requests consent to participate in the ProtecT and ProMPT study (Consents 1 and 2 in Schedule for Prostate Check Clinic).
   - Consent form 1 is for consent to enter the study and take blood for the PSA test and future studies, including checking GP or hospital records
   - Consent form 2 is for consent for the ProMPT research
   - Consents 1 and 2 (one copy) are given to participant
   - A cross is placed in boxes of sections the participant does not consent to, initialled by the participant

3. Completes the S1 Schedule for Prostate Check Clinic containing:
   - baseline socio-demographic data; age, socio-economic status, ethnicity
   - baseline clinical data, e.g. previous urinary problems or PSA tests

© Copyright: The University of Oxford and Oxford University Hospitals NHS Foundation Trust 2016

- exclusion criteria checklist
- a checklist to discuss with participants describing the ProtecT and ProMPT studies

4. Completes a single page version of the data entry form (PCC Proforma) about the attendance at the clinic and outcome. The PCC Proforma is entered onto the study computer at the earliest opportunity by the clinical secretary. Any potential problems with conducting a biopsy, e.g. allergies to penicillin, warfarin etc. are written on the reverse of the sheet as are any other comments regarding the man or the appointment.

5. Checks the participant's case notes if previous PSA tests have been performed and records the results on the form.

6. Records on the PCC Schedule their weight, blood pressure and pulse as well as the study instrument number of the scales and blood pressure monitor. If the blood pressure is above a hypertensive level agreed with the current practice (identified by the lead nurse in initial visits) the participant will be advised to have the blood pressure checked again by the practice nurse and the practice will be informed of the reading.

   If men consent to the PSA test, the nurse takes blood as detailed in the **ProMPT Blood Collection and Storage Protocol.** Laminated sheets of relevant sections of the protocol are available for use in clinics**.**

B. Participants

1. Men complete a study questionnaire (MTQ1) on urinary symptoms (ICS*male*SF *questionnaire*[22] ICIQ questionnaire), general health status (SF-12),[23] Hospital Anxiety and Depression scale[24] (HAD), EuroQol EQ-5D[25] Profile of Moods States and Impact of Events scale[26] which they may return in the post if necessary, using a freepost envelope.
2. Men are given a questionnaire (MTQ1a) with a freepost envelope to complete at home on environmental exposures and prostate cancer for the ProMPT study.
3. 30,000 men over an 18 month period will be given seven day dietary diaries (MTQ1b). The diet diaries will be sent to Bristol for coding and data entry.

12.2 Consent Form 3 ("Cooling Off" Consent) and Notification of PSA Results (CC)

Men have a further 24-hour period after the PCC clinic during which time they must sign and return a further consent form (Consent form 3) to agree to the PSA test being conducted on their blood sample. If men agree, the PSA test is conducted and the results entered onto the project database by the secretary. A photocopy of the consent form is posted to the men and a copy may be held locally with the original returned to Bristol. Participants who telephone are asked to return their consent form by post. Men who do not complete the forms in full are requested to do so by post with the incomplete form posted back (Letter Consent3/retP). If participants do not return this form after being contacted by telephone or letter (Letter Consent3/NRP), or do not consent to PSA testing (Letter Consent3/refP), or do not sign the form, or are ineligible for ProtecT at the PCC, their blood specimens will be destroyed. This information is recorded on the study database. In the case of a non-reply, blood is destroyed one month after the date of the PCC.

All participants will receive the test results by post. The majority (~90%) of participants will have a normal PSA result (i.e. <3.0 ng/ml) and will exit the study (Letter NormP). The participant's GP will be informed of the test results (Letter NormGP).

Consent 3 also seeks further consent for the ProMPT research. Blood for this study is destroyed if Consent 3 is not obtained in the methods as described above.

© Copyright: The University of Oxford and Oxford University Hospitals NHS Foundation Trust 2016

All men with negative PSA results should have their ProtecT study data returned to Bristol for data entry and storage at this stage, i.e. PCC [S] schedule, MT1 questionnaire, Consent 3, grouped inside PCC schedule and recorded on the front of the PCC schedule.

12.3 Eligibility for Diagnostic Phase of the Study

- Men with a raised PSA result ≥3.0 ng/ml[27] and <20 ng/ml from the PCC PSA test
- Men with a raised PSA result of are >19.99 ng/ml are only eligible for a ProtecT biopsy if a reason for the raised PSA at PCC is identified e.g. prostatitis. If they have a convincing diagnosis of prostatitis, the PSA should be repeated before deciding on a biopsy

13. THE DIAGNOSTIC PHASE (CC)

Diagnosis of prostate cancer was completed in 2009

13.1 Diagnosis of Localised Prostate Cancer

All men with a raised PSA result (section 10.3) are invited to attend the Urology department of the clinical centre (Letter ab-lowP). There is a Diagnostic Process SOP. The participant's GP will also be informed (Letter ab-lowGP). The dates of the appointment and the attendance will be recorded on the study database. Locally approved protocols should be utilised for inviting men to biopsy who are on warfarin. The PCC proforma will be reviewed prior to biopsy e.g. for medication and whether to collect additional blood if 44 mls were not obtained at the PCC. Consent for the biopsy will be obtained using local Trust consent forms appropriate for the procedure.

At this appointment they will have a:

- TRUS-guided biopsy (10 cores)[28-30] under antibiotic prophylaxis according to local protocols
- physical examination including digital rectal examination (DRE)
- second PSA test (subsequent action will be taken only on the basis of the PCC PSA test unless a reason for the raised PCC PSA is identified e.g. prostatitis)
- a consent form (Consent 3.1) to request biopsy tissue for the ProMPT study to be used in conjunction with the ProMPT 'patient information sheet for ProtecT patients'

All other tests required to determine eligibility for the ProtecT trial must be completed before the eligibility appointment, preferably within 4 months of the PCC date.

There are several routes through the diagnostic/eligibility phase, depending on PCC PSA level and subsequent test results – each is detailed below (see also Figure 2).

13.2 Criteria for Trial Eligibility when PCC PSA Test is <10ng/ml

All men should proceed to a TRUS-guided biopsy, with DRE and second PSA test.

(a) Men diagnosed with histologically-proven **clinically localised prostate cancer** (T1-T2, NX, M0) (2002 TNM classification),[31] are eligible for the treatment trial (section 14). If **high grade cancer (Gleason score 8-10) is found an isotope bone scan** should be conducted before the eligibility appointment.

(b) Men with any suspicion of **advanced disease** should be investigated fully and if advanced prostate cancer is found, the man is ineligible for the trial and should be treated routinely, but details of the diagnosis to be added to the trial database (see Section 13.5).

© Copyright: The University of Oxford and Oxford University Hospitals NHS Foundation Trust 2016

(c) Men with **negative results** after the first biopsy require the following further tests to determine eligibility:

i. Men with **HGPIN [high grade prostatic intra-epithelial neoplasia]** or **suspicious** findings should be offered a repeat biopsy immediately, as ~50% will have an associated invasive prostatic adenocarcinoma.[32] A letter is sent to the participant's GP and the participant.

ii. Men with **inadequate biopsies** should be offered a repeat biopsy immediately. A letter is sent to the participant's GP and the participant.

(d) Men with a negative **set of biopsies** should have their **free/total PSA ratio** measured [performed in Sheffield on a monthly basis, with samples sent on dry ice, preferably Tuesday–Thursday, with study centre and study no, name, forename, DOB, date of sample**.** Letters are sent to the participant and their GP (Letter neg-biopsy/PSAhighP, Letter neg-biopsy/PSAhighGP).

Further action depends on the result of the free/total PSA:
(i) Men with a **free/total PSA ratio of 0.12 or less** (12% or less) will be offered a **second set of biopsies**.[33,34] If the repeat biopsy indicates localised prostate cancer, they will be eligible for the treatment trial. If the repeat biopsy is negative, the participant should be asked to return to UOP [**Urology out-patients]** for another PSA test, 12 months after the initial measurement at the PCC. If, at this time, PSA has doubled within 12 months, another biopsy should be offered; otherwise, annual PSA tests should be offered at UOP (Letter neg-biopsy/UOP/PSArepeatGP and Letter neg-biopsy/UOP/PSArepeatP).

(ii) Men with a **free/total PSA ratio of >0.12** (greater than 12%) will be offered a **PSA 12 months after the initial test at the PCC**. If, at this time, PSA has doubled within 12 months, another biopsy should be offered; otherwise, annual PSA tests should be suggested conducted by the participants GP. A letter is sent to the participants' GP and the participant (Letter neg-biopsy/GP/PSArepeatGP and Letter neg-biopsy/GP/PSArepeatP). PSA tests can be conducted in secondary care if the urologist prefers (Letter neg-biopsy/UOP/PSArepeatGP and Letter neg-biopsy/UOP/PSArepeatP).

Within the ProtecT study, annual PSA tests should not be offered once the participant is 70 years. Participants should be managed in primary care using standard NHS recommendations.

If, at any time in the diagnostic phase, men are diagnosed with histologically-proven clinically localised prostate cancer (T1-T2, NX, M0) defined according to the 2002 TNM classification, they are eligible for the treatment trial and should proceed to an eligibility appointment (go to section 14).

13.3 Criteria for Trial Eligibility when PCC Or Biopsy PSA is 10-19.99ng/ml

All men should proceed immediately to a **TRUS-guided biopsy**, DRE and second PSA test. The following action should be taken according to outcome:

(a) If biopsy is inadequate, a repeat biopsy should be offered immediately

(b) If first biopsy is **negative** or shows **HGPIN or is suspicious**, a second biopsy should be conducted immediately without free/total PSA measure. If biopsy 2 is negative and PSA remains high or rising in the absence of obvious other reasons, a 3rd and, if necessary 4th, set of biopsies should be undertaken by the most experienced member of the team and targeting the transitional zone, using GA if required (pathology to be informed of targeted biopsies). If all biopsies are negative, PSA should be repeated every 6 months for two years, with further biopsies indicated if doubling time is within 12 months.

© Copyright: The University of Oxford and Oxford University Hospitals NHS Foundation Trust 2016

(c) An isotope bone scan is undertaken if cancer is detected histologically. If the bone scan indicates **skeletal metastases**, the man is ineligible for the trial and should be treated routinely but details of the diagnosis to be added to the trial database (see Section 13.5) (letter advanGP).

(d) If the bone scan is negative and **clinically localised prostate cancer** (T1-T2, NX, M0) is diagnosed, men are eligible for the treatment trial (go to section 14).

If, at any stage, clinically localised prostate cancer (T1-T2, NX, M0) is diagnosed, the man is eligible for the treatment trial.

13.4 Criteria for Trial Eligibility when PCC PSA is >19.99ng/ml

Men with PCC PSA >19.99 ng/ml are likely to have advanced prostate cancer and will be dealt with urgently by the urologist, outside the ProtecT study, with the GP informed (Letter ab-highGP). Those found to have advanced disease are treated according to conventional practice, and are not eligible for the trial. A letter is sent to the participant's GP (Letter AdvanGP). Men with a PCC PSA of <20.0ng/ml and a biopsy PCC of >19.99ng/ml will be eligible for randomisation only if localised cancer is detected and a bone scan was negative. Information on disease grade and staging will be required for the CAP **(Cancer of the Prostate)** and ProMPT studies for all those with cancer who are ineligible for ProtecT and the details of those participants with cancer should be sent to the study coordinator.

13.5 Data Collection at The Diagnostic Phase (CC)

The research nurse completes onto the study computer at the earliest opportunity the clinical stage and grade of the disease, including Gleason scores, and whether other tests have been performed, e.g. bone scans or additional PSA tests. It is possible that there will be several appointments or events and results e.g. bone scans during the diagnostic phase, and data collection must occur at each appointment or event on all men with a raised PSA.

Participants are asked to complete questionnaire (Questionnaire MTQ2) containing similar measures used in the PCC clinic questionnaire (MTQ1) with the addition of the UCLA **EPIC** prostate cancer index.[32 35] The questionnaire will be entered by Bristol**.** The questionnaire should be completed for each biopsy undertaken, including second or third biopsies.

If localised prostate cancer is diagnosed and the participant is excluded for any reason e.g. for health grounds, then this must be fully documented.

All men unable to proceed to the eligibility appointment for whatever reason should have their ProtecT study data returned to Bristol for data entry and storage, i.e. PCC schedule, MTQ1 and MTQ2 questionnaires, Consent 3, Consent 3.1, eligibility proforma grouped inside PCC schedule and recorded on the front of the schedule.

© Copyright: The University of Oxford and Oxford University Hospitals NHS Foundation Trust 2016

Figure 2: The Clinical Diagnostic Process Before Eligibility Is Determined

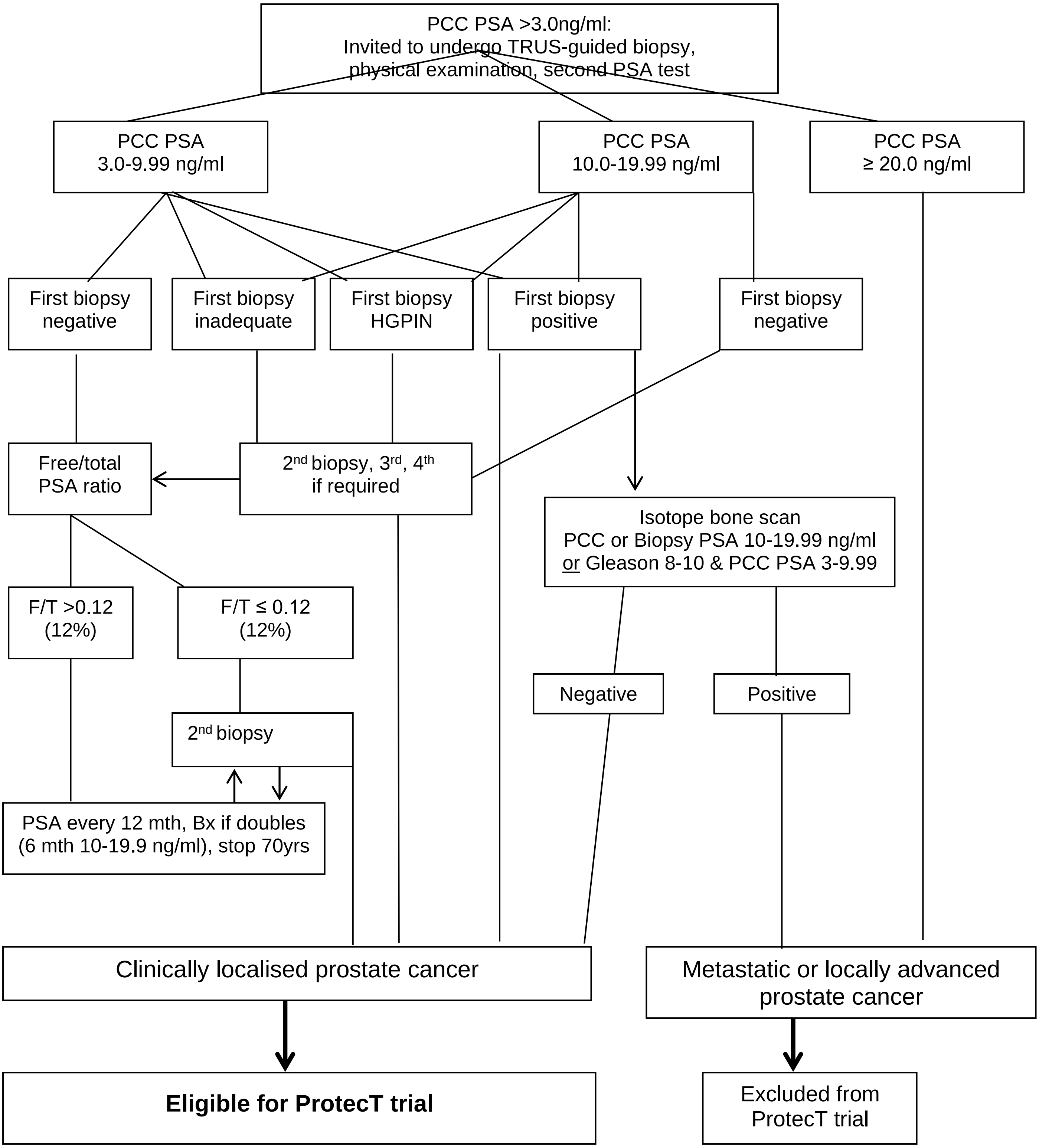

© Copyright: The University of Oxford and Oxford University Hospitals NHS Foundation Trust 2016

13.6 Detection of Localised Cancer at Biopsies Subsequent to Monitoring in Primary Care

Men with negative biopsies at entry to ProtecT who are later shown to have localised prostate cancer at subsequent biopsies, e.g. after a period in primary care, can have an eligibility appointment provided that they fulfil the study criteria for eligibility for randomisation (Section 12.1) and are still within the age criteria for the study.

13.7 Biopsy Classification and Interpretation across Clinical Centres

Quality control will be ensured by regular exchange of material for cross evaluation and confirmation of biopsy interpretations, led by Pathology Management Committee, expert pathologists from the clinical centres**.** There will be an audit of 5% of all TRUS biopsies**.** Data are collected on the biopsy and radical prostatectomy proformas and transferred to the clinical centres database by the research nurses. Urologists are requested to write right then left cores on biopsy forms to pathology. The ProtecT pathology classification scheme is:

**B1**	**Inadequate specimen (no prostate tissue identified)**	
**B2**	**Benign**	
**B3**	**Epithelial atypia not amounting to HGPIN**	
**B4**	**(tick either or both boxes as applicable)**	**Subcategory B4a: HGPIN**<br>**Subcategory B4b: Atypical small acinar proliferation (ASAP)**
**B5**	**Adenocarcinoma**	

14. ELIGIBILITY APPOINTMENT FOR PARTICIPANTS WITH CONFIRMED LOCALISED PROSTATE CANCER (CC)

Men with confirmed localised prostate cancer are invited to attend an eligibility appointment **with the study urologist** (Letter DiagP and to their GP, Letter DiagGP). **All diagnostic tests and staging must be completed before the eligibility appointment.** This is a relatively short appointment in which the urologist explains the diagnosis, gives the participant an information sheet (Information Sheet 2) and invites the participant to attend a longer 'information' appointment with the research nurse (or the urologist if the participant requests) up to and no longer than 10 days later (unless the participant requests a delay). The feasibility study showed that this two-stage process is effective and efficient: men are often shocked by the diagnosis and need time to reflect on this and the advantages and disadvantages of the treatments before the trial randomisation ('information') appointment. Centres may also provide local audited figures for the side effects of treatments in addition to those provided in Information sheet 2. There is a Diagnostic Phase Summary Report that can be checked at this appt.

© Copyright: The University of Oxford and Oxford University Hospitals NHS Foundation Trust 2016

14.1 Eligibility Criteria for Randomisation (CC)

The urologist must ensure that all participants for randomisation fulfil these criteria:

1. Clinically localised prostate cancer (T1-T2, NX, M0) defined according to the 2002 TNM classification[31]
2. PSA at PCC in the range 3.0-19.99 ng/ml
3. No metastases from isotope bone scan (PCC PSA was 10-19.99 ng/ml or Gleason grade 8-10)
4. Fit for all three treatments (do not exclude on basis of age or cancer volume)

If the volume of cancer detected on biopsy is very small but the diagnosis is unequivocal, the case should be scrutinised with input from the pathologist but these patients are eligible for randomisation, and should NOT be excluded on the basis of tumour volume. The PIs are always happy to discuss these cases with the Urologist concerned as necessary.

14.2 Data Collection at The Eligibility Appointment (CC)

The urologist completes the eligibility schedule (S2 Schedule for Eligibility appointment), which records the clinical stage of the disease, whether the man is fit for all three treatment options and a checklist of issues discussed with the participants. Even if the participant has a strong preference for a treatment an information appointment should be arranged. If the information appointment does not happen, an information schedule is completed recording his selected treatment.

The whole consultation is recorded on tape unless the participant declines to allow the taping. There is a Recording SOP including digital tapes. At the end of the appointment, the tape is rewound and marked with the participants study number and placed in the locked cabinet identified for the study at the clinical centre. Audio tapes are sent to Bristol with the questionnaires and schedules on a regular basis and digital tapes by the NHS net. The tapes are booked in, transcribed and stored in a locked filing cabinet in Bristol.

15. INFORMATION APPOINTMENT (CC)

The main purpose of this information appointment is to provide the participant with sufficient information to allow him to decide whether or not he is willing to be randomised to the randomised controlled trial of active monitoring versus radical prostatectomy versus radical radiotherapy (3 arms).

The nurse-researcher emphasises the need for a trial of treatments, describes the advantages and disadvantages of each of the three treatments and explains the purposes of randomisation. The information content and delivery has been determined by the feasibility study and the nurse will work to a detailed but flexible script. Men should not undergo randomisation unless they are able to view all treatments as reasonably equivalent and at that stage randomisation should then proceed. There is an Information Appointment SOP.

© Copyright: The University of Oxford and Oxford University Hospitals NHS Foundation Trust 2016

16. RANDOMISATION (CC AND BRISTOL)

Randomisation was completed in 2009.

The participants give their written consent to be randomised (Consent form 4) and a copy of the form is given or posted to the participant (Consent 4 is not the acceptance of treatment allocated from the randomisation). Once the participant has given written consent to be randomised, the nurse will telephone Bristol for the treatment allocation which will be performed in Bristol. The participant is told the allocation and asked whether he accepts it. He may want time to think or to talk to other clinicians.

16.1 Minimisation Variables

- Participants' age on the date of the general practice list being made (and DOB for confirmation) (stratified into four 5 year age bands)
- Gleason score (stratified 2-4, 5-7 or 8-10)
- Average result of PCC and 1st Biopsy PSA tests (stratified <6, 6-9.9 or ≥10 ng/ml)

These variables and the man's study number are emailed to the nominated Bristol secretary when the eligibility and information appointments are arranged with the date of the appointment (copied to the trial coordinator).

If a participant had negative biopsies then re-entered the study after a period of PSA monitoring in primary or secondary care, followed by a positive biopsy, then the 2 latest PSA tests will be used for minimisation purposes.

If a participant is aged 70 years by the time of the PCC they will be entered as 69 years for minimisation purposes only.

If the Gleason sum score is missing it will be entered as 6 for minimisation purposes only.

16.2 The Main Study: the Three-Arm Trial

The primary intention in the information appointment is to recruit informed participants to the main three-arm trial comparing active monitoring, radical prostatectomy and radical radiotherapy.

16.3 Alternative to Randomisation

If randomisation is unacceptable to the participant, a participant-led selection of a treatment option will be reached without randomisation. These participants will be the 'selection group', including (rarely) if the participant does not have an information appointment.

16.4 Data Collection at Information Appointment (CC)

The nurse will complete a Schedule for the Information Appointment (S3) which records a checklist of issues discussed with participants, and the decision reached regarding randomisation and the allocation for the participant. If the participant refuses to be randomised this is recorded. If further appointments are required, then the new appointment date is also recorded on the Schedule and the schedule is completed for each appointment.

© Copyright: The University of Oxford and Oxford University Hospitals NHS Foundation Trust 2016

The whole consultation is recorded on tape unless the participant declines to allow the taping. At the end of the appointment, the tape is marked with the participants' study number and placed in the locked cabinet identified for the study at the clinical centre. These tapes are sent to Bristol, as described in 14.2.

16.5 Data Collection after Treatment Allocation

All men are asked to be followed up, whichever treatment decision is reached, including selection participants. A letter is sent to the participant's GP indicating his treatment allocation (LetterRCTGP) or treatment selection (Letter PrefGP). Bristol are informed of the participant's treatment (study secretary and trial coordinator). The date of the treatment allocation acceptance is recorded in the Information Schedule (S3) and the centres database.

All men who have been randomised or have selected a treatment should have their ProtecT study data returned to Bristol for data entry and storage, i.e. PCC schedule, MTQ1 and 2 questionnaires, Consent 3.1, Eligibility and Information schedules, recorded on the front of the PCC schedule.

17. TREATMENT (INTERVENTION) PROTOCOLS (CC)

The treatment phase of the trial is completed, and all participants are under the clinical care of their local NHS team. **These treatments are to be given to participants either randomised to or selecting a particular treatment.**

All participants will receive a detailed study patient information booklet regarding the risks and benefits of the treatment regime to which they have been allocated or have selected, and the processes involved, including clinical follow-up. Locally produced information booklets may also be given to participants at this stage. Participants not indicating their choice of treatment or acceptance of allocation (by 6 months after randomisation) will be deemed to be on active monitoring until the participant indicates otherwise.

18. ACTIVE MONITORING

Men undergoing active monitoring will return for an appointment three months after randomisation to refine their plan of management. This appointment will usually be conducted by the research nurse and only staff connected with the study should undertake these appointments. PSA results, any additional tests or review appointments will be recorded on the Active Monitoring Treatment schedule (S4rAM) at each appointment. There will be an annual check of participant notes by the lead urologist to sign off the annual follow-up.

18.1 Treatment Details and Follow-Up Pathway

Their individual plan of management will be decided jointly by the participant and urologist or research nurse, but will include:

- PSA every three months in year 1, then 6 months thereafter
- Opportunity for a digital rectal examination (DRE) at the annual review appointment conducted by urologist as indicated (rise in PSA, new symptoms etc.)
- Opportunity for a review appointment (Section 16.2)

© Copyright: The University of Oxford and Oxford University Hospitals NHS Foundation Trust 2016

Prior to each follow-up visit, a PSA test will have been performed and results obtained so that at each visit, PSA results will be plotted and examined for any evidence of a rise that might indicate disease progression. Other factors that may cause increased levels, e.g. infection will also be investigated. The aim of active monitoring is to detect disease progression as early as possible, preferably while the tumour is still localised, but also to allow those whose disease remains stable to avoid intervention. The active monitoring pathway is shown in Figure 3.

18.2 Assessing PSA Changes over Time

Prior to each visit, the PSA test will be conducted so that the result can be plotted in the Active Monitoring Schedule. At each visit, the research nurse (or urologist) will assess the PSA results over the preceding 12 month period. If there is a rise in PSA level of 50% or more in that 12 month period, the participant will be asked to return for a repeat PSA test a minimum of six weeks and up to nine weeks later. Action is taken if the repeat PSA test confirms the 50% or greater rise over the original 12 months period, the participant will have a review appointment with the study urologist to discuss the implications of the rise and current options (see below). If the repeat PSA test does not confirm the previous 50% rise, the participant will return to regular Active Monitoring appointments. If at any time the nurse or participant is concerned about the PSA level, they may request a review appointment (see below).

If a participant is concurrently prescribed the 5-alpha reductase inhibitor finasteride then the measured PSA value is doubled (measured is 6 ng/ml is taken as 12 ng/ml) and action taken on the doubled value.

18.3 Review Appointment and Annual Bone Scans

A review appointment should be arranged with the study urologist in the following circumstances:
1. The PSA level has been assessed as defined above as 'rising'
2. If any symptoms of spreading disease (urinary or systemic) become apparent
3. If the participant or nurse is anxious about the PSA level or other concerns

At the review appointment, the study urologist will discuss the issues raised and current options, including remaining on Active Monitoring, undergoing re-staging of the cancer, or having other treatments, as appropriate. Other treatments might include radical prostatectomy or radiotherapy if the cancer still appears to be contained within the prostate. If the cancer is not contained within the prostate, treatment options would include transurethral resection of the prostate for bladder outflow obstruction, hormone treatment, radiotherapy and other relevant treatments.

NB: Bone scans are conducted annually on participants once the PSA reaches at least 10 ng/ml. If the PSA remains at above this level the following year another bone scan is performed even if there has been no change in the PSA level.

© Copyright: The University of Oxford and Oxford University Hospitals NHS Foundation Trust 2016

Figure 3: Active Monitoring Follow-Up Pathway

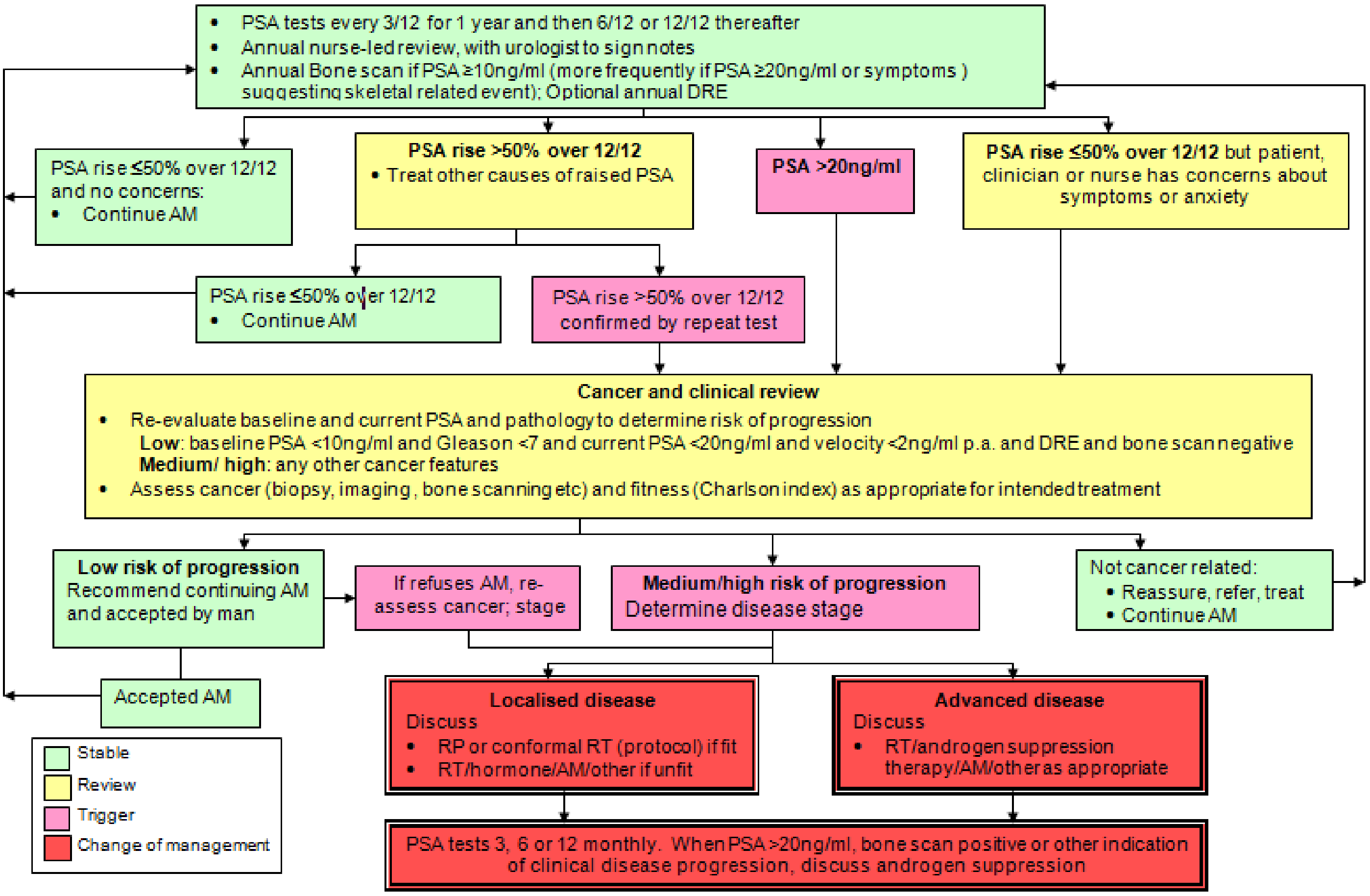

19. RADICAL PROSTATECTOMY

Participants undergoing radical prostatectomy will be listed for surgery optimally within 2 weeks, and no longer than 3 months, unless specifically requested by the participant for personal reasons.

Accurate per-operative data will be collected, including details of any complications. Standards of performance by the surgeons will be reviewed continually by the steering group.

19.1 Treatment Details

Participants will have the procedure explained in detail, as well as possible morbidity and complications. Participants will be admitted 24 hours prior to the surgery, which will only be performed by the surgeons involved in the trial. The steering group will ensure that these surgeons have a high level of expertise in performing the procedure by auditing retrospectively the results of their last 20 cases by the appropriate technique prior to involving them in the study. Alternatively, surgeons may elect to visit a centre of excellence in either Europe or USA to receive training. The TSC will be provided with anonymised surgeons' data. If any surgeons are outside acceptable limits, the TSC Chairman will be informed and will discuss the implications and necessary action with the PIs.

© Copyright: The University of Oxford and Oxford University Hospitals NHS Foundation Trust 2016

Pelvic lymphadenectomy and radical prostatectomy will be performed following the conventional anatomical retropubic approach as described by Walsh.[36] Laparoscopic and robotic prostatectomy approaches are also possible. The decision to undertake a nerve-sparing operation will be at the discretion of individual surgeons, depending on individual cases and after discussion with the participants. Prospective collection of data, outcome, rates of complications and results will be used for continuous monitoring of the quality of surgery performed. This is to ensure that the other treatment options are compared with the best possible surgical outcome. During surgery, participants will have their node status assessed:

a) Those with a PSA less than 10 ng/ml and a Gleason score <8 will undergo pelvic lymphadenectomy and radical prostatectomy.

b) Those with a PSA of 10 ng/ml or more and/or a Gleason score of 8 or over will undergo a frozen section biopsy of the pelvic lymph nodes prior to prostatectomy. If the lymph nodes are positive, the participant's further management will be at the surgeon's discretion.

19.2 Data Collection

Accurate operative details will be recorded using the S4 Radical Prostatectomy Schedules (Surgeon) and (Researcher), including: length of the operation, blood loss, technical difficulties, unilateral or bilateral neurovascular bundle preservation, intra-and per-operative complications, the presence of urinary leaks or bleeding post-operatively, length of hospital stay and occurrence of any immediate post-operative complications, as well as general recovery from the surgery.

The participant will be discharged home and re-admitted 1-2 weeks later for trial without catheter. Continence will be assessed accurately at this stage, and if satisfactory, the participant will be allowed home, for further clinical follow-up at six weeks (histology) and 3 monthly.

19.3 Histopathological Staging and Evaluation

This will be performed in a unified manner, with collaboration between respective histopathologists in the centres involved, using conventional tissue handling and histopathological criteria. Quality control will be ensured by regular exchange of material for cross evaluation, led by the lead pathologist**.**

19.4 Positive Surgical Margins

This will be defined following conventional histopathological criteria. The association with capsular invasion and seminal vesicle involvement will be documented carefully. Adjuvant treatment (radiotherapy *and/or* hormonal manipulation) for these participants will be at the discretion of individual surgeons after discussion with the participant, and will be guided by PSA levels after the surgery.

19.5 Follow-Up Pathway

The follow-up pathway is shown in Figure 4.

© Copyright: The University of Oxford and Oxford University Hospitals NHS Foundation Trust 2016

Figure 4: Radical Prostatectomy Follow-up Pathway

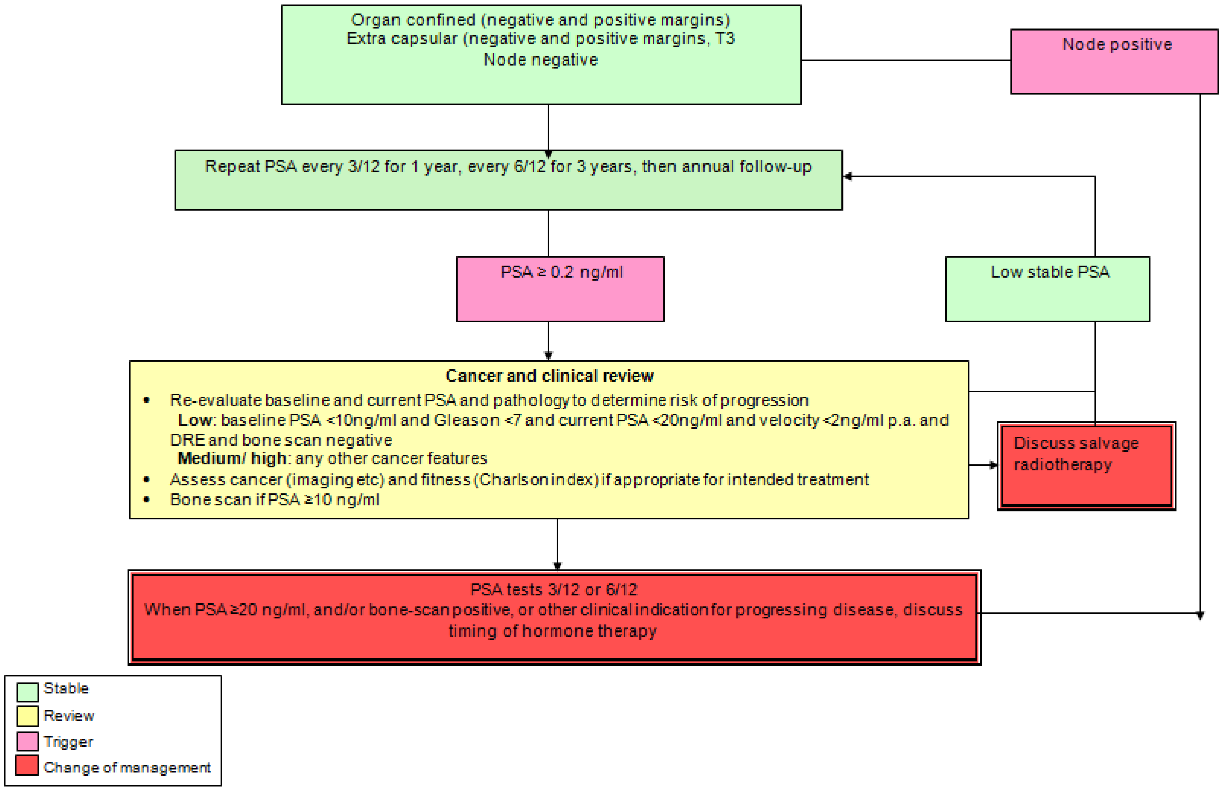

20. RADICAL CONFORMAL EXTERNAL BEAM RADIOTHERAPY V3.0

The radiotherapy protocol has been written by M Mason, P Kirkbride, FC Hamdy, R Moore. Participants undergoing radiotherapy will receive 3-D conformal external beam radiotherapy. Radiotherapists with a special interest in uro-oncology will be responsible for this treatment, and their results audited retrospectively before and prospectively throughout the trial.

This treatment protocol is to be given to participants either randomised to or selecting a particular treatment.

20.1 Treatment Details

All participants will receive neoadjuvant hormone therapy for 3-6 months followed by external beam radiotherapy using 3D conformal methods. Participants will not be eligible to receive prostate brachytherapy, either as sole treatment or as a boost following external beam treatment.

Neo-adjuvant Androgen Deprivation will be achieved in all patients using LHRH agonists at four weekly cycles in conjunction with initial Cyproterone Acetate (CPA) or equivalent alternative, to prevent "flare phenomenon". The CPA or equivalent should commence approximately one week prior to the first LHRH agonist injection and should be given for a total of at least three weeks. The duration of Androgen Deprivation

© Copyright: The University of Oxford and Oxford University Hospitals NHS Foundation Trust 2016

should be at least three months and a maximum of six months, prior to commencement of radiotherapy and should continue until the end of radiotherapy.

20.2 CT Planning Requirements for Radiotherapy

CT planning scan should be done about 4 weeks before the commencement of radiotherapy. Participants will be treated in the supine position. The bladder will be moderately full, (participant to drink about 500 mls 1 hr pre-scan) and the participant should be asked to empty the rectum as free of faeces and flatus as possible. No oral, rectal or intravenous contrast should be used. Positioning/immobilisation will be using current departmental methods. Reproducibility of the positioning of the participants will be maintained using orthogonal laser beams or an equivalent method. The clinical and planning target volumes will be defined on CT scans, which will be taken at no more than 5 mm intervals (5 mm slice thickness). Scans will be taken from the bottom of the sacro-iliac joints to the penile urethra (usually 1 cm below ischial tuberosities will be adequate).

20.3 Volumes and Dose Reference Point

Clinical target volume (CTV) will be outlined by clinicians or authorised planning staff on CT scans taken in the treatment position as above. Outlining should be done on at least 12 (not necessarily contiguous) CT slices, so that the beam portal may accurately conform to the shape of the prostate, plus or minus seminal vesicles. The clinical target volume (CTV) can be accurately defined on CT images, however the planning target volume (PTV) is more difficult to define accurately and computer generated region growing algorithms are recommended to define the required margins. Volumes will be defined according to ICRU Report 50.[37]

Two groups of participants will be defined:

- **Group L** (low risk of seminal vesicle involvement)

  clinical stages T1b/c or T2a with (PSA + [(Gleason score –6) x 10]) <15
- **Group M** (moderate or high risk of seminal vesicle involvement)

  clinical stages T1b/c or T2a with (PSA + [(Gleason score –6) x 10]) $\geq$15

  or patients with clinical stage T2b

CTV will be defined on the basis of clinical and radiological staging as 1) either prostate and base of seminal vesicles or 2) to include prostate and all of the seminal vesicles. No deliberate attempt will be made to include lymph nodes, as adjuvant lymph node irradiation has not been shown to be beneficial.

	Phase I	Phase II
Group L<br>CTV	Prostate & base SV* (CTV_1)<br>No margin beyond organ	Prostate only (CTV_2)<br>No margin beyond organ
PTV	CTV_1 + 10mm	CTV_2 + 5 mm
Group M<br>CTV	Prostate & SV (CTV_1)<br>No margin beyond organ	Prostate only (CTV_2)<br>No margin beyond organ
PTV	CTV_1 + 10mm	CTV_2 + 5 mm

© Copyright: The University of Oxford and Oxford University Hospitals NHS Foundation Trust 2016

*base of SV defined as 5mm margin, radially extended from the prostate defined.

20.4 Organs at Risk

Normal tissues may be outlined by authorised planning staff and will include bladder, rectum and femoral heads together with the body contour. The normal tissues will be outlined and considered as solid organs. Bladder should be outlined from base to dome. The rectum should be outlined from the anus taken at the level of the ischial tuberosities **or** 1 cm below the lower margin of the PTV, whichever is more inferior, to the recto sigmoid junction. This will give length of approximately 12 cm in most cases. Any additional bowel in the treated volume should be outlined separately.

20.5 Simulation Procedures

All treatment isocentres should normally be simulated for phases 1 and 2. After simulation the shape of the multileaf collimator (MLC) leaves or cerrobend blocks should be indicated on simulator films or digitally reconstructed radiographs (DRR). In the simulator, the position of the isocentre should be determined using orthogonal anterior and lateral fields.

20.6 Treatment Technique

The field arrangement for both phases will use a 3-field or a 4-field technique. A 4-field technique should only be used if the MLC movement plane is not in the same plane as the wedge. 3-field techniques should use anterior and left and right lateral fields (which may be modified with slight obliquity to left and right posterior oblique on an individual patient basis). 4-field techniques should use anterior/posterior and right and left lateral fields. 6-field techniques are not required.

If seminal vesicles are wrapped around rectum, then clinical advice should be taken from the consultant directing the treatment. Factors reported of consequence include patient age (younger patients may have increased risk of SV involvement), diabetes and more strongly, haemorrhoids (which may increase risk of rectal damage). Enclosing the rectum with >60% dose may increase rectal damage, and this is more likely in patients with a small rectal area in the transverse section. See recommendations in 18.8 Dose Specification.
The use of multisegment or IMRT solutions should be discussed with the trial QA group.

20.7 Dose Computation

Three dimensional dose distributions should be produced. Beam's eye view representations of PTV and organs at risk will be reproduced for each treatment beam and additionally in the mid-axial plane. If there is marked variation in participant contour further axial distributions should be obtained 2 cm from the cranial and caudal field edges. Ideally, a mid-plane sagital dose distribution should be produced.

Computer data representing dose distributions, CT images and contours should be archived. (Also see paragraph 18.10.)

20.8 Dose Specification

Dose prescription to participants will be 74 Gy in 2 Gy fractions; the phase 1 dose will be 56Gy in 28 fractions, and the dose to phase 2 will be 18Gy in 9 fractions. All doses are to be defined at the isocentre. All fields will be treated daily on a linear accelerator of 5 MV or greater. The planned overall treatment time will be 7.4 weeks. Phases 1 and 2 shall use shaped field throughout, with permission for up to total 5 (five) treatment rest days at any time during treatment. The rationale is that a) more departments have matched MLC linacs facilitating patient transfer b) use of open fields for phase 1 includes more rectum than use of open field for phase 2 (at same dose per fraction to isocentre). If more than 5 days gap is likely to occur, use of standard blocks positioned to approximate conformal shielding should be (virtual) simulated then treatment verified on treatment unit.

© Copyright: The University of Oxford and Oxford University Hospitals NHS Foundation Trust 2016

Minimum and maximum (area of at least 2 cm^2) dose within the defined PTV would normally be 95% and 105% respectively. A hot spot dose outside the PTV will not exceed 105%.

Dose to organs at risk outside the PTV will not exceed the prescribed dose to the isocentre.

Dose volume histograms evaluating dose to CTV, PTV and organs at risk (rectum, femoral heads and bladder) shall be used to ensure the following dose constraints:

For bladder:

- <25% volume to receive dose >74Gy, i.e. 100%
- <50% volume to receive dose >67Gy, i.e. 90%

For rectum:

- Up to 3% of rectum permitted to receive >= 74Gy i.e. 100% (3% represents rectal volume within PTV)
- <25% of rectum permitted to receive >= 70Gy i.e. 95%
- <30% of rectum permitted to receive >= 67Gy i.e. 90%
- <50% of rectum permitted to receive >= 55.5Gy i.e. 75%
- Remainder of rectum permitted to receive <= 44Gy i.e. 60%

Using sagittal reconstruction it is recommended that 60% isodose should not cross posterior rectal contour.

Cumulative dose to the femoral heads should not exceed a maximum dose of 55 Gy to a volume of $\geq$ 2 cm^3. Dose corrections will be made for the femoral heads either on a pixel by pixel basis or using a standardised value of bone density. Departmental procedures concerning rectal gas shall be followed.

20.9 Treatment Verification

Orthogonal portal images or check films will be taken during treatment during phase I (3 or 4 field). When portal imaging devices are available daily images will be taken during week 1 and thence at weekly intervals. When using film at least 2 sets of images will be taken during the first week of treatment.

Port films will be compared to simulator images (or DRR). Treatment accuracy to within 2-3 mm is to be obtained whenever possible and positioning errors of 5 mm and greater are unacceptable. Corrections of participant positioning and appropriate resimulation will be employed if systematic errors greater than this magnitude are apparent. The departmental protocol shall include a specific number of observations on which resimulation is undertaken. (For example 3 observations of a discrepancy >=5 mm).

20.10 Quality Assurance and Data Collection

Participants will be required to follow trial QA protocols as issued.

A questionnaire, planning consistency evaluation and dosimetry checks will form part of the quality assurance. Process documents will be produced by each participating centre, and a radiographer's log detailing verification data will be collected, using the format of the MRC RT01 study.

Data from the first 5 patients randomised since January 2003 must be printed on 'hard copy' and, additionally, in electronic format. Also, every subsequent 7th patient will be hardcopied. All trial patient plans should be archived and made available electronically.

© Copyright: The University of Oxford and Oxford University Hospitals NHS Foundation Trust 2016

Computer data representing dose distributions, CT images and contours should be archived. The data shall be exported in either native file format, DICOM-RT or RTOG format. They will be collected during one of the QA visits or via alternative systems (e.g. ISO 9660 CDROM or DAT UNIX tar, bru, compress, gzip). The data will be pseudo-anonymised when centrally stored. Data transfer and storage policy will follow the trial guidelines on data protection.

QA data will include:

1. Hardcopy and data representation of all outlining, target volume and critical organ definition.

2. Hardcopy and data representation of treatment dose distribution plans and dose volume histogram for all outlines defined.

3. Simulator images: copy films or scanned films or electronic images.

4. Verification images: copy films or scanned films or electronic images.

The data above will be submitted to the QA Physicist on a minimum six monthly basis or at prearranged collection visits.

20.11 Follow-up Pathway

Participants will be seen one month after completion of treatment, and thereafter 3-monthly for the first year, 6-monthly for the second year and then annually thereafter until disease progression (see below).

© Copyright: The University of Oxford and Oxford University Hospitals NHS Foundation Trust 2016

20.12 Disease Progression

The follow-up pathway is shown in Figure 5.

Figure 5: Radiotherapy Follow-Up Pathway

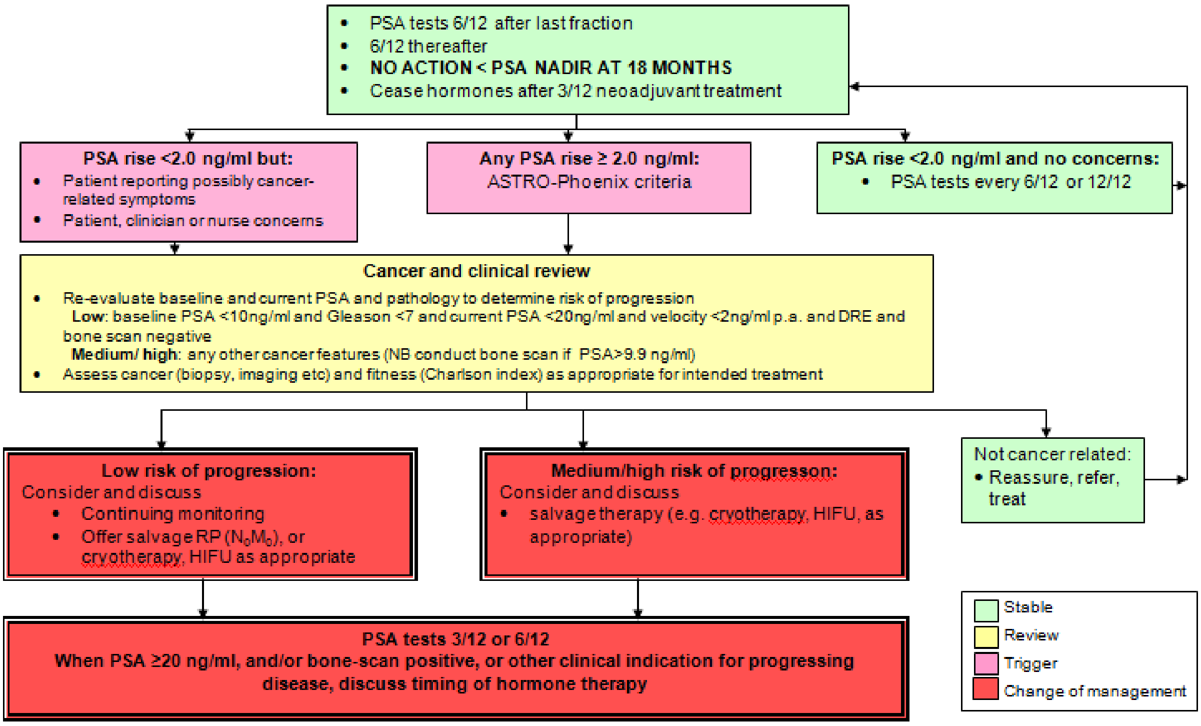

21. RECRUITMENT FLOW

Recruitment completed in 2009.

Each centre can recruit approximately 240 PCC attendees per month when the centre is fully established. 22 participants each month will have a raised PSA (11%) and will require a biopsy. 4 localised cases of prostate cancer will be identified per month (48 per year) and will be eligible for randomisation and follow-up.

22. RESEARCH DATA COLLECTION (CC AND BRISTOL)

See section 22.9 for details of extended follow-up from 2018 toMarch 2022.

All participants diagnosed with localised prostate cancer will undergo research data collection (comprehensive cohort principle). This includes those who were randomised, selected a treatment without randomisation, as well as those who sought therapies not offered by ProtecT, e.g. brachytherapy, or who are treated in private practice by any modality.

22.1 Evaluation of Detection (CC and Bristol)

Recruitment and cancer detection completed in 2009.

Records will be kept of the response rates and the accuracy of the tests at each stage of the study. The positive predictive value of the PSA test will be calculated using histological confirmation as the 'gold standard'. The numbers and specific tests required by men with initially abnormal PSA levels (e.g. confirmatory PSA, TRUS

© Copyright: The University of Oxford and Oxford University Hospitals NHS Foundation Trust 2016

with or without biopsy, bone scan) will be carefully documented to evaluate the urological workload**,** including those ineligible for randomisation.

22.2 Adverse Events (CC)

There will be no adverse event reporting in extended follow-up from 2018 to March 2022.
An adverse event (AE) is defined as any untoward medical occurrence in a participant which does not necessarily have a causal relationship with this treatment.

Adverse events including treatment complications resulting from any of the three treatment arms will be recorded by the nurse on the diagnoses data entry form in the clinical centres database with the date of onset and resolution. Serious AE are defined by the MRC GCP guidelines:

Serious adverse events includes any **untoward** medical occurrence that:

- results in death
- is life-threatening
- requires in-patient hospitalisation or prolongation of existing hospitalisation
- results in persistent or significant disability or incapacity

All serious adverse events (SAE) should be notified within 48 hours of occurrence to the Chief Investigator and the study co-ordinator. Death notification should occur within 24 hours of the study team learning of the event. SAE probably related to participation in the trial are reported to the MREC within 15 days on the NRES SAE proforma. There may be a requirement from the local R&D office to report SAE, and this should be established by a centre.

22.3 Pathology Findings (CC)

Diagnosis completed in 2009.
All biopsy results of ProtecT participants will have a pathology biopsy proforma completed by the pathologist and data entered on the clinical centres database by the research nurses. The radical prostatectomy pathology proforma will also be completed and entered in the same way.

22.4 Follow-Up Timescale and Participant Group

See section 22.9 for details of extended follow-up from 2018 to March 2022.

Research follow-up will be timed from the final information appointment (i.e. date randomised). If there was no information appointment, the eligibility appointment is utilised. All localised cases of prostate cancer will undergo research follow-up, unless the participant withdraws completely from the study. Participants in whom disease progression to T3 or T4 occurs after randomisation will continue to have research follow-up unless the participant withdraws. There is a follow-up SOP covering Treatment and Research follow-up. Participants changing prostate cancer treatments should have the appropriate treatment schedule completed for the additional treatment. Efforts should be made not to "double count" events and to adhere to the annual timings of the follow-up.

22.5 Research Data Collection of Treatments

See section 22.9 for details of extended follow-up from 2018 to March 2022.

Data collection in each treatment arm will be timed around the commencement of the treatment regime. There are data collection schedules for each of the three treatment arms (S4 Schedules) collecting information on the procedures, complications and resource use. These schedules comprise:

© Copyright: The University of Oxford and Oxford University Hospitals NHS Foundation Trust 2016

Active monitoring: S4RAM sheet and chart completed at each visit

Surgery:

- S4Surgery surgeon completed on the date of surgery by the surgeon
- S4Surgery researcher, completed by the research nurse pre-and post-operatively and by the surgeon at 6 weeks post-surgery

Radiotherapy: S4RT Radiotherapy completed pre-radiotherapy by the research nurse, during treatment by the radiotherapy staff and at 6 weeks post-radiotherapy by the oncologist, including a radiographers log, completed by the radiographer during radiotherapy

Any biopsies conducted in follow-up should have a biopsy proforma completed by the pathologist and entered on the centres database.

22.6 Participant Data Collection at Six, 12 Months and Annually

See section 22.9 for details of extended follow-up from 2018 to March 2022.

Research data collection for the participants will commence 6 months after the date of the first information appointment, involving a postal questionnaire on resource use as well as the instruments used at baseline on anxiety and depression, urinary symptoms, sexual function and treatment related quality of life. Questionnaires (MTQ4) will be posted to participants after any changes of address or contact status are reviewed on the database by the clinical centres. A reminder is sent out 4 weeks after non-return of the questionnaire, including a small study token e.g. a ProtecT ballpoint pen. If there is no response and the details are checked and are correct at the clinical centre, the participant will be telephoned by the research or clinical centre 6 weeks after the original mailing. If no telephone contact is possible a short version of the quest (MTQ4a, MTQ5a and MTQ6a) will be posted recorded delivery. Methods to complete the questionnaire using a secure website will be developed, especially for those participants who have moved from the study centre. Participants potentially lost to follow-up will be traced using the NHS tracing service. The revised questionnaire also requests details of a second contact person for participants and these individuals will be contacted if other methods are unsuccessful. This person will also be contacted should the participant lose capacity to contribute to the study to ascertain the participants wishes regarding the study. There is a SOP Follow-up of men following treatment decision: Part III Questionnaire follow-up.

At 12 months and annually thereafter, participants will complete a version of the study questionnaire (MTQ5 and MTQ6), similar to that completed at six months.

22.7 Research and Other Data Collection at 12 Months and Annually (CC)

See section 22.9 for details of extended follow-up from 2018 to March 2022

Full research data collection will take place at 12 months, and thereafter annually, after the date of the first information appointment.

Participants will be seen by one of the study nurses who will complete the Researcher 12 month (S5) or Annual Data Collection schedule (S6) based on the notes from the database and hospital notes as well as the participant interview. Research blood will be collected for urological research in those participants who have consented to this aspect of the study.

Men who move away should have a letter sent from the investigator to the local consultant detailing the study and enclosing the protocol. If the consultant is willing to send protocol based-PSA measurements to the clinical centre that would be continuing study follow-up. Research data collection should be obtained by telephone or by visiting the nearest study centre if feasible. The nurses will also request the details of a

© Copyright: The University of Oxford and Oxford University Hospitals NHS Foundation Trust 2016

relative or partner who could act as a second point of contact should the participant be un-contactable by the usual means.

22.8 Survival Data

All participants who consented to flagging with national registries have been flagged with NHS Digital.

All men participating in the study who give their consent to record flagging (Consent 1 in the S1 PCC Schedule) will be flagged at the UK National Health Services Digital to ensure that the primary outcome of the study, time ascertained at 10 and 15 years, can be analysed. Notification of mortality and cancer incidence amongst study participants will be achieved through flagging and through links with hospital pathology and clinical services in the ProtecT catchments and participating GP practices. Clinical centre staff will return to the general practices to obtain the NHS number of those men who attended the PCC to allow automated flagging. Notifications from NHS Digital will be entered on the study database.

22.9 Extended Research Follow-up from 2018 to March 2022 and COVID-19 amendments

Participants diagnosed with prostate cancer will be followed up in four ways:

1. Passive follow-up by notification of vital status from national registries.

Notification from NHS Digital several times annually to central research team for participants who consented to central registry flagging. Vignettes created for cause of death committee to ascertain the underlying cause of death using medical record review at centres by trial staff from the research centre and OU. Death certificate data will be used to derive overall survival.

COVID-19 issues

University of Bristol (UoB, the research centre) closed their buildings (24th March 2020) preventing access to NHS Digital routine data due to the System Level Security Policy. Trial staff stopped note reviews at centres (16th March 2020, field work stopped by UoB).

Amendment

Cause of death committee suspended (24th March 2020).

2. Passive follow-up for clinical outcomes.

NHS routine data received several times annually by central research team. Outcomes will be derived from routine data by the central research team. Medical record reviews at centres will be targeted to supplement and validate routine data and will be conducted by research centre staff (UOB and UO) (and, if locally available, research staff at centres).

COVID-19 issues

UoB closed their buildings (24th March 2020) preventing access to NHS Digital routine data. Trial staff stopped note reviews at centres (16th March 2020, field work stopped by UoB).

Amendment

Urologist Principal Investigators to conduct medical record reviews by remote access to Trust Electronic Health Records (EHR). The CI confirmed with 8 of the 9 centre PIs that they had remote access and wished to help.

3. Participant questionnaires for patient-reported outcomes

A shorter questionnaire than previously is posted to participants annually from the research centre (MTQ13) up to and including 15 years of individual follow-up. A reminder is posted 4 weeks later and if there is no response the participant is telephoned by research centre staff around 2 weeks later if there is staff capacity. If

© Copyright: The University of Oxford and Oxford University Hospitals NHS Foundation Trust 2016

no questionnaire is received or telephone contact made a short version of the questionnaire (MTQ14) is posted at around nine weeks with a reminder two weeks later. All questionnaires can be completed online with the Research Data Capture (REDCap) system where up to three reminders are sent by email. There is a SOP (Follow-up of men : Part III Questionnaire follow-up).

COVID-19 issues
UoB shut their buildings (24th March 2020) except for tasks that could not be done remotely and on a limited basis. Returned paper questionnaires (MTQ13) are logged to generate reminders, make calls or send the short version (MTQ14).

Amendments
- Initial reminders might be later than 4 weeks.
- Telephone calls to remind participants might be later than 2 weeks after reminders and subject to capacity.
- The short version (MTQ14) may be later than 9 weeks than the original questionnaire.
- Telephone call by research centre staff to non-responders to offer to complete the questionnaire over the telephone as some participants may not be able to leave their homes.

4. Participant interviews

A sample of participants in each treatment group and of non-randomised men already recruited to a qualitative sub-study will be interviewed if major prostate cancer disease events occur to evaluate men's experiences of outcomes and the implementation and acceptability of active monitoring. Interviews will be conducted either at UOB, or in men's homes or by telephone (as men prefer) by central research staff.

COVD-19 issues

UoB shut their buildings (24th March 2020) preventing interviews at UoB and visits to homes not possible, with staff capacity issues preventing interviews by telephone.

Amendment

Interviews paused (24th March 2020).

22.10 Extended Research Follow-up from April 2022 to March 2027

1. Passive follow-up by notification of vital status from national registries.

Notification from NHS Digital to central research team for participants who consented to registry flagging. Vignettes created for cause of death committee to ascertain the underlying cause of death using medical record reviews at centres (or remote access to Electronic Health Record (EHR)) by research staff and, if available, with local PIs after median-20 year follow up reached. Death certificate data will be used to derive overall survival.

2. Passive follow-up for clinical outcomes.

NHS Digital routine data received by central research team. Outcomes will be derived from routine data by central research team. Medical record reviews at centres at end of follow up period targeted to supplement and validate routine data conducted by research centre staff (or remotely with EHR access), with local PIs.

3. Participant questionnaires for patient-reported outcomes until December 31st 2022

© Copyright: The University of Oxford and Oxford University Hospitals NHS Foundation Trust 2016

A shorter questionnaire posted to participants annually from the research centre (MTQ13) up to 15 years of individual follow-up. A reminder is posted 4 weeks later and if there is no response the participant is telephoned by research centre staff around 2 weeks later, if there is staff capacity. If no questionnaire is received or telephone contact made a short version of the questionnaire (MTQ14) is posted at around nine weeks with a reminder two weeks later. All questionnaires can be completed online with the Research Data Capture (REDCap) system where up to three reminders are sent by email. There is a SOP (Follow-up of men : Part III Questionnaire follow-up). Data collection will cease in December 2022.

23. CLINICAL FOLLOW-UP (CC)

The treatment phase of the trial is completed and all participants are under the clinical care of their local NHS team. Clinical follow-up will take place at 3-monthly intervals in the first year, and at clinical discretion thereafter. Clinical follow-up will be delivered by the specialist or their team (including the trial research nurses) undertaking the delivery of the treatment arms. These appointments will involve assessment of response to treatment, management of any complications, and investigation of any apparent disease progression. Any clinical follow-up is recorded as an event on the study clinical database in the follow-up module. Any PSA tests conducted in the radiotherapy arm up to 6 months post-treatment should be viewed with caution and the results may increase following cessation of hormone therapy.

24. OUTCOME MEASURES

Trial outcomes remain unaltered in extended follow-up, unless stated below

24.1 Primary Outcome

The primary outcome is definite or probable prostate cancer specific mortality (including definite or probable intervention-related mortality) at a median of 10, 15, and 20 years following randomisation**.**

24.2 Secondary Outcomes

- Overall survival at a median of 10, 15, and 20 years follow-up
- Other clinical outcomes at up to 10, 15, and 20 years follow-up: disease progression; treatment complications
- Urinary and bowel symptoms, quality of life, sexual function, depression and other psychosocial effects up to 15 years follow up

These outcomes will be evaluated in the following ways:

1. Prostate cancer mortality – An independent cause of death committee, blinded to the CAP allocation will be convened regularly to scrutinise vignettes and investigate/confirm the underlying cause of death.
2. Overall survival: cause of death from death certificates
3. Disease progression - using PSA, DRE, ultrasonography, biopsy, imaging.

© Copyright: The University of Oxford and Oxford University Hospitals NHS Foundation Trust 2016

4. Treatment complications – (immediate complications relating to primary radical treatments have been completed by 2017 whilst long term complications are ongoing) delayed treatment complications including (completed: blood loss), rectal/bowel (completed: injury/) symptoms, sexual dysfunction, (completed: urethral stricture), lower urinary tract symptoms and treatment, incontinence and obstruction, will be collected from routine data and in clinical schedules and Completed by 15 year analysis: participant questionnaires, developed in the feasibility study.
5. Participant questionnaire: General health status - measured by the validated instrument: EuroQol EQ-5D-3L.[25] Completed by 15 year analysis.
6. Depression and psychological state - measured by the EORTC QLQ-C30 questionnaire completed at annually.[39] Completed by 15 year analysis.
7. Urinary symptoms - measured by the ICSmaleSF questionnaire[22], which includes voiding and incontinence scores, nocturia, frequency and urinary-specific quality of life, the ICIQ questionnaire[40] and items from the UCLA expanded prostate cancer index (EPIC).[35] Completed by 15 year analysis.
8. Sexual function – measured by the UCLA EPIC.[35] Completed by 15 year analysis.
9. Quality of life (QoL) related to prostate cancer treatment – measured using the UCLA EPIC[35]. Completed by 15 year analysis.
10. Qualitative evaluation of outcome - assessed by in-depth interviews with samples of participants in each arm of the trial and also the preference groups. Completed by 15-year analysis:
11. Resource use (NHS, social service and personal). Routine hospital and primary care data sources with additional questions in clinical and participant questionnaires. Completed by 10 year analysis:

25. ECONOMIC EVALUATION

Completed in 10 year analysis

The details of the economics aspects of the study are given in full in the ProtecT Economics Protocol: Evaluating the effectiveness of treatments for clinically localised prostate cancer. The economic evaluation will be conducted from the societal viewpoint as costs associated with the treatment and care of cancer participants may fall on participants, carers, social services and society in general, as well as on the NHS. The evaluation will also be performed using a long run perspective: this is most appropriate to any change in national practice. In this trial, all participants will essentially be receiving a higher level of care than would be usual practice. The aim within this trial is not to determine the efficiency of these treatments relative to current practice nor to determine the efficiency of prostate cancer treatment relative to other forms of health treatment. Rather, the aim is to provide an internal comparison of the three forms of treatment and to assign costs to the cancer detection.

The precise form of the economic evaluation will depend upon the outcomes of the trial. Initially outcomes of the alternative forms of treatment will be compared and consideration will be given to performing a cost-effectiveness or cost-utility analysis. If, for example, there are differences only in survival, a cost-effectiveness analysis will be performed using years of life gained as the measure of outcome. If there are, additionally, differences in quality of life then a cost-utility analysis will be performed, with Quality-Adjusted Life-Years (QALYs) being used as the measure of outcome. These will be formed by combining information about survival with the EuroQol EQ-5D data collected annually, and participant utilities/willingness to pay data. The economic evaluation will be conducted after the main study assessments.

© Copyright: The University of Oxford and Oxford University Hospitals NHS Foundation Trust 2016

25.1 Data Collection

During the trial, the direct costs falling on health services, participants, carers and social services as a result of treatment will be identified and collected. Although cancer detection costs will be the same in all arms of the trial, and are therefore not relevant to the decision about which treatment to perform, information about the costs associated with cancer detection will undoubtedly be useful to policy makers and will therefore be collected during the study. Physical resource use information collected will include: hospital stay, staff time, consumables, diagnostic tests, drugs, capital equipment, GP time and travel, participant and carer travel, out-of-pocket expenses, and any use of social services. Information about the indirect costs and benefits associated with time lost, from both work and leisure, will also be collected. These indirect costs will be presented separately. Routine information systems will be used wherever possible to collect information about both hospital and community services resource use. Resource and cost data from published literature and observational data sources will also be collected to assess whether there are differences between the trial population and routine practice. Where routine systems are available, resource use data will be collected for all participants. Where routine data systems are not available a combination of participant-held diaries and participant and carer questionnaires will be used to assess resource use on a sample of participants from across the centres over the recruitment period. Wherever possible, unit cost data generated within the hospital will be used to value resource use. Pro-rata salary will be used to value staff time. Unit costs of health and social services will be used as a source for the valuation of community/primary care services[33]. Time lost from work will be valued on the basis of average wages, lost leisure time will be evaluated at a proportion of time lost from work.

25.2 Analyses

The analysis from the viewpoint of society will not include any transfer costs/payments. Discounting will be undertaken at 6% (with the discount rate varied during the sensitivity analysis). The economic data collected as part of the trial will be analysed to assess the mean costs, survival and health related quality of life for the specific trial population over the timeframe of the trial (1, 5, 10 and 15 years). This will give a reliable estimate of the relative value for money of the different treatments for the specific population, trial centres and trial protocol.

A sensitivity analysis will be undertaken (particularly given that much of the data will be collected in a somewhat artificial trial situation) and attention will be given to generalising the results obtained beyond the trial.

26. QUALITATIVE RESEARCH

Completed by 15-year analysis. See section 22.9 for details of extended follow-up from 2018 to March 2022 as objectives 2 and 3 are ongoing

The qualitative studies will include:

1. *Completed:* development and implementation of training methods, including tape-recording of information appointments and rapid feedback to ensure high levels of randomisation
2. *Ongoing*: detailed study of men's experiences of undergoing each of the treatments
3. *Ongoing*: evaluation of the implementation and acceptability of the active monitoring treatment programme

© Copyright: The University of Oxford and Oxford University Hospitals NHS Foundation Trust 2016

4. *Completed*: views and perceptions of urologists participating in the study.
5. *Completed*: Reasons of men refusing to participate in prostate cancer detection.

27. DATA MANAGEMENT AND SECURITY

Some updating to processes in extended follow-up 2018 to March 2022
A unique file identified by the study number will be maintained for participants. All data recorded on paper relating to the participant will be located in these files. A list will be maintained at each centre of staff with authorisation to make alteration to the study records, including the study database.

Data obtained on paper will also be entered onto and maintained on an electronic trial database. Information capable of identifying individuals and the nature of treatment received will be held in the database with passwords restricted to ProtecT study staff. Data from computerised sources will be converted to trial databases and hard copies will be maintained in the relevant participants file e.g. PSA results, in locked filing cabinets. Information capable of identifying participants will not be removed from Bristol or clinical centres or made available in any form to those outside the study. Data moved electronically from clinical centres to Bristol will only be sent by secure NHS networks or encrypted.

Routine NHS data will be held in a 'safe haven' on the UOB network, with access restricted to relevant trial staff. Importing, storage and use of routine data will comply with a System Level Security Policy between Public Health England or NHS Digital and UOB.

The Clinical Centres' Database will be closed by 2018 (an archived copy will remain in a secure area of each centres' NHS Trust network) and moved to UOB.

All data held in Bristol will conform to the University of Bristol Information Security Policies and Compliance with the Data Protection Act policies.

The Trust's Caldicott Guardian should be informed at the commencement of the study in a clinical centre.

28. MANAGEMENT, MONITORING AND STUDY ORGANISATION

See revised section 22.9 and below for extended follow up from 2018 to March 2027. A Trial Management Plan (figure 6) below details the responsibilities of University of Oxford and the central research team at UOB.

Trial Management Plan in extended follow up from 2018 to March 2027

● = Accountable for task ⦿ = Responsible for task

	UO	UoB
**1. Study Governance**		

© Copyright: The University of Oxford and Oxford University Hospitals NHS Foundation Trust 2016

1.1	Undertake role of Sponsor as defined by the Research Governance Framework for Health and Social Care and the Medicines for Human Use (Clinical trials) Regulations 2004, as amended	●⦿	
1.2	Maintaining registration of trial on publicly accessible database	●⦿	
1.3	Production and distribution of Sponsor Standard Operating Procedures (SOPs)	●⦿	
1.4	Production of study specific working practice documents, which are compliant with Sponsor SOPs	●	⦿
1.5	Approval and sign off of study specific working practice documents	●⦿	
1.6	Updating Trial Master File and Site Investigator Files	●⦿	
1.7	Coordination of Trial Steering Committees (or equivalent)	●⦿	
1.8	Ensuring appropriate insurance cover in place to cover trial-related responsibilities	●⦿	
**2. Data Management**			
2.1	Database management and data monitoring, including involvement appropriate systems for data capture	●	⦿
2.2	Revision of Case Report Forms (CRFs) and questionnaires	●	⦿
2.3	Validation of trial database, ensuring validation process is appropriately documented	●	⦿
2.4	Raise data queries and resolve at sites	●	⦿
2.5	Ensure data queries are resolved	●	⦿
2.6	Comply with security and confidentiality agreements of suppliers of routine data (e.g. NHS Digital and PHE)	●	⦿
2.6	Conducting interim and final data analyses	●	⦿
2.7	Provision of statistical support	●	⦿
**3. Study Conduct**			
3.1	Preparation and submission of amendments to regulatory applications	●	⦿
3.2	Ensure that conditions and principles of GCP are satisfied and adhered to	●	⦿
3.3	Ensure that the trial is conducted in accordance with the protocol and subsequent amendments	●	⦿
3.4	Approval and sign off of amendments to regulatory applications	●⦿	
3.5	Liaising with participating sites	●	⦿
3.6	Central coordination and management of essential trial documents and patient data collected from participating clinical sites	●	⦿
3.7	Financial monitoring: Monitoring and recording the income and expenses	●⦿	⦿ UOB specific funds
3.8	Financial Management: Using financial monitoring reports to: pay invoices; review and submit financial reports to funders e.g. ASTOX	●⦿	⦿ UOB specific funds
**4. Study Close-Out**			

© Copyright: The University of Oxford and Oxford University Hospitals NHS Foundation Trust 2016

4.1	Submission of relevant annual and end of study reports (e.g. National Research Ethics Service (NRES), Trial Steering Committees)	●⦿	
4.2	Submission of reports for Funder	●⦿	
4.3	Preparation and submission of final publication	●	⦿
4.4	Review and contribute towards reports in 4.1, 4.2 and 4.3 above	●	⦿
4.5	Ensure that all trial records are archived appropriately on conclusion of the trial	●⦿	

A Trial Steering Committee will oversee the ProtecT trial. Written records will be taken of each meeting and copies held by the study coordinator. (Data Monitoring Committee ceased after publication of primary outcomes – see 28.2).

28.1 Trial Steering Committee 2012

Continued in extended follow-up in revised format see below:

- Chair: Professor M Baum (external surgeon, London)
- Professor A Zeitmann (external oncologist, USA)
- Professor D Dearnaley (clinical oncologist, London)
- Dr J Adolfsson (external urologist, Sweden)
- Professor P Albertsen (external urologist, USA)
- Professor T Roberts (external health economist, Birmingham)
- Dr M Robinson (ProtecT uro-pathologist, Newcastle-upon-Tyne)
- Professor M Mason (ProtecT oncologist, Cardiff)
- ProtecT Principal investigators (Professors Hamdy, Donovan, Neal)
- ProtecT senior statistician (Professor T Peters, Bristol)
- ProtecT coordinator (Dr A Lane, Bristol)
- ProtecT and CAP health economist (Dr S Noble, Bristol)
- ProtecT Coordinating Nurses (Mr P Holding, Sheffield; Ms T Lennon, Newcastle)
- Professor R Martin (CAP Principal investigator, Bristol)
- Dr E Turner (CAP coordinator, Bristol)
- Professor J Sterne (CAP senior statistician, Bristol)
- Professor F Schroder (CAP external urologist, The Netherlands)
- Professor T Walley (HTA Director)

The TSC will meet annually in January.

In the extended follow-up period a streamlined Committee with a new independent Chair Professor Deborah Ashby (Imperial College, University of London) will meet annually face to face in 2019 and thereafter by teleconference/videoconference or in person.

Committee

Independent Chair: Professor Deborah Ashby (statistician, London)

Independent member: Dr Chris Parker (oncologist, RMH, Sutton)

© Copyright: The University of Oxford and Oxford University Hospitals NHS Foundation Trust 2016

Independent member: Mr Tom Walton (urologist, Nottingham)

Patient and Public representative: Mr Timon Colegrave

Non-independent member: Chief Investigator (Professor Freddie Hamdy, Oxford)

Observers: Co-PIs (Professors Jenny Donovan, Bristol; David Neal, Oxford), Trial Manager (Professor Athene Lane, Bristol), Trial Statistician (Professor Chris Metcalfe, Bristol) and Sponsor (University of Oxford) and others at the request of the TSC.

28.2 Data Monitoring Committee (DMC 2012)

The committee disbanded after their last meeting on 31/01/2015

- Chair: Professor I Roberts (trialist, London)
- Professor D Ashby (statistician, London)
- Dr R Cowan (oncologist, Manchester)
- Mr T O'Brien (urologist, London)

The DMC will be convened at any point when there are questions of safety or ethics in any part of the trial and will be the only body responsible for instigating an interim analysis of study data. They will review the safety and disease progression of participants in each treatment arm. The DMC will meet annually unless otherwise necessary. A report will be sent to the TSC with the recommendations from each DMC meeting. The TSC can invite the DMC Chair representative to attend the TSC.

28.3 Regional Management Committees

Now disbanded

Each hub centre (Universities of Bristol, Cambridge and Oxford) will have a regional management committee comprised of the principal investigator based at that hub centre, urologists, oncologists, pathologists and lead nurses for each of centres associated with that hub. These committees will assist in monitoring the progress of the study at each centre. Written records will be maintained of these regional meetings and a copy sent to the study co-ordinator, who may attend these meetings as requested.

28.4 Study Management Committee Meetings

Now disbanded

The principal investigators, urologists, oncologists, pathologists and lead nurses for each of centres may have meetings to feedback the TSC and DMC committee findings.

28.5 Specialist Sub-group Meetings

Now disbanded

Specialist sub-group meetings will be held as determined by the sub-groups and PIs:

- Urologists: once per year

© Copyright: The University of Oxford and Oxford University Hospitals NHS Foundation Trust 2016

- Oncologists and radiographers: as required to include the radiotherapy link nurses
- Pathologists: once to twice per year
- Research Nurses:

  Lead Nurses three times per year with research staff

  All nurses: as required for training and updates
- Administrators: as required for all staff

28.6 Management Executive Committee

Continuing in extended follow up

- Professors Hamdy, Donovan, Neal (not in extended follow up: Mason and Peters) comprise the committee
- All publications using ProtecT data must be approved by the committee prior to submission of the publication
- The committee retains the decision to publish or communicate study results
- The content of all presentations at scientific meetings using ProtecT data must be notified to the committee prior to presentation
- The details of publications and presentations at scientific conferences should be notified to the study coordinator and a copy of the paper sent on publication
- All additional studies with ProtecT participants must be approved by the committee prior to commencement, including ProtecT participants with negative biopsies. It is inappropriate for men to enter other urological-related randomised trials whilst in ProtecT follow-up.

28.7 Departures from Protocol

During the extended follow up period:
It is important to keep participant withdrawals from the trial to a minimum but;

- a participant may be withdrawn from the study by their general practitioner or the study team at any time should it be considered detrimental to the participant to continue.

- a participant may withdraw from the study at any time without prejudice to his subsequent treatment.

During the extended follow-up reasons for withdrawal will be documented on the study database.

Completed: Participants who fail to attend appointments will be contacted by telephone and letter, to encourage them to attend, to arrange alternative appointments and to determine reasons for withdrawal. Reasons for withdrawal will be fully documented on the study database and adverse event forms completed if applicable.

28.8 Organisation of Study Documentation

In extended follow clinical centres will have a Site Investigator File ("investigators Trial Master File") which will be updated by University of Oxford staff in conjunction with the Principal Investigators at sites.

© Copyright: The University of Oxford and Oxford University Hospitals NHS Foundation Trust 2016

All clinical centres will have an investigators' Trial Master File which will include all relevant information and documentation for the trial. This will include the protocol, LREC approval, financial agreements, CVs of all staff involved in the trial, delegation logs and any correspondence or emails received pertaining to the study. It will be the responsibility of the lead nurse and clinical secretary at each site to maintain this file.

28.9 Study Monitoring and SMART

Completed The study will be also be monitored by the study co-ordinator and data managers through reports, visits and examination of the study database. Visits to PCC and the study centres may occasionally be made by the research study team as part of the data quality assessments. The annual Site Monitoring and Review Team comprised of two Lead Nurses and the study coordinator will investigate the conduct of the study at each centre.

28.10 HTA Monitoring Visits

Completed

The HTA may make annual monitoring visits regarding the conduct and progress of the study. The meeting will take place immediately following the TSC.

29. PUBLICATION POLICY

Continuing in extended follow-up with updates shown Brief annual reports will be produced for the NIHR HTA until funding ceases in March 2022. Papers will be prepared for publication in general and urological peer-reviewed journals. The findings will also be presented at national and international conferences. The primary analyses will be undertaken when there is a median of 10,15 and 20-year follow-up (i.e. end of year 13, 18 and 23). During the study, a number of other publications have been published such as the effectiveness of the training programme on randomisation rates, the accuracy of PSA tests, urological workload in terms of confirmatory tests required following PSA testing, short-and medium-term outcome following each of the treatments. There is a ProtecT publication policy available on the study website http://www.bristol.ac.uk/population-health-sciences/projects/protect/which describes the procedures to ensure the security of the primary and secondary outcome data of the ProtecT study, and promote analysis and publication of data through the study and other allied studies. Collaborators wishing to use ProtecT data for publication or collect additional data must complete a ProtecT publication or allied study request proforma (available on the study website) for approval by the ProtecT PIs.

30. QUALITY ASSURANCE PROCEDURES

In extended follow up from 2018 to March 2027
The study may be monitored, or audited in accordance with the current approved protocol, GCP, relevant regulations and standard operating procedures. There will also be central monitoring and training of staff to ensure high quality data collection. The Trial Management Group (TMG) is comprised of the CI, Co-PIs and trial

© Copyright: The University of Oxford and Oxford University Hospitals NHS Foundation Trust 2016

coordinator. The TMG will meet in person six-monthly and by skype/teleconference more regularly. The trial receives academic support from the Bristol Trials Centre, a UKCRC Registered Clinical Trials Unit.

31. ETHICAL AND REGULATORY CONSIDERATIONS

In extended follow up from 2018 to March 2027

31.1 Declaration of Helsinki

This study is conducted in accordance with relevant regulations and the principles of the Declaration of Helsinki.

31.2 Guidelines for Good Clinical Practice

The Investigators will ensure that this study is conducted in accordance with relevant regulations.

31.3 Approvals

The protocol, informed consent form, participant information sheet and participant questionnaires were submitted to an appropriate Research Ethics Committee (REC), and HRA for written approval. The Chief Investigator will submit and, where necessary, obtain approval from the above parties for all substantial amendments to the original approved documents.

31.4 Reporting

The CI shall submit an Annual Progress Report, or on request, to the REC Committee, host organisation and Sponsor. In addition, an End of Study notification and final report will be submitted to the same party.

31.5 Participant Confidentiality

Study staff will ensure that the participants' anonymity is maintained. Participants will be identified by a unique ID number on all study documents and any electronic database with clinical data, except for CRFs, where participant initials are added (and date of death if relevant) and participant questionnaires where men add their name to ensure correct logging on questionnaire return. The administrative details of participants are separated from clinical databases. All documents will be stored securely and only accessible by study staff and authorised personnel. The study will comply with the Data Protection Act which requires data to be anonymised as soon as it is practical to do so. Information capable of identifying participants will not be removed or made available to those outside the study.

31.6 Expenses and Benefits

Completed by 15 year analysis: Reasonable travel expenses will be reimbursed on production of receipts, or a mileage allowance provided, as appropriate for participant interviews.

32. FINANCE AND INSURANCE

In extended follow up from 2018 to March 2027

32.1 Funding

The ProtecT study was funded by the NIHR Health Technology Assessment Programme (96/20/99) until 31/03/2022 and is funded by the University of Bristol until December 31st 2022.

© Copyright: The University of Oxford and Oxford University Hospitals NHS Foundation Trust 2016

32.2 Insurance

The University of Oxford has a specialist insurance policy in place which would operate in the event of any participant suffering harm as a result of their involvement in the research (Newline Underwriting Management Ltd, at Lloyd's of London).

33. PROJECT MILESTONES

Completed at end of recruitment in 2009

---

year 1

- ♦ Continue full-scale recruitment in Sheffield, Newcastle and Bristol
- ♦ Train three new centres from September 2001 to March 2002 (Birmingham, Cardiff, Edinburgh)
- ♦ Train three new centres from March to September 2002 (Leeds, Leicester and Cambridge) if requirements are complied with, e.g. availability of 3-D conformal radiotherapy
- ♦ Initiate 6-monthly meetings of steering group to evaluate recruitment, co-ordination between centres and data quality control.
- ♦ Follow-up of all participants to be continued throughout lifetime of study

year 2

- ♦ Continue full-scale recruitment in Sheffield, Newcastle, Bristol and three centres commencing in year 1, with increasing recruitment in the next three centres

years 3 to 5

- ♦ Continue full-scale recruitment in all nine centres – to be completed at the end of year 5

© Copyright: The University of Oxford and Oxford University Hospitals NHS Foundation Trust 2016

34. REFERENCES

1. Majeed A, Babb P, Jones J, Quinn M. *BJU Int* 2000;85:1058-1062.
2. Wasson JH, Sushman CC, Bruskewitz RC, et al. *Arch Fam. Med* 1993;2:487-93.
3. Editorial. UK experts advise against prostate cancer screening. *Lancet* 1997;349 (9050):1-2.
4. Woolf SH. *BMJ* 1997;314:989-90.
5. Neal DE, Donovan JL. *Lancet Oncology* 2001: 17-24.
6. Paulson DF, Lin GH, Hinshaw W, Stephani S. *J Urol* 1982;128:502-5.
7. Graversen PH, Nielsen KT, Gasser TC, et al. *Urology* 1990;36:493-8.
8. Madsen P, Peder H, Gasser TC, Corle DK. *Scand J Urol Nephrol* 1988; 110: 95-100.
9. Wilt TJ, Brawer MK. *J Urol* 1994;152:1910-4.
10. Wilt TJ, Brawer MK. *Semin Urol* 1995;13:130-6.
11. O'Reilly P, Martin L, Collins G. *BMJ* 1999;318(7197):1556.
12. Livesey JE; Cowan,RA; Brown,CW. *Clinical Oncology* 2000; 12:63.
13. *Prostate cancer incidence statistics*. 2014; Available from: http://www.cancerresearchuk.org/health-professional/cancer-statistics/statistics-by-cancer-type/prostate-cancer/incidence.
14. Lane JA, Donovan, JL, Davis M, et al. *Lancet Oncol*, 2014;15:1109-18.
15. Hamdy FC, Donovan JL, Lane JA, et al. *N Engl J Med*, 2016;357:1415-24.
16. Donovan JL, Hamdy FC, Lane JA, et al. *N Engl J Med*, 2016;357:1425-37.
17. NEJM. *Notable Articles of 2016*. 2016; Available from: https://anesthesia.ucsf.edu/system/files/NEJM%20Notable%20Articles%202016.pdf
18. Altmetric. *Top 100 Articles of 2016*. 2016; Available from: https://www.altmetric.com/top100/2016/
19. Donovan JL, Frankel SJ, Faulkner A, Selley MA, Gillatt D, Hamdy FC. *BMJ* 1999; 318:299-300.
20. Albertsen P. *Journal of Urology*, 2016;196:1604-05.
21. D'Amico AV. *J Clin Oncol*, 2017;35:1638-40.
22. Donovan JL, Peters TJ, Abrams P, Brookes ST, De La Rosette JJ, Schafer W. *J Urol* 2000;164:1948-55.
23. Brazier JE, Harper R, Jones NMB, *et al. BMJ* 1992; 305:160-164.
24. Zigmond AS, Snaith RP. The Hospital Anxiety and Depression Scale. *Acta Psychiat Scan* 1983; 67:361.
25. The EuroQol Group. EuroQol - a new facility for the measurement of health. *Health Policy* 1990;16:199-208.
26. Shacham S. *J Pers Assess*, 1983;47:305-6.
27. Schröder FH, van der Cruijsen-Koeter I, de Koning HJ, *et al. J Urol* 2000;163(3):806-12.
28. Babaian RJ, Toi A, Kamoi K, *et al. J Urol* 2000;163:152-7.
29. Presti JC Jr, Chang JJ, Bhargava V, Shinohara K. *J Urol* 2000;163:163-6; discussion 166-7.
30. Borboroglu PG, Comer SW, Riffenburgh RH, Amling CL. *J Urol* 2000;163:158-62.
31. Hermanek P, Sobin LH, Wittekind C. *TNM Classification of Malignant Tumours (UICC S.), 2002.*
32. Häggman MJ, Macoska JA, Wojno KJ, Osterling JE. *J Urol* 1997; 158: 12-22.
33. Catalona WJ, Partin AW, Slawin KM, *et al. JAMA* 1998 20;279:1542-7.
34. Patel D, White PA, Milford Ward A. *BJU Int* 2000;85(6):686-9.
35. Wei JT, Dunn RL, Litwin MS, et al. *Urology*, 2000;56:899-905.
36. Walsh PC. *Campbell's Urology*. Ed. Walsh PC, Retik AP, *et al*. WB Saunders. Philadelphia. pp 2865-2886.
37. *Lahtinen T, Tenhunen M, Vayrynen M*. Radiother Oncol *1993;28(2):174-6.*

© Copyright: The University of Oxford and Oxford University Hospitals NHS Foundation Trust 2016

38. Hamdy FC*. Lancet Oncology*, 2011;12:832-3.
39. Aaronson NK, Ahmedzai S, Bergman et al. The EORTC QLQ-C30: a quality of life instrument for use in international clinical trials in oncology. *JNCI 1*993;85:365-76.
40. Avery K, Donovan JL, Peters TJ et al. *Neurourol Urodyn*, 2004;23:322-30.
41. Frankel SJ, Donovan JL, Peters TJ *et al. J Clinical Epidemiology*, 1998; 51(8): 677-685.

© Copyright: The University of Oxford and Oxford University Hospitals NHS Foundation Trust 2016

APPENDIX 1: SETTING UP A NEW CLINICAL CENTRE

Completed at end of recruitment in 2009

Resources have been provided by the NHS HTA Programme to support all research costs. Each clinical centre will be sub-contracted to one of the major research hubs (Bristol, Cambridge and Oxford). Resources will be obtainable by quarterly invoice in arrears to the hub centres. First wave centres will commence in September 2001; second wave in March 2002.

1. Lead nurse and secretary to be appointed as close to day 1 as possible. The lead nurse will be employed by the research hub, but all other staff will be appointed through the Trust.
2. Appointments and setting up of study to be assisted by the close involvement of the lead nurses from the research hubs. Assistance will also be provided by the ProtecT qualitative researcher.
3. <u>Setting up ProtecT study</u> SOP to be utilised.
4. Clinical centre' lead nurse (CCLN) to shadow co-ordinating nurses in the research hub for two days. Birmingham, Cardiff, Leicester and Leeds to Sheffield; Edinburgh and Cambridge to Newcastle.
5. CCLN to identify first practice, set up lab staff/procedures, TRUS/biopsy clinics, office procedures etc.
6. CCLN to schedule first prostate check clinic (PCC) appointments hourly in first instance. Two days' worth, then stop for discussion with co-ordinating nurses.
7. Two-day training programme for new nurses at Bristol, new secretaries and urologists if possible.
8. Two new nurses to be appointed by month 3. Secretary also to be appointed. One nurse to be appointed by month 9 to help with follow-up and PCCs.
9. The PIs and coordinator to work with urologists and lead nurses over the first few months to ensure they are aware of the study details, budget arrangements and provide training for the eligibility appointments and the information appointments where participants request second opinions.
10. PCC appointments will be extended to 45 minutes during the training period. A minimum of 60 appointments per week, each lasting approximately 30 minutes, is expected when the centre is working at full capacity (i.e. after six months).

© Copyright: The University of Oxford and Oxford University Hospitals NHS Foundation Trust 2016

Training new centres for the information appointment and randomisation

Full training for nurses and urologists will be based on the findings of the feasibility study. It is expected that at least 60-70% of participants will consent to randomisation following training. Training will include:

1. Initial 2-day course outlining the study procedures, need for a treatment trial, evidence about treatments, concepts and practicalities of randomisation, and practice in the delivery of the study information.
2. Observing information appointments led by training coordinator nurses in the research hubs - 'mentoring'.
3. Consenting to the tape-recording of 'information' appointments.
4. Receiving feedback and further training based on the analysis of the tape-recordings.
5. Accepting that the randomisation rate will be monitored and consenting to further tape-recordings and feedback during the progress of the study.

The training programme is based on the feasibility study.

If the randomisation rate is <60% after the first 6 months, every effort will be made by the ProtecT study team to increase recruitment to the acceptable rate required for the study sample size (minimum 60%). If this rate can not be reached an alternative centre will have to be found to replace the centre.

© Copyright: The University of Oxford and Oxford University Hospitals NHS Foundation Trust 2016

APPENDIX 2: SAMPLE SIZE CALCULATIONS AND STATISTICAL ANALYSES (2012) AND UPDATED FOR EXTENDEND FOLLOW-UP (2018 To March 2027)

The latter part of the feasibility study showed that each centre can see approximately 200 prostate check clinic attenders and thus detect approximately 4 localised cases per month (48 per year). The table below indicates the numbers of eligible cases expected based on five years of recruitment in nine centres in total (i.e. six new centres in addition to the current three), assuming that each new centre will require six months of training and will operate at 0.5 efficiency over first 12 months.

Centres	Feasibility	Year 1	Year 2	Year 3	Year 4	Year 5
Current 3	150	144	144	144	144	144
4+5+6		72	144	144	144	144
7+8+9			100	144	144	144
Total	150	216	388	432	432	432
Cum.	150	366	754	1186	1618	2050

Sample size implications

Sample size could be considered in terms of survival time or the proportion expected to survive after 10 years' follow-up. Although the former is preferable since it corresponds to the primary analysis, given the high proportions surviving for 10 years the power of these two approaches is virtually the same. Given the availability of data in the literature on the 10-year survival (around 85% for all treatments) and the greater transparency of such specifications, the following calculations are therefore presented in these terms. In time this will need to be revisited once sufficient numbers of events accrue: (a) for the figures to be presented more directly for the intended primary Cox regression analyses, and (b) for the study assumptions about such numbers of events to be reassessed in a similar fashion to that employed by the ATAC trial involving postmenopausal women with early breast cancer. In the meantime, for both the primary (intention-to-treat) analysis including all men randomised, and hence also for the purposes of considering the implications of the projected sample size, the crucial statistic is the 95% confidence interval for the difference in 10-year survival between any two of the three treatment arms.

Previously the central role of such confidence intervals was expressed in terms of demonstrating equivalence between the trial arms, but the present proposal is that it is better to take a more general view – that is, by considering the widths of projected confidence intervals for various scenarios. The first set of scenarios is to obtain the confidence intervals for a spread of possible observed differences (between the null and 10 percentage points), given the current projected total sample size of 2050 to be recruited by May 2006 (Year 5). An additional aspect considered for the first set of scenarios is the impact of a relatively conservative Bonferroni correction to the coverage probability of the confidence intervals, to account for the three pair wise comparisons being considered.

The second set of scenarios involves calculating the increases in the sample size that would be required in order to reduce the widths of such confidence intervals by 10% and 20% in relative terms. The third set of scenarios explores the potential precision of the main (explanatory) secondary analysis – in particular, by

© Copyright: The University of Oxford and Oxford University Hospitals NHS Foundation Trust 2016

calculating the confidence intervals for a simple (‘per protocol’ or ‘on-treatment’) analysis including only those who actually received their allocated treatment, assuming percentages for the latter of between 75% and 85%.

For all the scenarios, the figures initially presented are the numbers of men analysed (for the intention-to-treat analyses this is the same as the number randomised). Assuming the current overall rate of 70% of men agreeing to be randomised, the figures are then multiplied by about 1.43 to give the total number of men with localised prostate cancer who need to be identified by the detection.

In detail, then, for the first set of scenarios the confidence interval widths were calculated for the current projected sample size of 478 per arm for (again projected) observed differences of 0, 5 and 10 percentage points. In each case the overall percentage surviving 10 years was assumed to be 85% – for example, the difference of 10 percentage points related to a comparison of 80% versus 90%. However, the effect of altering the overall survival was investigated for selected situations by also considering 85% vs. 95%. In each case the margin of error is presented, calculated as usual as the ‘half-width’ of the confidence interval.

Within the range considered, the observed difference had negligible impact on the absolute margin of error, and hence just one figure is presented in the following table. Moreover, the 4.5% margin of error for 80% vs. 90% only reduces to 3.8% for 85% vs. 95%. It should nonetheless be noted that the implications of the levels of imprecision given below may well change across, for example, a confidence interval of –4.5% to 4.5% for an observed difference of 0% and one of 5.5% to 14.5% around a 10% difference. Adjusting for the three pairwise multiple comparisons has very little effect. (Although this has been conducted relatively crudely by just altering the (two-sided) significance level to 5% divided by 3 and hence the coverage probability to 98.3% for each contrast, if anything this approach would be expected to be conservative.) As can be seen from the second set of scenarios, sample sizes would have to be increased considerably to yield relatively modest reductions in the margin of error – for instance, to reduce it by 10% would require an increase of 25% in the numbers randomised, and for a 20% gain the increase is 60%. For each of the two assumed percentages of men adhering to their allocated treatment the third set of scenarios indicate that the (most conservative) secondary on-treatment analyses would have a margin of error of around 5% (with the 4.9% reducing to 4.1% for 85% vs. 95% surviving at 10 years).

Scenario		Margin of error	Number per arm analysed	Total number identified
Observed difference in range 0-10 percentage points	Ignoring multiple testing	4.5%	478	2050
	Adjusting for multiple testing	5.5%	478	2050
Reduce margin of error by a factor of:	10%	4.1%	≈ 600	2574
	20%	3.6%	≈ 750	3216
On-treatment analysis excluding departures from protocol (% included)	85%	4.9%	406	2050
	75%	5.2%	358	2050

Data analysis plan

Primary analysis

The primary comparative analysis will be conducted on an intention-to-treat basis, comparing the three groups as randomised. For the primary analysis of survival (for a median of 10 years’ follow-up), Cox proportional

© Copyright: The University of Oxford and Oxford University Hospitals NHS Foundation Trust 2016

hazards regression will be used to obtain hazard ratios and their confidence intervals, adjusting for the four stratification/minimisation variables (centre, age, PSA and Gleason). Corrections for multiple comparisons between the three randomisation groups will be considered, by for instance using the Student-Newman-Keuls procedure.

Presentation of the primary analyses

Treatment	10-year mortality risk	95% confidence interval
Surgery	a.bc	(j.kl, m.no)
Radiotherapy	d.ef	(p.qr, s.tu)
Active monitoring	g.hi	(v.wx, y.za)
p-value	*0.fg*	
p-value	*0.fg*	

This table will be supplemented by the Kaplan-Meier plots. The p-value is for the (overall) null hypothesis of equal risk across the three treatments. The hazard ratios and their 95% CIs for the three pairwise comparisons are then presented in a separate table or text, but pairwise significance tests are conducted if and only if the overall test yields a p value less than 0.05. This conditional approach keeps the overall false positive rate at 5%, and has been found to maintain power in simulation studies (Bauer, P. Multiple testing in clinical trials. Statistics in Medicine 1991;10:871-890.)

As a secondary analysis, the hazard ratio and 95% CI for both radical treatments (surgery and radiotherapy) combined versus active monitoring will be presented. Any p-value for this comparison would need to be corrected for multiple comparisons to maintain consistency between the primary and secondary analyses; the precise nature of this correction is to be investigated in further simulation studies.

Secondary analyses

Secondary analyses will include Cox regression for time to disease progression and logistic regression for survival at 10 years, and analyses of the various quality of life instruments employed within the trial. The latter are complicated by the fact that while men enter the ProtecT study in general terms healthy and asymptomatic, each treatment is likely to impact in different ways on particular physical, social and emotional measures. The principal quality of life measure has therefore been chosen as the SF-12 since it assesses generic health status and hence should apply equally across the randomised groups. The other important measures (of incontinence, sexual and bowel function, anxiety and depression) will all be analysed, but many of these will vary across the groups in relatively predictable ways. Further methodological work is envisaged and clearly required to investigate the importance placed by men themselves upon these various aspects of quality of life, both severally and in combination. The EORTC QLQ-C30 is included to examine the impact of progression, and the EuroQoL EQ-5D to assess utilities for the economic evaluation. Adjustments will also be made for major imbalances between the arms at baseline by introducing appropriate covariates into all the regression models.

Planned subgroup analyses will be conducted by stratified analyses for descriptive statistics and formally by including interaction terms in the relevant regression models. These subgroup analyses will investigate differential comparisons across the randomisation groups according to the following patient characteristics: disease grade (Gleason score <7, 7-10), clinical stage (T1 vs. T2), age and PSA level (both as continuous variables). The 'Gleason score' will be obtained as the sum of the Gleason score for the most dominant pattern in the tumour and the score for the second most common pattern. For subgroup analyses based on continuous

© Copyright: The University of Oxford and Oxford University Hospitals NHS Foundation Trust 2016

variables (age and PSA), departures from the assumptions of a linear relationship will be investigated by introducing polynomial terms, with a categorical version only considered if necessary on grounds of interpretability and provided there is no marked loss of power.

The secondary analyses will also estimate the relative efficacy of each treatment amongst individuals who do comply with their original allocated treatment. Such an estimate can be considered as a measure of the treatment's potential if, for example, compliance with treatment can be improved through a reduction in the risk of side-effects. Per protocol and on-treatment methods attempt to measure efficacy, but both are almost inevitably biased. Instead we will obtain unbiased estimates of efficacy amongst compliers using complier average causal effect (CACE) methods, extended to the analysis of survival data. CACE methods are based on two key assumptions: (i) random allocation ensures that, on average, there are an equal number of non-compliers in each study arm; (ii) the effect of the most conservative treatment is the same irrespective of whether the patient was allocated to that treatment or opted for it after being allocated to a more radical alternative. We will employ the C-PROPHET implementation of this approach (Loeys T, Goetghebeur E. A causal proportional hazards estimator for the effect of treatment actually received in a randomized trial with all-or-nothing compliance. Biometrics 2003;59:100-5). This method requires that patients allocated to the more conservative treatment cannot then undergo the more radical alternative. Hence in this secondary analysis we will make the two comparisons between active monitoring and each of surgery and radiotherapy in turn, and we will make the simplifying assumption that patients moving from active monitoring to radical treatment are doing so as part of the active monitoring protocol (i.e. these patients are not swapping between treatment arms, but are moving along the active monitoring treatment pathway). These analyses will be adjusted for the four minimisation variables, as done for the primary analysis.

Secondary analyses will also explore the impact of inaccurate clinical staging prior to treatment, for (effectively) observational studies comparing the treatment options. Clinical staging is acknowledged to be inaccurate, with approximately 25-30% of cases found not to be localised to the prostate when full operative staging is carried out. We will thus have the most accurate (pathological) staging only in one treatment arm (radical surgery), but it is likely that similar levels of upstaging will be occurring in the other arms.

Further secondary analyses will compare estimates using data from the cohort of men who refused random allocation and chose their treatment (the preference cohort) to the estimates from the primary analyses of the randomised trial.

Extended follow-up from 2018 to March 2027
The statistical methods and analysis for the primary analyses were described in the published version of the protocol and Statistical Analysis Plan, published on http://hdl.handle.net/1983/14265b05-5d00-40b2-90c7-020db8ee725a. Analyses for the median 15 year analyses are detailed below and were reviewed by the NIHR HTA in the extension funding application The 15 year median analysis Statistical Analysis Plan is published at https://doi.org/10.17605/OSF.IO/JCT49. Analyses for the 20 year median analyses will follow similar approaches as for the 15 year analyses.

Description of Statistical Methods (2018 to March 2027)

The statistical analysis of primary[3] and secondary[4] outcomes conducted for the median 10-year follow-up will be repeated for the median 15-year and 20 year follow-up. In brief, the primary outcome of deaths due to prostate cancer or its treatment will be compared among the three assigned treatment groups on an intention-to-treat basis with the use of Cox proportional hazards regression adjusted for trial centre, age at baseline, Gleason score, and PSA level at baseline (log-transformed). The results of a cumulative incidence

© Copyright: The University of Oxford and Oxford University Hospitals NHS Foundation Trust 2016

approach, which will avoid bias due to other causes of death, will also be presented (although there was no evidence of such bias in the primary 10-year analysis). This approach will be adapted as necessary for secondary event-based outcomes, e.g. date of disease progression, date of diagnosis of metastatic disease, and all-cause mortality. The previously specified sub-group analyses will be conducted, investigating whether the relative treatment effects vary by age, clinical stage, Gleason score, and PSA level at diagnosis. Patient reported outcomes completed during the complete 11-year follow-up will be compared among the three assigned treatment groups on an intention-to-treat basis. Two-level random-effects models will be used to accommodate the correlation between the repeated assessments for each man. Two-level linear models will be used for continuous measures, and two-level logistic models were used for binary measures. All models will include trial centre, age at baseline, Gleason score, and PSA level at baseline (log-transformed) as covariates. All men with at least one post-randomisation measure available will be included in an analysis; there are no plans to impute missing assessments, although the possible effect of these on the observed results will be investigated through sensitivity analyses.

The Number of Participants

The original sample size calculation was presented in published protocol. The ProtecT study randomly allocated 545 men to active monitoring, 553 men to surgery, and 545 men to radiotherapy. Amongst these men, at median 10-year follow-up, there were 8, 5 and 4 prostate cancer deaths respectively. There were very few withdrawals at the time of the median 10-year analyses in randomised men[3]. A simple extrapolation from the occurrence of prostate cancer deaths during the median 10-year follow-up, suggests that by the end of 2020 the number will have doubled from 17 to 34 among those randomised. If there is an increase in the incidence rate of prostate cancer death, as may result from the number of metastatic disease cases that have been diagnosed, then conditional power of 67%, 78%, and 85%, would result from the occurrence of 48, 60, and 72 prostate cancer deaths respectively.

A formal sample size calculation was not undertaken for men in other groups (advanced cancer, comorbidities or with and initial PSA of 20ng/ml or above). All men in these groups will be followed up observationally.

Analysis of Outcome Measures

The analyses will be conducted as described above.

© Copyright: The University of Oxford and Oxford University Hospitals NHS Foundation Trust 2016

APPENDIX 3: PROGRESSION OF PARTICIPANTS THROUGH PROTECT STUDY

Completed in 2009

Note: X is an exit point, participant does not continue in the ProtecT study.

Participant states pre-PCC

Returns invitation letter or telephones

1. Yes
2. No = X
3. Refuses = X

PCC list of appointments

1. Attends
2. Did not attend, rebook and then put as refusal after 2 times = X
3. Refuses = X

States at end of PCC clinic

1. Refuse Consent form 1 points 1-4 = X and blood destroy if PSA done, once men have result
2. Refuse Consent 1 blood tests = X and blood destroy if PSA taken done, once men have result
3. Refuse Consent 1 points 2 /3, continue in ProtecT, but flag not use blood for other studies
4. Ineligibility = X and blood destroy if PSA taken once men have result
5. Eligible and consent 1 obtained for ProtecT and ProMPT for consent 2

States after return of Consent form 3

1. Refuses consent = X
2. Consent given for PSA test
3. Exit ProtecT study as negative PSA = X
4. Eligible for diagnostic phase as raised PSA

NB 1-3 return all participants' questionnaires and schedules to Bristol inside PCC Schedule

States after diagnostic phase

1. Localised prostate cancer, PSA 3-19ng/ml and fit to continue
2. Participant refusal to attend biopsy appointment, rebook 2x , if no = X
3. Advanced cancer = X
4. HGPIN alone on biopsy = X
5. Biopsy negative, no HGPIN, PSA free/total <0.12, 2nd biopsy = clinical and patient decision

NB: Some of these men will have prostate cancer on subsequent biopsies. The decision to offer more biopsies will depend on repeat PSA, and at the discretion of the clinician with a fully informed patient. If they have localised prostate cancer later, they can be included in ProtecT.

© Copyright: The University of Oxford and Oxford University Hospitals NHS Foundation Trust 2016

6. Biopsy negative, no HGPIN, PSA free/total ≥0.12 = X
7. PSA 10-20ng/ml, bone scan, metastases = X
8. PSA 10-20ng/ml, bone scan, localised
9. Ill-health exclusion = X
10. Other exclusion = X

NB 2-10 return all participants' questionnaires and schedules to Bristol inside PCC Schedule

States after Information appointment

1. Randomised to three arm
2. Randomised to three arm, refuses and expresses preference
3. Selection option, no randomisation
4. No decision, further appointment(s) arranged
5. Withdrawn = X

NB 1-5 return all participants' questionnaires and schedules to Bristol inside PCC Schedule.

© Copyright: The University of Oxford and Oxford University Hospitals NHS Foundation Trust 2016

APPENDIX 4: AMENDMENT HISTORY

Version 3.0 and a list of changes made from prior versions are available online in supplementary material (pdf pages 85-86) to the NEJM clinical outcomes paper http://www.nejm.org/action/showSupplements?doi=10.1056%2FNEJMoa1606220&viewType=Popup&viewClass=Suppl

Amendment No.	Protocol Version No.	Date issued	Author(s) of changes	Details of Changes made
	5.5	28/02/2022	JA Lane	Extension to study to March 31st 2027 for 20-year median analysis of clinical outcomes by passive follow-up
	5.4	11/05/2021	JA Lane	Study end date extended from June 2021 to March 31st 2022
	5.3	18/11/2020	JA Lane	Questionnaires ceasing at individual's 15 years, 15-year SAP publication
	5.2	01/05/2020	JA Lane	COVID-19 amendments to follow-up
	5.1	22/03/2019	JA Lane	Revised Trial Steering Committee
	5.0	18/06/2018	JA Lane	Data collection changes and study conduct in extended follow up phase, new sponsor protocol template
	4.0	17/11/2017	JA Lane	Participant questionnaire and PROMs
	3.0	21/10/2012	JA Lane	See above
	2.4	01/07/2009	JA Lane	See above
	2.3	20/06/2007	JA Lane	See above
	2.2	01/06/2005	JA Lane	See above
	2.1	01/06/2003	JA Lane	See above
	2.0	01/12/2002	JA Lane	See above
	1.0	01/09/2001	JA Lane	See above

© Copyright: The University of Oxford and Oxford University Hospitals NHS Foundation Trust 2016

Summary of changes to the ProtecT Trial Protocol 2001-2022

Changes to Protocol version 2.1, June 2003

- The option for participants to take part in a two-arm trial of radiotherapy and surgery only (including in the feasibility study) was removed in 2003, at the request of the TSC (Section 5, p9 of the first protocol). This is not included in the final 2012 protocol.
- The process for randomising general practices to be asked to participate in the ProtecT trial as part of the larger CAP trial of screening was added (Section 7.1, p9 of the final protocol).
- The inclusion criteria were clarified as follows: (a) age of participant defined on the date of the preparation of the list in the general practice, (b) we added *'and with a life expectancy of 10 years'* to *'fit for any of the three treatments'* and (c) we clarified the date of registration at the general practice. Also included was the invitation of men 45-49 years in one centre as part of a pilot study (Section 8, p9 of the final protocol).
- The exclusion criteria were clarified by formally excluding those with 'bilateral hip replacement' (Section 8, p10 in final protocol).
- The Prostate Check Clinic (recruitment) process was clarified in detail using updated Standard Operating Procedures, including enabling participants to consent to blood collection for the ProMPT (Prostate Mechanisms of Progression and Treatment) study (Section 10, pp.10-13 in final protocol), clarifying processes to be followed by research nurses (Section 10.1 p12 in the final protocol), and clarifying that men with PSA>19.9ng/ml should be investigated for reasons of a high PSA, such as prostatitis before proceeding to biopsy (Section 10.3 p13 in final protocol).
- Details of the diagnostic phase were clarified using updated Standard Operating Procedures, including providing greater detail for recruitment staff in relation to criteria for eligibility based on PSA test findings of <10ng/ml, 10-19.9ng/ml, and over 19.9ng/ml (Section 11.1 to 11.4, p13-15 of the final protocol), and adding a flow chart to illustrate the process before eligibility is determined (Section 11, p17 of the final protocol). In addition, the Trial Steering Committee requested further Quality Assurance for histopathological interpretation of tissue samples, and an internal audit process was included (Section 11.7, p18 of the final protocol).
- The formal criteria to be assessed to confirm eligibility for randomisation were presented more clearly (they were not changed) (Section 12.1 p18 in the final protocol).
- The categorisation of minimisation variables was clarified – Gleason score 2-4, 5-7 or 8-10; and PSA tests <10ng/ml or 10 or more (Section 14.1 p19 in the final protocol).
- Treatment options were described for progressing disease in the AM arm (presented in the treatment pathway in the final protocol, p23).
- Details of the neo-adjuvant hormone treatment, CT planning, radiotherapy dosage, delivery, outlining, organs at risk, and dose specification were clarified following updating of the radiotherapy protocol (Section 18 pp.26-30 in final protocol). The radiotherapy Quality Assurance process was also added and described (p29) – now in press (Mason et al, Clin Oncol 2016, manuscript attached).
- Details of adverse events and research follow-up timescales were clarified and added (Section 20.2, and 20.4 in the final protocol, p31).
- A description of the Cause of Death and adjudication committee was added (Section 22.2, p33 of the final protocol).
- Analysis at five years was added, subject to DMSC/TSC approval. This was later removed by DMSC/TSC and so does not appear in the final protocol.
- Quality of life, previously measured by the FACT-P questionnaire was replaced by UCLA prostate cancer index (later called EPIC) in 2003; and referred to as UCLA EPIC (Section 22.2, p37 in the final protocol).
- The Statistical Analysis Plan (SAP) was clarified following advice from DMSC. The final version of the SAP was published in 2015 (Metcalfe C, Peters T, Hamdy F. Prostate Testing for Cancer and Treatment (ProtecT) Study: Statistical Analysis Plan. Version 1.0. Bristol, UK, University of Bristol 2015: 1-22 – attached).

Changes to Protocol version 2.2, August 23rd 2005

- A requirement was added for an isotope bone scan to be conducted if the Gleason score of the cancer at diagnosis was 8-10 (Section 11.2 on p14 of the final protocol).
- In men who have a negative biopsy at recruitment, we clarified that further PSA testing would not be offered within the ProtecT study beyond the age of 70 years. (Section 11.2, p15 of the final protocol).
- We clarified that diagnostic staging would be defined according to the TNM 2002 classification (section 11.2, p15 of the final protocol).
- We clarified that if the Gleason score was missing, it would be classified as '6' for minimisation purposes (Section 14.1 p20 of final protocol).
- In the Active Monitoring arm, we removed the criterion of a rise in PSA level of >20% between consecutive measures, and maintained the rise of 50% over a 12 month period as a trigger for further review. (Section 16.2, p21 of the final protocol).
- Definition of biochemical progression and details of the management of progressing disease in the radiotherapy arm was updated according to the ASTRO-Phoenix criteria (section 18.12 p30 of the final protocol, and within the pathway flow-chart).

Changes to Protocol version 2.3, June 20th 2007

- The exclusion criteria were clarified to exclude men who had moved to another primary care physician practice so that they could be enrolled only once (section 8, p10 of the final protocol).
- Treatment pathway flowcharts and treatment pathways after treatment failure were added (pp 23, 25 and 30 of the final protocol).
- Laparoscopic and robot-assisted laparoscopic radical prostatectomy were added as techniques allowed in the study (section 17, p24 of the final protocol).
- A new follow-up process was added so that participants who moved away from the study centre could be contacted and tracked formally (section 27, p32 of the final protocol).
- Profile of Moods States and Impact of Events questionnaires (pre-randomisation to evaluate aspects of screening) were added (section 22, p34 of the final protocol).
- Resource-use questionnaire for carers/partners of randomized participants added (Section 23.1 p35 of final protocol).

Changes to Protocol version 2.4, July 1st 2009

- New methods of communication were introduced to follow-up non-responders to outcome questionnaires, with additional contact details collected (Section 20.6, p32 of final protocol).
- Addition of the EORTC QLQ-C30 questionnaire at 2 and 5 years' follow-up (section 22.2, p34 of final protocol).
- Clarification that follow-up starts from the date of randomisation (section 20.4, p31 of the final protocol).
- Improved wording to clarify primary outcome as prostate cancer-specific mortality at 10 years' median follow-up (section 22, p33 of final protocol).

Changes to Protocol version 3.0, October 21st 2012

- Advice that men should receive annual isotope bone scans if the PSA had risen above 9.9 ng/ml was added, even if there was no change from the past 12 months (pathway flow-chart updated, p23 of final protocol).
- Advice that men taking the finasteride (which lowers PSA values) should have their PSA values adjusted for the active monitoring PSA kinetics was added, in line with clinical practice (Section 16.2, p22 of the final protocol).
- The requirement for annual bone scans if the PSA had risen above 9.9 ng/ml even if there is no change from the past 12 months was added. (section 16.3, p22 of the final protocol)
- The EORTC QLQC-30 questionnaire was added at 10 years' follow-up (Section 22.2, p34 of the final protocol).
- The five-year interim analysis was removed.
- The sub-groups for the analysis of Gleason scores were clarified as two: <7 and 7-10 (Appendix 2, p44 of the final protocol).

Changes to Protocol version 4.0, November 17th 2017
Rationale for changes:<br>• To update the protocol with the results from the median 10-year analysis, published in NEJM in 2016<br>• To stop data collection by research nurses and rely primarily on routine NHS electronic data following a decision from the NIHR HTA Programme to reduce funding.<br>• The Sponsor (University of Oxford) required the protocol to be more consistent with their new template.<br><br>Detailed changes were:<br>• Updated background to the trial including the results from the median 10-year outcome publications.<br>• Updated processes for collection of clinical follow-up data from routine NHS electronic sources, supplemented by selected medical note verification at centres by trial staff.<br>• Immediate complications of treatment had occurred and were reported previously, and collection was ceased. Long-term impacts of treatments continued to be collected in participant questionnaires.<br>• Adverse event reporting was ceased.<br>• Updated statistical analysis plan for the median 15-year follow-up analysis.<br>• Health economic data collection was ceased because of reduced funding.
**Changes to Protocol version 5.0, June 18th 2018**
Rationale for changes:<br>• Updating the study questionnaire for the collection of PROMs (Patient-Reported Outcome Measures) based on findings from the results published in 2016.<br><br>Detailed changes were:<br>• Reduced respondent burden by removing some items/measures from the main questionnaire.<br>• Produced a new short version focusing on the key measures for participants who did not respond to initial mailings.<br>• Updated methods for questionnaire administration, including postal delivery, sending a reminder letter if no reply was received within two weeks and identification of contact person if other methods were unsuccessful.<br>• Updated timing of interviews for participants in the qualitative study to focus on major prostate cancer disease events.
**Changes to Protocol version 5.1, March 22nd, 2019**
Detailed changes were:<br>• At the funder's request, the membership of the independent Trial Steering Committee was updated with meetings to occur face-to-face or by teleconference, as appropriate.<br>• Updating of one measure in the study questionnaire.
**Changes to Protocol version 5.2, May 1st, 2020**
Rationale for changes<br>• COVID-19 pandemic<br><br>Detailed changes were:<br>• Cause of death committee suspended (24th March 2020 – national lockdown).<br>• Non-contact follow-up implemented, including notification of vital status from national registries, and follow-up of clinical outcomes by Urology local investigators using electronic remote access to Trust Electronic Health Records for case-note reviews<br>• Administration of study questionnaires adjusted because of remote working of staff<br>• Participant interviews paused (24th March 2020, as above)

Changes to Protocol version 5.3, December 12th 2020
Changes were:<br>• Publication of the median 15-year Statistical Analysis Plan.<br>• Study questionnaire administration to be ceased following a participant's completion of 15 years of follow up. Letter and newsletter sent to inform participants.
**Changes to Protocol version 5.4, May 12th 2021**
Changes were:<br>• It was necessary to extend the study end date to March 31st, 2022, due to COVID-19 pandemic delays.
**Changes to final Protocol version 5.5, February 28th 2022**
Changes were:<br>• Study follow-up to be continued to a median of 20 years, extending the study end-date to March 31st, 2027.

Metcalfe, C., Peters, T., & Hamdy, F. (2015). Prostate Testing for Cancer and Treatment (ProtecT) Study: Statistical Analysis Plan. (1.0 ed.) University of Bristol.

Publisher's PDF, also known as Final Published Version

Link to publication record in Explore Bristol Research
PDF-document

University of Bristol - Explore Bristol Research

General rights

This document is made available in accordance with publisher policies. Please cite only the published version using the reference above. Full terms of use are available: http://www.bristol.ac.uk/pure/about/ebr-terms.html

Take down policy

Explore Bristol Research is a digital archive and the intention is that deposited content should not be removed. However, if you believe that this version of the work breaches copyright law please contact open-access@bristol.ac.uk and include the following information in your message:

• Your contact details
• Bibliographic details for the item, including a URL
• An outline of the nature of the complaint

On receipt of your message the Open Access Team will immediately investigate your claim, make an initial judgement of the validity of the claim and, where appropriate, withdraw the item in question from public view.

Bristol Randomised Trials Collaboration

University of BRISTOL

Bristol Randomised Trials Collaboration (BRTC)

Prostate Testing for Cancer and Treatment (ProtecT) Study

Statistical Analysis Plan

Version 1.0 (19th November 2015)

The following people have reviewed the Statistical Analysis Plan and are in agreement with the contents			
**Name**	**Role**	**Signature**	**Date**
Chris Metcalfe	Author	Chris Metcalfe	19/11/2015
Tim Peters	Statistical Reviewer	T. J. Peters	19.11.15
Freddie Hamdy	Chief Investigator	[illegible]	19/11/2015

Table of Contents

1. INTRODUCTION & PURPOSE 4
2. SYNOPSIS OF STUDY DESIGN AND PROCEDURES 5
2.1. Trial objectives and aims 5
2.1.1. Primary objective 5
2.1.2. Secondary objectives 5
2.2. Trial design and configuration 5
2.3. Trial centres 5
2.4. Eligibility criteria 6
2.4.1. Inclusion criteria 6
2.4.2. Exclusion criteria 6
2.5. Description of interventions 6
2.6. Randomisation procedures 7
2.7. Sample size and justification 7
2.8. Blinding 7
2.9. Trial committees 7
2.10. Outcome measures 8
2.10.1. Primary outcome 8
2.10.2. Secondary outcomes 8
2.11. Interim analysis 9
3. GENERAL ANALYSIS CONSIDERATIONS 9
3.1. Analysis populations 9
3.2. Procedures for missing data 9
3.3. Study centre effects 10
3.4 Acceptance of allocated intervention 10
4. DESCRIPTION OF PARTICIPANT CHARACTERISTICS 10
4.1. Disposition 10
4.2. Baseline characteristics 10
5. ASSESSMENT OF STUDY QUALITY 11
5.1. Eligibility checks 11
5.2. Protocol deviations 11
6. ANALYSIS OF EFFECTIVENESS 11
6.1. Summary of primary and secondary outcomes 11
6.2. Primary analysis 12
6.3. Secondary analyses 12
6.4. Subgroup analyses 13

6.5. Sensitivity analysis 13
7. OTHER ANALYSES 14
7.1. Patient preference cohort 14
7.2. Upgrading and upstaging following radical surgery 14
7.3. Treatment effects in those able to comply with their allocation 14
8. FINAL REPORT TABLES AND FIGURES 15
9. APPENDIX 22
10. REFERENCES 22

1. INTRODUCTION & PURPOSE

This document details the statistical analysis proposed and the presentation that will be followed, as closely as possible, when analysing and reporting the main results from the **Prostate Testing for Cancer and Treatment (ProtecT) Study**.

The core of this statistical analysis plan is unchanged from that laid out in the protocol (Version 1.0, September 2001), with subsequent changes due to clarifications and further detail. Hence the key decisions had been made and documented prior to unblinded analyses being conducted in confidence for the Data Monitoring Committee.

The purpose of the plan is to:

1. Make explicit the details of the planned analysis, as agreed with the Trial Steering Committee.
2. Ensure that the analysis is appropriate for the aims of the trial, reflects good statistical practice, and that interpretation of *a priori* and post-hoc analyses is appropriate.
3. Explain in detail how the data will be handled and analysed to enable others to perform the actual analysis in the event of sickness or other absence, or to replicate the analyses

Additional exploratory or auxiliary analyses of data not specified in the protocol are permitted but fall outside the scope of this analysis plan (although such analyses would be expected to follow Good Statistical Practice).

The analysis strategy will be made available if required by journal editors or referees when the main papers are submitted for publication. Additional analyses suggested by reviewers or editors will, if considered appropriate, be performed in accordance with the Analysis Plan, but if reported the source of such a post-hoc analysis will be declared.

Amendments to the statistical analysis plan will be described and justified in the final report of the trial.

2. SYNOPSIS OF STUDY DESIGN AND PROCEDURES

IMPORTANT: *This synopsis is purely to provide background information for those reading the statistical analysis plan. It does not replace the study protocol; the current version of which must be consulted for all other purposes.*

2.1. Trial objectives and aims

The ProtecT trial was designed in the late 1990s and early 2000s to compare the major conventional treatments for patients with clinically localised prostate cancer detected through population-based PSA testing. The three treatments were radical prostatectomy, external beam three-dimensional (3D) conformal radiotherapy, and active monitoring.

2.1.1. Primary objective

In men with localised prostate cancer detected through population-based PSA testing, to compare definite or probable prostate cancer specific mortality (including definite or probable intervention related mortality) at a median of 10 years following random allocation to radical prostatectomy, external beam three dimensional (3D) conformal radiotherapy, and active monitoring.

2.1.2. Secondary objectives

To make the same comparison on a number of secondary outcome measures, including overall survival, clinical disease progression, treatment complications, lower urinary tract symptoms, quality of life, and sexual function. To estimate the resource use and costs of case-finding, treatment and follow-up, and to compare costs and outcomes of treatment in terms of survival and health related quality of life.

2.2. Trial design and configuration

A three parallel groups randomised controlled trial.

2.3. Trial centres

Recruitment to the trial took place at general practices in and around nine study centres across the UK: Newcastle, Sheffield, Bristol, Cardiff, Edinburgh, Birmingham, Leicester, Cambridge, and Leeds.

2.4. Eligibility criteria

2.4.1. Inclusion criteria

- Men
- Age 50-69 years on the date of preparation at the general practice of the list of potential participants
- Able to give written informed consent to participate
- Fit for any of the three treatments and with a life expectancy of at least 10 years
- Registration with the participating general practice on the date of the PCC
- For randomisation: clinically localized prostate cancer (confirmed by isotope bone scan in men with PSA of 10ng/L or more) diagnosed by 10-core biopsy following a PSA level of 3ng/L or more.

2.4.2. Exclusion criteria

- Concomitant or past malignancies (other than a small treated skin cancer)
- Prior treatment for prostate malignancy
- Serious cardiac or respiratory problems in the previous 12 months of the PCC, e.g. stroke, MI, heart failure, COPD
- Kidney dialyses or transplantation
- Bilateral hip replacement
- Previous entry to the ProtecT study at a prior general practice
- PSA 20ng/L or more at diagnosis

2.5. Description of interventions

The **Active Monitoring Protocol** aimed to avoid immediate radical treatment whilst assessing the disease over time, with a review and the opportunity for radical treatment if there was evidence of disease progression. PSA levels were measured and reviewed every three months in the first year and twice yearly thereafter. Changes in PSA levels were assessed, and a rise of at least 50% over the previous 12 months triggered repeat testing within six to nine weeks. If the PSA levels were persistently raised, or the patient had other concerns, a review appointment was made to consider treatment options.

The **Radiotherapy Protocol** began with neoadjuvant androgen suppression, given for three to six months before and concomitantly with 3D-conformal radiation therapy delivered at 74 Gy in 37 fractions.

Surgery was a radical retropubic prostatectomy procedure. The surgical approach was left to the discretion of the surgeon, and was most commonly open, but laparoscopic, or robot-assisted approaches were permitted from 2003.

2.6. Randomisation procedures

Once a man agreed to have his treatment determined by random allocation, the research nurse telephoned a central system for a computer-generated allocation. Randomisation was stratified by centre with stochastic minimization by age at invitation, Gleason score (primary and secondary grades), and mean of baseline and first biopsy PSA results. Men who declined randomisation were offered identical follow-up, and formed an observational patient preference cohort.

2.7. Sample size and justification

Following a review of the likely disease-specific mortality with active monitoring, the Data Monitoring Committee advised in 2008 that recruitment should continue to a projected target of 1590 (530 per arm). This would enable a risk ratio of 0.54 to be detected with 80% power at the 5% significance level for a pairwise comparison of each radical treatment with active monitoring. This assumes 10% prostate cancer specific mortality at 10 years in men managed with active monitoring, equivalent to an absolute difference of 10% versus 5.4%.

2.8. Blinding

A panel of clinicians, otherwise independent of the study, confirmed cause of death for those deaths known to be at risk of miscoding in death certificates from previous trials of prostate cancer detection (ERSPC) and treatment (SPCG-4). These clinicians were kept blind to each man's treatment allocation, by having the clinician consider information in a vignette extracted from a man's medical record rather than consulting the record directly.

2.9. Trial committees

ProtecT has a Trial Steering Committee, chaired by Professor Michael Baum (University College London). Reporting to that committee is an Independent Data Monitoring Committee, chaired by Professor Adrian Grant (University of Aberdeen) until 2012 and subsequently by Professor Ian Roberts (London School of Hygiene and Tropical Medicine).

2.10. Outcome measures

2.10.1. Primary outcome

The primary outcome is definite or probable prostate cancer mortality, including intervention-related deaths, at a median 10 years' follow-up.

The process used to assess cause of death was adapted from the PLCO algorithm and ERSPC process. The medical records of deceased participants were summarised by trained researchers, anonymised and reviewed by an independent endpoint committee. Table 2 presents the classification of deaths by study arm.

2.10.2. Secondary outcomes

Secondary clinical, economic and patient-reported outcomes to be presented in the primary results papers are:

- overall mortality
- metastatic disease
- primary treatment failure:
    - for radical prostatectomy: PSA 0.2ng/ml+ at three months post-surgery
    - for the radiotherapy protocol: according to the ASTRO criteria (Roach, 2006).
- clinical disease progression
- treatment complications
- resource use (the subject of a separate economic analysis plan)

Metastatic disease is defined as positive imaging showing bony, visceral and/or lymph node metastases, or PSA above 100; or bone marrow infiltration with associated systemic symptoms.

Clinical disease progression will be measured as person-years free of the consequences of disease progression. Signs of disease progression will include evidence of metastatic disease; the initiation of hormone therapy; diagnosis of clinical T3 or T4 disease; or ureteric obstruction, rectal fistula, or the need for a permanent catheter when these are not considered to be a complication of treatment.

The *treatment complications* listed in Table A will be recorded, following the indicated interventions. Complications following radical therapy will be presented irrespective of whether the man was allocated to that intervention, chose it following allocation, or it was recommended after a period of active monitoring.

The patient-reported outcomes which have been measured are listed in the Appendix. These measures are derived from validated questionnaires and have

been completed at recruitment, at first biopsy, six months after randomisation, and yearly thereafter for at least 10 years. These measures will be reported in a separate companion paper, to be submitted for publication at the same time as the primary outcomes paper.

Table A. Serious intervention-related complications occurring between intervention initiation and 90 days after intervention completion, which will be reported following the indicated interventions

	Active monitoring protocol*	Surgery	Radiotherapy protocol
Death	X	X	X
Hospital admission lasting more than 10 days	X	X	
Hospital admission for sepsis	X	X	X
Hospital readmission	X	X	X
Rectal, bowel or bladder injury or damage		X	X
Ureteric injury or damage		X	X
Urethral or anastomotic problem requiring intervention		X	X
More than three units of blood transfused		X	
Thromboembolic-cardiovascular event		X	

*Following a repeat biopsy, for example.

2.11. Interim analysis

A confidential interim analysis, by study arm, of primary and secondary outcomes has been presented to the annual meeting of the Data Monitoring Committee since 2004. The Data Monitoring Committee recommends changes to the Trial Steering Committee if clear evidence (of the order of $p<0.001$) of a positive or negative balance of risks and benefits emerges for one intervention in comparison with the others.

3. GENERAL ANALYSIS CONSIDERATIONS

3.1. Analysis populations

The primary analysis data set is all men randomised to one of the three management options being compared in the ProtecT trial.

3.2. Procedures for missing data

There are no missing data for the minimisation variables. Men are linked for vital status notification with the NHS national registry, ensuring almost all primary

outcome events are captured. When we are notified that a man has emigrated, the man's inclusion in the analysis will be censored on the date of emigration. The number of men emigrating will be presented, broken down by random allocation.

Where a man has omitted responding to a small number of items on a patient reported outcome measure, these will be imputed as per the guidance for that measure. However, where the patient has not responded to any or most of the items on a measure, the main analysis of patient-reported outcomes will NOT be based on data with those missing scale scores imputed. However, the amount of missing data, by allocation arm, will be presented. All men providing at least one post-randomisation patient-reported measure will be included in the relevant analysis.

3.3. Study centre effects

The primary analysis will be stratified by study centre, by using dummy variables in the regression equation to distinguish the nine study centres. For the main trial paper there is no plan to investigate whether estimated treatment effects vary by study centre.

3.4 Acceptance of allocated intervention

With regard to the surgery and radiotherapy protocols, a participant is considered to have accepted their allocated intervention if he has commenced that treatment (in any way) within nine months of randomisation. Similarly, a participant is considered to have accepted the active monitoring protocol if he has undergone at least one PSA test for monitoring within nine months of randomisation and not undergone radical treatment in that time.

4. DESCRIPTION OF PARTICIPANT CHARACTERISTICS

4.1. Disposition

Details of the recruitment of the ProtecT randomised trial cohort, up to the point of randomisation, were presented in the *Baseline Paper* (Lane et al, 2014). Details of how many men were excluded and for what reasons are presented. The subsequent flow of patients through the trial will be summarised in a CONSORT diagram that will include the numbers randomised to the three treatment groups, losses to follow-up and the numbers analysed (Figure 1).

4.2. Baseline characteristics

These are presented as descriptive statistics in the *Baseline Paper* (Lane, 2014).

5. ASSESSMENT OF STUDY QUALITY

5.1. Eligibility checks

The numbers of patients excluded from random allocation of treatment are reported with reasons in the *Baseline Paper* (Lane, 2014).

5.2. Protocol deviations

The first treatment received within nine months of random allocation will be tabulated by allocated treatment, to illustrate the extent of initial non-compliance with randomised allocation (Table 1). Active monitoring commences with the first PSA monitoring test; this does not include PSA tests undertaken whilst waiting for surgery or radiotherapy within eight months of randomisation. Radiotherapy commences with the first fraction.

In addition, the date on which radical treatment is received by those allocated to active monitoring will be recorded. This can be compared with each man's PSA series to identify those changes to radical treatment which did not follow an increase in PSA level, as described in the active monitoring protocol. The cumulative proportion of these who undergo radical treatment over the follow-up period will be plotted against time (Figure 2).

Treatments received by men in each treatment allocation arm following any changes within three years and five years of randomisation will be reported.

6. ANALYSIS OF EFFECTIVENESS

6.1. Summary of primary and secondary outcomes

The following summaries of the primary outcome events will be presented for each treatment allocation arm:

- Number of deaths due to prostate cancer (Table 3).
- Prostate cancer mortality at 5 and 10 years, with 95% confidence interval (Table 3).
- Prostate cancer mortality per 1,000 person years of follow-up, with 95% confidence interval (Table 3).
- Cumulative hazards of death from prostate cancer as a function over time (Figure 3).

The following summaries will be presented for the secondary outcome events (Table 4):

- Number of events.
- Events per 1000 person years of follow-up, with 95% confidence interval.

6.2. Primary analysis

The primary analysis will be conducted on an intention-to-treat basis comparing treatment groups as allocated. Deaths occurring until a median of 10-year follow-up has accumulated (midnight 00:00 on Monday 23rd November 2015) will be included in the locked database.

The primary outcome measure of prostate cancer (definite, probable, or intervention-related) mortality will be compared between the three treatment groups using Cox's proportional hazards regression adjusted for study centre (all nine centres distinguished using dummy variables), age at baseline (continuous measure in years), Gleason score (2-6, 7, 8-10), and PSA at baseline (continuous measure in ng/ml, log-transformed to accommodate positive skewed distribution):

$$h(t) = h_0(t)\exp\{\beta_{1j}x_{1i} + \beta_{2k}x_{2i} + \beta_3 x_{3i} + \beta_{4m}x_{4i} + \beta_5 ln.x_{5i}\}$$

β_{1j} is the log hazard ratio comparing two of the treatment arms, with two of the three pairwise comparisons being available from a single iteration of the analysis (i.e. j=1,2 the estimated comparisons depending on the choice of comparator treatment). x_{1i} is the treatment allocation (0,1,2) for participant i. β_{2k} (k=1 to 8) captures differences in the hazard of the primary outcome event between study centres, x_{2i} being the study centre for participant i. β_3 is the linear effect of age, with x_{3i} being the age in years for participant i. β_{4m} (m=1,2) accommodates the effect of Gleason score category with x_{4i} being the Gleason score category for participant i. Finally β_5 is the linear effect of log-transformed PSA level, x_{5i} being the PSA level at diagnosis for participant i.

The hazard ratio and 95% confidence interval for the treatment effect estimated in each pairwise comparison of allocated treatments will be presented, but pairwise significance tests will only be conducted if a test of an equal 10-year disease specific mortality risk across all three arms yields a p-value of less than 0.05 (Table 3). This conditional approach keeps the overall false positive rate at 5%, and has been found to maintain power in simulation studies (Bauer 1991).

6.3. Secondary analyses

The approach to the primary analysis will be adapted to the analysis of secondary outcome events, i.e. definite, probable or possible prostate cancer mortality; all-cause mortality; and metastatic cancer (Table 4).

Patient reported outcomes (see Section 2.10.2) by allocated groups (i.e. following the ITT principle) will be presented in a separate companion paper. Summary statistics by group will be presented graphically for the baseline, 6 month, 12 month and subsequent assessment points. Analyses will employ multi-level models for repeated measures to estimate average treatment effects. Random intercepts and random slopes models will be considered. These analyses will be adjusted for the stratification (centre) and minimisation (baseline age, Gleason score, PSA level) variables as described in the previous section, and the baseline measure of the patient reported outcome being considered. The exact nature of formal testing of differences between groups depends upon the trends observed over time in each; in selecting an analysis, the principles of the primary analysis will be followed as closely as possible, and the most parsimonious set of parameters chosen to describe the differences between groups over time.

6.4. Subgroup analyses

Pre-specified subgroup analyses will investigate whether treatment effectiveness in reducing prostate cancer specific mortality is modified by factors measured at randomisation:

- age (above versus below 65 years)
- clinical stage (T1 v T2)
- Gleason score (score 6 v 7+)
- PSA at diagnosis (PSA < 10ng/ml v 10 and above)

The statistical models used in the primary analysis will be extended to incorporate interaction terms, to test null hypotheses of no variation in treatment effect across subgroups. For sub-group analyses based on age and PSA, the interaction test will be based on the continuous measure, and departures from the assumption of a linear relationship will be investigated (and accommodated if necessary) by introducing polynomial terms. Significance testing will be conducted with the principles of the primary analysis being followed as closely as possible (Table 5).

6.5. Sensitivity analysis

Sensitivity analyses will repeat the primary analysis, but with the following changes made:

- men recruited during the feasibility study period excluded.
- the outcome will be death definitely, probably **and possibly** due to prostate cancer.

7. OTHER ANALYSES

7.1. Patient preference cohort

The analysis of the primary outcome, of secondary outcome events, and of patient-reported outcomes described above will be applied to data collected from the patient preference cohort. The results of this analysis will be compared to that of the randomised trial data, and any differences highlighted.

7.2. Upgrading and upstaging following radical surgery

Grade and stage, as ascertained at histopathology and following radical prostatectomy will be compared.

7.3. Treatment effects in those able to comply with their allocation

A secondary analysis will estimate the relative efficacy of each treatment amongst individuals who do comply with their original allocated treatment. Such an estimate can be considered as a measure of the treatment's potential if, for example, compliance with treatment can be improved through a reduction in the risk of side-effects. Per protocol and on-treatment methods attempt to measure efficacy, but both are almost inevitably biased. Instead we will obtain unbiased estimates of efficacy amongst compliers using complier average causal effect (CACE) methods, extended to the analysis of survival data (Hampson, 2012).

8. FINAL REPORT TABLES AND FIGURES

Figure 1. CONSORT flowchart, illustrating the flow of participants through each of the three arms of the trial, from the point of randomisation. *As per study protocol.

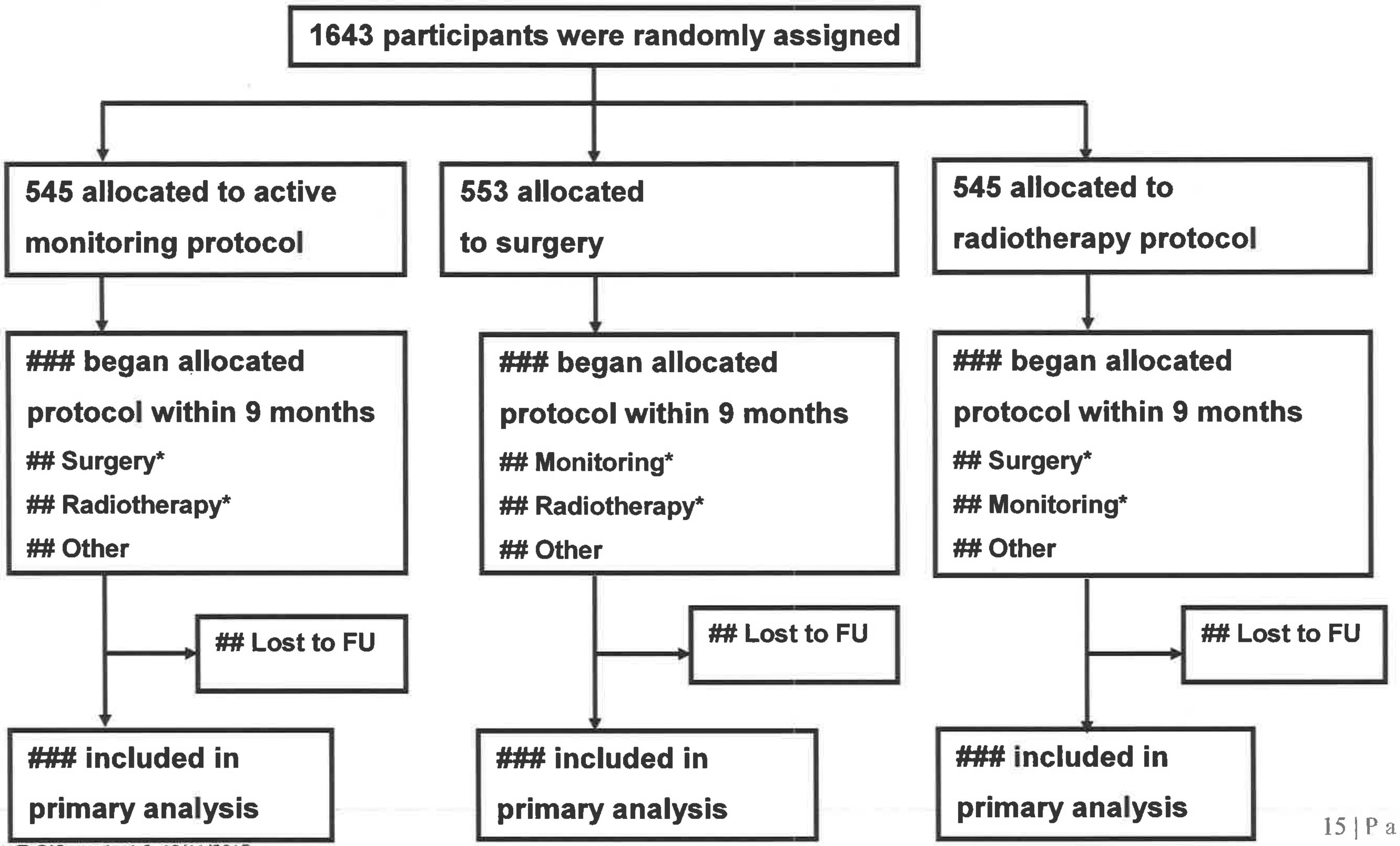

Figure 2. Cumulative probability of being treated radically, for the active monitoring protocol group. The shaded area indicates the proportion of patients who, having followed the active monitoring protocol, changed to the surgery or the radiotherapy study protocol, and the unshaded area non-protocol changes to radical therapy

Figure 3. Cumulative hazards of death from prostate cancer[1] in the active monitoring (solid line), surgery (long dash line) and radiotherapy (short dash line) treatment groups

1. Definitely or probably due to prostate cancer or its treatment, as established by the Independent Cause of Death Evaluation Committee

Figure 4. Cumulative proportion of men over the follow-up period with clinical progression, including metastasis and death due to prostate cancer or its treatment, by randomised treatment group

Table 1. Treatments initiated post-randomisation by random treatment allocation

	Active Monitoring protocol (n=545)	Surgery (n=553)	Radiotherapy protocol (n=545)
First treatment initiated within nine months of randomisation, n (%)			
Active monitoring protocol			
Radical prostatectomy			
Radical radiotherapy protocol			
Other radiotherapy regime			
Other treatment			
No treatment initiated within nine months of randomisation			

Table 2. Likelihood death due to prostate cancer, or treatment of prostate cancer, as established by the independent Cause of Death Evaluation Committee, by randomised treatment group

Cause of death	Active monitoring protocol (N=545)	Surgery (N=553)	Radiotherapy protocol (N=545)
Definite prostate cancer			
Probable prostate cancer			
Possible prostate cancer			
All cause death			

Table 3. Prostate cancer mortality by random allocation

	Active monitoring protocol (N=545)	Surgery (N=553)	Radiotherapy protocol (N=545)	p-value[2]
Total person years in follow-up				
Number of deaths due to prostate cancer[1]				
Percent prostate cancer mortality[1] at 5 years (95% CI)				
Percent prostate cancer mortality[1] at 10 years (95% CI)				
Prostate cancer deaths[1] per 1000 person years (95% CI)				

CI denotes confidence interval

1. Definitely or probably due to prostate cancer or its treatment, as established by the Independent Cause of Death Evaluation Committee

2. Likelihood ratio test of the null hypothesis "no difference in prostate cancer mortality between the three treatment arms", adjusted for study centre, age, mean PSA at prostate check clinic and biopsy, and Gleason score at baseline.

Table 4. Clinical progression, metastatic disease and all-cause mortality, by randomised allocation

	Active monitoring protocol (N=545)	Surgery (N=553)	Radiotherapy protocol (N=545)	p-value[1]
Person years of follow-up free of clinical progression[2]				
Number of men with clinical progression				
Clinical progression per 1000 person years (95% CI)				
Person years of follow-up free of metastatic disease				
Number of men with metastatic disease				
Metastatic disease per 1000 person years (95% CI)				
Total person years in follow-up				
Number of deaths due to any cause				
All-cause deaths per 1000 person years (95% CI)				

[1] Likelihood ratio test of the null hypothesis "no difference in prostate cancer mortality between the three treatment arms", adjusted for study centre, age, mean PSA at prostate check clinic and biopsy, and Gleason score at baseline. [2]Signs of disease progression will include evidence of metastatic disease; the initiation of hormone therapy; diagnosis of clinical T3 or T4 disease; or ureteric obstruction, rectal fistula, or the need for a permanent catheter when these are not considered to be a complication of treatment.

Table 5a. Prostate cancer specific mortality[1], stratified by subgroups

	Rate per 1000pyrs	95% Confidence Interval	P-value[2]
AGE: Below 65 years at randomisation			
Active monitoring protocol			
Surgery			
Radiotherapy protocol			
AGE: 65 years or older at randomisation			
Active monitoring protocol			
Surgery			
Radiotherapy protocol			
PSA AT DIAGNOSIS: Less than 10ng/ml			
Active monitoring protocol			
Surgery			
Radiotherapy protocol			
PSA AT DIAGNOSIS: 10ng/ml+			
Active monitoring protocol			
Surgery			
Radiotherapy protocol			

1. Definitely or probably due to prostate cancer or its treatment, as established by the Independent Cause of Death Evaluation Committee. 2. Likelihood ratio interaction test of the null hypothesis, no difference in relative effectiveness of the three treatments between levels of a subgroup

Table 5b. Prostate cancer specific mortality[1], stratified by subgroups

GLEASON SCORE: 6	
Active monitoring protocol	
Surgery	
Radiotherapy protocol	
GLEASON SCORE: 7+	
Active monitoring protocol	
Surgery	
Radiotherapy protocol	
CLINICAL STAGE: T1c	
Active monitoring protocol	
Surgery	
Radiotherapy protocol	
CLINICAL STAGE: T2	
Active monitoring protocol	
Surgery	
Radiotherapy protocol	

1. Definitely or probably due to prostate cancer or its treatment, as established by the Independent Cause of Death Evaluation Committee. 2. Likelihood ratio interaction test of the null hypothesis, no difference in relative effectiveness of the three treatments between levels of a subgroup

9. APPENDIX

The following standard assessment tools have been completed by men participating in the ProtecT study:

- Expanded Prostate Index Composite
- International Consultation on Incontinence Questionnaire (ICIQ)
- International Continence Society urinary function (ICSmaleSF)
- International Continence Society sexual function (ICSsex)
- EORTC QLQ-C30 cancer-specific impacts
- Hospital Anxiety and Depression Scale
- Short Form 12 (SF-12) mental and physical subscales
- EuroQoL-5D (EQ-5D) generic quality of life

10. REFERENCES

Bauer P. (1991). Multiple testing in clinical trials. **Statistics in Medicine 10:**871-890.

Bokhorst LP, Bangma CH, van Leenders GJLH, et al. (2014). Prostate-specific antigen-based prostate cancer screening: reduction of prostate cancer mortality after correction for nonattendance and contamination in the Rotterdam section of the European Randomised Study of Screening for Prostate Cancer. **European Urology 65:**329-336.

Cuzick J, Sasieni P, Myles J, Tyrer J. (2007). Estimating the effect of treatment in a proportional hazards model in the presence on non-compliance and contamination. **Journal of the Royal Statistical Society, Series B 69:**565-588

Hampson L, Metcalfe C. (2012). Incorporating prognostic factors into causal estimators: A comparison of methods for randomised controlled trials with a time-to-event outcome. **Statistics in Medicine 31:** 3073-3088.

Lane JA, Donovan JL, Davis M, et al for the ProtecT study group. (2014). The ProtecT (Prostate testing for cancer and Treatment) trial: study design, diagnostic process and baseline results. **Lancet Oncology 15:**1109-1118.

Loeys T, Goetghebeur E. (2003). A causal proportional hazards estimator for the effect of treatment actually received in a randomized trial with all-or-nothing compliance. **Biometrics 59:**100-105

Metcalfe C. (2014). Can the results of the European Randomized Study of Screening for Prostate Cancer be decontaminated? **European Urology 65:**337-338.

Roach M, Hanks G, Thames H, Schellhammer P, Shipley WU, Sokol GH, Sandler H. (2006). Defining biochemical failure following radiotherapy with or without hormonal therapy in men with clinically localized prostate cancer: recommendations of the RTOG-ASTRO Phoenix consensus conference. **International Journal of Radiation Oncology, Biology & Physics 65:**965-974.

OPEN ACCESS

University of BRISTOL

Metcalfe, C., Peters, TJ., & Hamdy, F. C. (2020, Nov 24). Prostate Testing for Cancer and Treatment (ProtecT) Study. Statistical Analysis Plan – 15 years: Version 1.0 19th November 2020. https://doi.org/10.17605/OSF.IO/JCT49

Publisher's PDF, also known as Version of record

License (if available):
CC BY

Link to published version (if available):
10.17605/OSF.IO/JCT49

Link to publication record in Explore Bristol Research
PDF-document

This is the final published version of the article (version of record). It first appeared online via Centre for Open Science at dx.doi.org/10.17605/OSF.IO/JCT49. Please refer to any applicable terms of use of the publisher.

University of Bristol - Explore Bristol Research

General rights

This document is made available in accordance with publisher policies. Please cite only the published version using the reference above. Full terms of use are available: http://www.bristol.ac.uk/red/research-policy/pure/user-guides/ebr-terms/

Prostate Testing for Cancer and Treatment (ProtecT) Study

Statistical Analysis Plan – **15 years**

Version 1.0 19th November 2020)

The following people have reviewed the Statistical Analysis Plan and are in agreement with the contents

Name	Role	Signature	Date
Chris Metcalfe	Author		19.11.2020
Tim Peters	Statistical Reviewer		19.11.2020
Freddie Hamdy	Chief Investigator		19.11.2020

Table of Contents

1. INTRODUCTION & PURPOSE 3
2. SYNOPSIS OF STUDY DESIGN AND PROCEDURES 4
2.1. Trial objectives and aims 4
2.1.1. Primary objective 4
2.1.2. Secondary objectives 4
2.2. Trial design and configuration 4
2.3. Trial centres 4
2.4. Eligibility criteria 5
2.4.1. Inclusion criteria 5
2.4.2. Exclusion criteria 5
2.5. Description of interventions 5
2.6. Randomisation procedures 6
2.7. Blinding 6
2.8. Trial committees 6
2.9. Outcome measures 6
2.9.1. Primary outcome 6
2.9.2. Secondary outcomes 6
2.10. Interim analysis 7
3. GENERAL ANALYSIS CONSIDERATIONS 8
3.1. Analysis populations 8
3.2. Procedures for missing data 8
3.3 Definitions of treatment received 8
4. DESCRIPTION OF PARTICIPANT CHARACTERISTICS 9
4.1. Disposition 9
5. ANALYSIS OF EFFECTIVENESS 10
5.1. Summary of outcomes to report at median 15 years follow-up 10
5.2. Prostate cancer mortality at median 15 years follow-up 10
5.3. Other analyses at median 15-years follow-up 11
5.4. Subgroup analyses 12
5.5. Sensitivity analyses 13
6. 15-YEAR PUBLICATION PLAN 14
6.1. Planned papers and timelines 14
7. FINAL REPORT TABLES AND FIGURES 15
8. APPENDIX 19
9. REFERENCES 20

1. INTRODUCTION & PURPOSE

*This document details the statistical analysis proposed and the presentation that will be followed, as closely as possible, when analysing and reporting the median **15-year** results from the **Prostate Testing for Cancer and Treatment (ProtecT) Study**. As far as possible this plan will follow the approaches in the main ProtecT statistical analysis plan written for the primary analysis of median 10-year follow-up, which is available at*: https://njl-admin.nihr.ac.uk/document/download/2021093

The purpose of the plan is to:

1. Make explicit the details of the planned analysis, as agreed with the Trial Steering Committee.

2. Ensure that the analysis is appropriate for the aims of the trial, reflects good statistical practice, and that interpretation of *a priori* and post-hoc analyses is appropriate.

3. Explain in detail how the data will be handled and analysed to enable others to perform the actual analysis in the event of sickness or other absence, or to replicate the analyses

Additional exploratory or auxiliary analyses of data not specified in the protocol are permitted but fall outside the scope of this analysis plan (although such analyses would be expected to follow Good Statistical Practice).

The analysis strategy will be made available if required by journal editors or referees when the main papers are submitted for publication. Additional analyses suggested by reviewers or editors will, if considered appropriate, be performed in accordance with the Analysis Plan, but if reported the source of such a post-hoc analysis will be declared.

Amendments to the statistical analysis plan will be described and justified in the final report of the trial.

2. SYNOPSIS OF STUDY DESIGN AND PROCEDURES

IMPORTANT: *This synopsis is purely to provide background information for those reading the statistical analysis plan. It does not replace the study protocol; the current version of which must be consulted for all other purposes.*

2.1. Trial objectives and aims

The ProtecT trial was designed in the late 1990s and early 2000s to compare the major conventional treatments for patients with clinically localised prostate cancer detected through population-based PSA testing. The three treatments were radical prostatectomy, external beam three-dimensional (3D) conformal radiotherapy, and active monitoring.

2.1.1. Primary objective

In men with localised prostate cancer detected through population-based PSA testing, to compare definite or probable prostate cancer specific mortality (including definite or probable intervention related mortality) at a median of 10 years following random allocation to radical prostatectomy, external beam three dimensional (3D) conformal radiotherapy, and active monitoring.

2.1.2. Secondary objectives

To make the same comparison on a number of secondary outcome measures, including overall survival, clinical disease progression, treatment complications, lower urinary tract symptoms, quality of life, and sexual function. To estimate the resource use and costs of case-finding, treatment and follow-up, and to compare costs and outcomes of treatment in terms of survival and health related quality of life.

2.2. Trial design and configuration

A three parallel groups randomised controlled trial.

2.3. Trial centres

Recruitment to the trial took place at general practices in and around nine study centres across the UK: Newcastle, Sheffield, Bristol, Cardiff, Edinburgh, Birmingham, Leicester, Cambridge, and Leeds.

2.4. Eligibility criteria

2.4.1. Inclusion criteria

- Men
- Age 50-69 years on the date of preparation at the general practice of the list of potential participants
- Able to give written informed consent to participate
- Fit for any of the three treatments and with a life expectancy of at least 10 years
- Registration with the participating general practice on the date of the PCC
- For randomisation: clinically localized prostate cancer (confirmed by isotope bone scan in men with PSA of 10ng/L or more) diagnosed by 10-core biopsy following a PSA level of 3ng/L or more.

2.4.2. Exclusion criteria

- Concomitant or past malignancies (other than a small treated skin cancer)
- Prior treatment for prostate malignancy
- Serious cardiac or respiratory problems in the previous 12 months of the PCC, e.g. stroke, MI, heart failure, COPD
- Kidney dialyses or transplantation
- Bilateral hip replacement
- Previous entry to the ProtecT study at a prior general practice
- PSA 20ng/L or more at diagnosis

2.5. Description of interventions

The **Active Monitoring Protocol** aimed to avoid immediate radical treatment whilst assessing the disease over time, with a review and the opportunity for radical treatment if there was evidence of disease progression. PSA levels were measured and reviewed every three months in the first year and twice yearly thereafter. Changes in PSA levels were assessed, and a rise of at least 50% over the previous 12 months triggered repeat testing within six to nine weeks. If the PSA levels were persistently raised, or th e patient had other concerns, a review appointment was made to consider treatment options.

The **Radiotherapy Protocol** began with neoadjuvant androgen suppression, given for three to six months before and concomitantly with 3D-conformal radiation therapy delivered at 74 Gy in 37 fractions.

Surgery was a radical retropubic prostatectomy procedure. The surgical approach was left to the discretion of the surgeon, and was most commonly open, but laparoscopic, or robot-assisted approaches were permitted from 2003.

2.6. Randomisation procedures

Randomisation was stratified by centre with stochastic minimization by age at invitation, Gleason score (primary and secondary grades), and mean of baseline and first biopsy PSA results. Men who declined randomisation were offered identical follow-up and formed an observational patient preference cohort.

2.7. Blinding

The process used to assess cause of death was adapted from the PLCO algorithm and ERSPC process. The medical records of deceased participants were summarised by trained researchers, anonymised and reviewed by an independent endpoint committee. Table 1 presents the classification of deaths by study arm.

2.8. Trial committees

For the current period of follow-up, ProtecT has a Trial Steering Committee, chaired by Professor Deborah Ashby (Imperial College).

2.9. Outcome measures

2.9.1. Primary outcome

The primary outcome is definite or probable prostate cancer mortality, including intervention-related deaths, at a median 10 years' follow-up.

We will repeat the analysis of definite or probable prostate cancer mortality, including intervention-related deaths, at 10 years (with all participating men having more than 10 years' follow-up) and at the median 15 years' follow-up.

As previously the plan is for the primary outcome measure to be determined by the independent cause of death committee. If this proves not possible, we will rely on certified underlying cause of death where necessary.

2.9.2. Secondary outcomes

Secondary clinical and patient-reported outcomes to be presented in the 15-year results papers are:

- *overall mortality*

- *metastatic disease*
- *clinical disease progression*
- *initiation of long-term hormone therapy*
- *patient reported outcomes (PROMs)*

Metastatic disease is defined as positive imaging showing bony, visceral and/or lymph node metastases, or PSA above 100; or bone marrow infiltration with associated systemic symptoms.

Clinical disease progression will be measured as person-years free of the consequences of disease progression. Signs of disease progression will include evidence of metastatic disease; the initiation of long-term hormone therapy; diagnosis of clinical T3 or T4 disease; or ureteric obstruction, rectal fistula, or the need for a permanent catheter when these are not considered to be a complication of treatment. There will be a review of cases where disease progression or metastatic disease are uncertain.

As the ascertainment of clinical disease progression may differ between the three study arms, we will also present the initiation of long-term hormone therapy, to indicate those men whose disease is no longer curable.

The reporting of metastatic disease, clinical disease progression, and initiation of long-term hormone therapy is conditional on securing the data.

The patient-reported outcomes which have been measured are listed in the Appendix. These measures are derived from validated questionnaires and have been completed at recruitment, at first biopsy, six months after randomisation, and yearly thereafter. These measures will be reported in a separate companion paper, to be submitted for publication at the same time as the primary outcomes paper.

2.10. Interim analysis

There have been no analyses of the outcome data that have accumulated since publication of the findings at median ten-years follow-up.

3. GENERAL ANALYSIS CONSIDERATIONS

3.1. Analysis populations

The primary analysis data set is all men **randomly allocated** to one of the three management options being compared in the ProtecT trial.

3.2. Procedures for missing data

Where a man has omitted responding to a small number of items on a patient reported outcome measure, these will be imputed as per the guidance for that measure.

Where the patient has not responded to any or most of the items on a measure, the main analysis of patient-reported outcomes will NOT be based on data with those missing scale scores imputed. However, the amount of missing data, by allocation arm, will be presented. All men providing at least one post-randomisation patient-reported measure will be included in the relevant analysis.

3.3 Definitions of treatment received

Men were considered to have received each of the treatments according to the following definitions; men who did not fulfil these were excluded.

1. Active Monitoring (AM) if there were ≥ two PSA tests and no radical treatment in the 12 months following diagnosis.
2. Surgery (RP) if RP carried out within 12 months following diagnosis.
3. Radiotherapy (RT) if treatment protocol was started within 12 and completed within 15 months.

4. DESCRIPTION OF PARTICIPANT CHARACTERISTICS

4.1. Disposition

Details of the recruitment of the ProtecT randomised trial cohort, up to the point of randomisation, were presented in the *Baseline Paper* (Lane et al, 2014). Details of how many men were excluded and for what reasons are presented. The subsequent flow of patients through the trial will be summarised in a CONSORT diagram that will include the numbers randomised to the three treatment groups, losses to follow-up and the numbers analysed. *This extends the diagram in Hamdy et al (2016) to make clear losses to follow-up since the median ten-year follow-up (Figure 1).*

5. ANALYSIS OF EFFECTIVENESS

5.1. Summary of outcomes to report at median 15 years follow-up

The following summaries of the outcome events will be presented for each treatment allocation group:

- Number of deaths due to prostate cancer.
- Prostate cancer mortality at 15 years, with 95% confidence interval.
- Prostate cancer mortality per 1,000 person years of follow-up, with 95% confidence interval.
- Kaplan-Meier survival of death from prostate cancer as a function over time.

The following summaries will be presented for the clinical secondary outcome events:

- Number of events.
- Events per 1000 person years of follow-up, with 95% confidence interval.
- Kaplan-Meier overall survival and survival free of disease progression as functions over time

In addition, if the data can be obtained, the Kaplan-Meier cumulative incidence of the uptake of radical treatment will be presented for the three treatment groups as a function over time.

5.2. Prostate cancer mortality at median 15 years follow-up

This analysis will be conducted on an intention-to-treat basis comparing allocated groups. Deaths occurring until a median of 15-year follow-up has accumulated (23:59 on Monday 23rd November 2020) will be included in the locked database. We will allow up to 30th June 2021 to be notified of deaths (we may revise this deadline for notification if the COVID-19 outbreak causes delays).

Prostate cancer (definite, probable, or intervention-related) mortality will be compared between the three treatment groups using Cox's proportional hazards regression adjusted for study centre (all nine centres distinguished using dummy variables), age at baseline (continuous measure in years), Gleason score (2-6, 7, 8-10), and PSA at baseline (continuous measure in ng/ml, log-transformed to accommodate positive skewed distribution):

$$\boldsymbol{h}(t) = \boldsymbol{h}_0(t)\exp\{\beta_{1j}x_{1i} + \beta_{2k}x_{2i} + \beta_3 x_{3i} + \beta_{4m}x_{4i} + \beta_5 ln.x_{5i}\}$$

β_{1j} is the log hazard ratio comparing two of the treatment arms, with two of the three pairwise comparisons being available from a single iteration of the analysis (i.e. j=1,2 the estimated comparisons depending on the choice of comparator treatment). x_{1i} is the treatment allocation (0,1,2) for participant i. β_{2k} (k=1 to 8) captures differences in the hazard of the primary outcome event between study centres, x_{2i} being the study centre for participant i. β_3 is the linear effect of age, with x_{3i} being the age in years for participant i. β_{4m} (m=1,2) accommodates the effect of Gleason score category with x_{4i} being the Gleason score category for participant i. Finally β_5 is the linear effect of log-transformed PSA level, x_{5i} being the PSA level at diagnosis for participant i.

Plots and tests based upon Schoenfeld residuals will be investigated to determine whether the proportional hazards assumption is reasonable; if not the model will be elaborated to allow for a changing magnitude of treatment effect with time.

The hazard ratio and 95% confidence interval for the treatment effect estimated in each pairwise comparison of allocated treatments will be presented, but pairwise significance tests will only be conducted if a test of an equal 15-year disease specific mortality risk across all three arms yields a p-value of less than 0.05 (Table 3). This conditional approach keeps the overall false positive rate at 5% and has been found to maintain power in simulation studies (Bauer 1991).

The competing risk of all-cause death is not anticipated to influence the estimation of the treatment effect on the risk of prostate cancer mortality. Age is the only strongly influential risk factor shared by all-cause and prostate cancer mortality, and age is included as a covariate in all models.

5.3. Other analyses at median 15-years follow-up

The approach to the primary analysis will be adapted to the analysis of secondary outcome events, i.e. definite, probable or possible prostate cancer mortality; all-cause mortality; and metastatic cancer.

For patient reported outcomes (see Section 2.9.2) summary statistics by allocated group will be presented graphically for the baseline, 6-month, 12-month and subsequent assessment points up to 12 years (we will accept questionnaires returned by September 30th 2021, by which time all men will have this duration of follow-up). This approach will also be taken for those questionnaires dropped from the battery in November 2018, with the consequent loss of responses for later assessment points being made clear. All graphical presentations will present the full 12 years follow-up but will focus on the novel data for the period from 73 to 132 months.

Analyses will employ multi-level models for repeated measures to estimate average treatment effects. These analyses will be adjusted for the stratification (centre) and minimisation (baseline age, Gleason score, PSA level) variables as described in the previous section. Consistent with the focus of the current analysis, the null

hypothesis of no difference in the population means of an outcome measure between allocated treatment groups, for the period from 73 to 144 months, will be tested.

5.4. Subgroup analyses

Following peer-reviewed criteria for the credibility of subgroup analyses (Sun et al, 2010), we have pre-specified a small number of subgroup analyses investigating whether treatment effectiveness in reducing prostate cancer specific mortality is modified by the following factors measured at diagnosis:

- Age (above versus below 65 years)
- Grade Group (Group 1 versus Group 2; Group 2 versus Group 3+)
- PSA (PSA < 10ng/ml versus 10 and above)
- Clinical stage (T1 versus T2)
- Aggregate tumour length in biopsy cores (<4mm versus 4mm+)
- Maximum tumour length in a single biopsy core (<2mm versus 2mm+)
- D'Amico low risk versus moderate / high risk.
- CAPRA low risk (score 0-1) versus moderate / high risk (>1)

Age, clinical stage, grade group, tumour burden and PSA are commonly used factors in the prediction of risk of disease progression. We anticipate that men at the lowest risk of disease progression have least to gain from radical treatment in comparison to their outcome with active monitoring. Wilt et al (2020) obtained results consistent with this hypothesis.

The D'Amico risk categories (D'Amico, 1998) are **low** (Gleason score is 6 or less, and PSA is 10ng/ml or less, and clinical stage is T1c/T2a); **high** (Gleason score is 8 or more, or PSA more than 20ng/ml, or stage is T2c); and **intermediate** (Gleason score is 7, or PSA is higher than 10ng/ml but no more than 20ng/ml, or stage is T2b).

To facilitate comparison of our results to those of Wilt et al (2020) we have adopted the CAPRA score of disease risk (Cooperberg et al, 2006) and will repeat this set of subgroup analyses with all-cause mortality as the outcome measure.

The statistical models used in the primary analysis will be extended to incorporate interaction terms, to test null hypotheses of no variation in treatment effect across subgroups. For sub-group analyses based on age, PSA, and tumour length measures, the interaction test will be based on the continuous measure, and departures from the assumption of a linear relationship will be investigated (and accommodated if necessary) by introducing polynomial terms. Significance testing will be conducted with the principles of the primary analysis being followed as closely as possible.

We will also investigate whether the relative impact of intervention on key PROMs (pad use, nocturia, erectile dysfunction , and bloody stools) is modified by age and the risk of disease progression at baseline, as incorporated for the clinical measures.

5.5. Sensitivity analyses

The analysis of prostate cancer mortality will be repeated, but with the outcome defined as death definitely, probably **and possibly** due to prostate cancer.

6. 15-YEAR PUBLICATION PLAN

6.1. Planned papers and timelines

The intention is to present the clinical and patient-reported outcomes in a pair of papers, to be submitted to a high impact medical journal by late 2021. With a census date of **23rd November 2020** for clinical outcomes contributing to this analysis, this will allow those routine data available by 30th June 2021 to be incorporated.

7. FINAL REPORT TABLES AND FIGURES

Figure 1. CONSORT flowchart, illustrating the flow of participants through each of the three arms of the trial, from the point of randomisation.

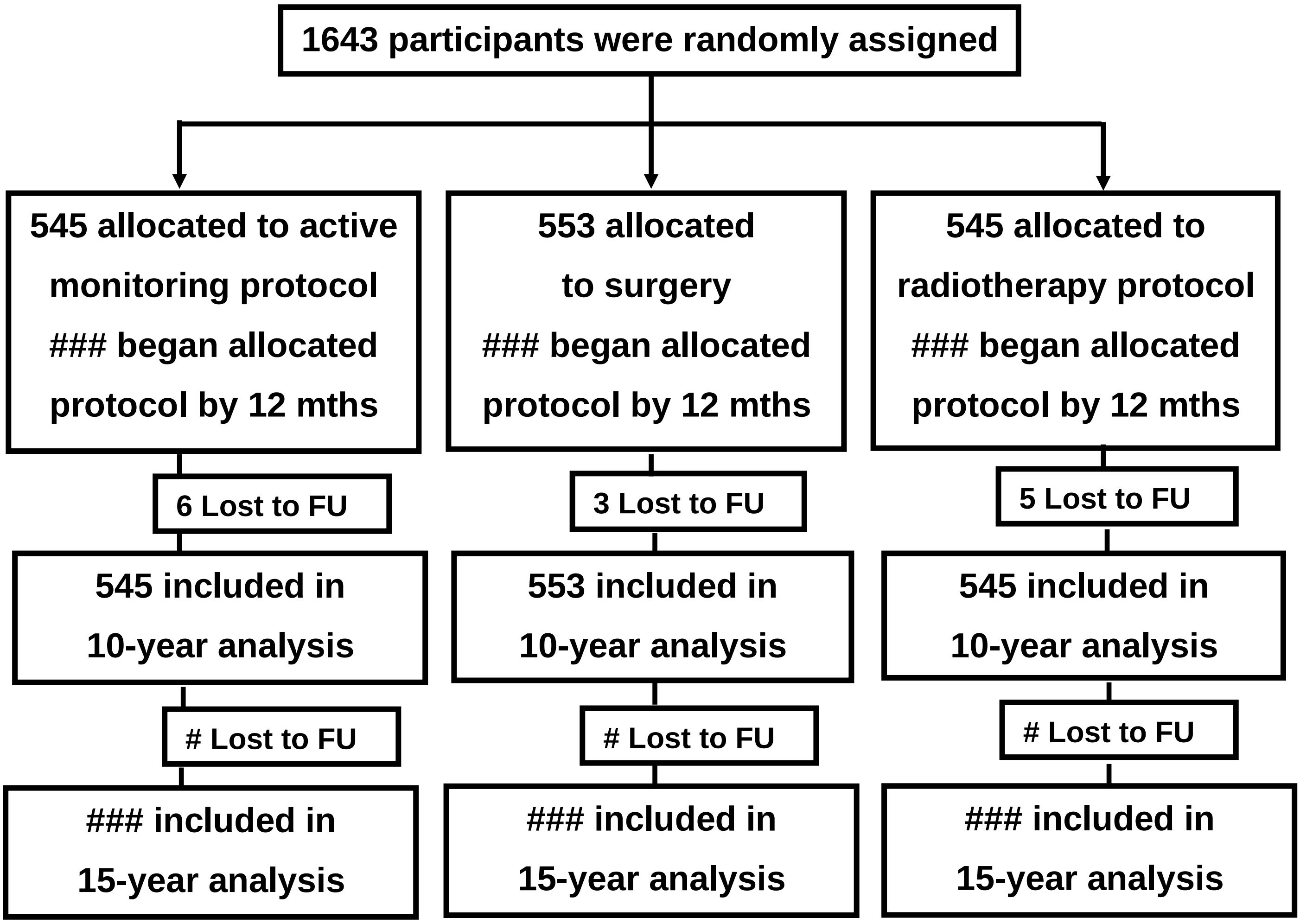

Figure 2. Kaplan-Meier estimates of the cumulative probability of undergoing radical interventions during the follow-up period, according to treatment group.

Radical intervention was defined as a radical prostatectomy, per-protocol radiotherapy, non-protocol radiotherapy (including brachytherapy), or high-intensity focussed ultrasound therapy.

Figure 3. Kaplan-Meier estimates of prostate cancer-specific survival and freedom from disease progression, according to treatment group: active monitoring (solid line), surgery (long dash line) and radiotherapy (short dash line) treatment groups

Panel A shows the rate of prostate cancer-specific survival. Prostate cancer-specific deaths were those that were definitely or probably due to prostate cancer as determined by an independent cause-of-death evaluation committee whose members were unaware of treatment assignments.

Panel B shows the rate of overall survival.

Panel C shows the rate of freedom from disease progression. Clinical progression of prostate cancer included metastases and death due to prostate cancer or its treatment.

Table 1. Prostate cancer mortality, Clinical progression, metastatic disease and all-cause mortality, by randomised group

	Active monitoring protocol (N=545)	Surgery (N=553)	Radiotherapy protocol (N=545)	p-value[1]
Total person years in follow-up				
Number of deaths due to prostate cancer[2]				
% prostate cancer mortality at median 10 years (95% CI)				
% prostate cancer mortality at median 15 years (95% CI)				
Prostate cancer deaths[1] per 1000 person years (95% CI)				
Number of deaths due to any cause				
All-cause deaths per 1000 person years (95% CI)				
Person years of follow-up free of hormone treatment				
Number of men treated with hormones for advanced disease				
Starting hormone treatment per 1000 person years (95% CI)				
Person years of follow-up free of clinical progression[3]				
Number of men with clinical progression				
Clinical progression per 1000 person years (95% CI)				
Person years of follow-up free of metastatic disease				
Number of men with metastatic disease				
Metastatic disease per 1000 person years (95% CI)				

[1] Likelihood ratio test of the null hypothesis “no difference in prostate cancer mortality between the three treatment arms”, adjusted for study centre, age, mean PSA at prostate check clinic and biopsy, and Gleason score at baseline. [2]Death probably or definitely due to prostate cancer or its treatment as judged by an independent committee. [3]Disease progression includes evidence of metastatic disease; the initiation of hormone therapy; diagnosis of clinical T3 or T4 disease; or ureteric obstruction, rectal fistula, or the need for a permanent catheter when these are not considered to be a complication of treatment.

Table 2. Prostate cancer deaths by randomised group and subgroup

	Rate prostate cancer mortality[1] per 1000 person years (number of deaths)			
	Active monitoring protocol (N=545)	Surgery (N=553)	Radiotherapy protocol (N=545)	p-value[2]
Age at randomization				
< 65 years				
65 years+				
Grade group at diagnosis				
Group 1				
Group 2				
Group 3+				
Aggregate tumour length in biopsy cores				
<4mm				
4mm+				
Maximum tumour length in any single biopsy core				
<2mm				
2mm+				
PSA level at diagnosis				
< 10 ng/ml				
10 ng/ml+				
Clinical stage at diagnosis				
T1c				
T2				
CAPRA risk score				
Low risk (score 0-1)				
Medium / high risk (score >1)				
D'Amico risk group				
Low				
Intermediate / high				

[1]Death probably or definitely due to prostate cancer or its treatment as judged by an independent committee.[2]Likelihood ratio test of the null hypothesis "equal relative treatment effects across the subgroups", adjusted for study centre, age, mean PSA at prostate check clinic and biopsy, and Gleason score at baseline.

8. APPENDIX

The following standard assessment tools have been completed by men participating in the ProtecT study:

- Expanded Prostate Index Composite
- International Consultation on Incontinence Questionnaire (ICIQ)
- International Continence Society urinary function (ICS*male*SF)
- EORTC QLQ-C30 cancer-specific impacts
- Hospital Anxiety and Depression Scale (Until November 2018)
- Short Form 12 (SF-12) mental and physical subscales (Until November 2018)
- EuroQoL-5D (EQ-5D) generic quality of life

9. REFERENCES

Bauer P. (1991). Multiple testing in clinical trials. **Statistics in Medicine 10:**871-890.

Bokhorst LP, Bangma CH, van Leenders GJLH, et al. (2014). Prostate-specific antigen-based prostate cancer screening: reduction of prostate cancer mortality after correction for nonattendance and contamination in the Rotterdam section of the European Randomised Study of Screening for Prostate Cancer. **European Urology 65:**329-336.

Cooperberg MR, Freedland SJ, Elkin EP, Presti JC, Amling CL, Terris MK, Aronson WJ, Kane CJ, Carroll PR. (2006). Multiinstitutional validation of the UCSF Cancer of the Prostate Risk Assessment for prediction of recurrence after radical prostatectomy. Cancer 107:2384-91.

Cuzick J, Sasieni P, Myles J, Tyrer J. (2007). Estimating the effect of treatment in a proportional hazards model in the presence on non-compliance and contamination. **Journal of the Royal Statistical Society, Series B 69:**565-588

D'Amico AV, Whittington R, Malkowicz SB, Schultz D, Blank K, Broderick GA et al. (1998). Biochemical outcome after radical prostatectomy, external beam radiation therapy, or interstitial radiation therapy for clinically localized prostate cancer. **JAMA 280**: 969-74.

Hamdy FC, Donovan JL, Lane JA, Mason M, Metcalfe C, Holding P, Davis M, Peters TJ, Turner EL, Martin RM, Oxley J, Robinson M, Staffurth J, Walsh E, Bollina P, Catto J, Doble A, Doherty A, Gillatt D, Kockelbergh R, Kynaston H, Paul A, Powell P, Prescott S, Rosario DJ, Rowe E, Neal DE. (2016). 10-year outcomes after monitoring, surgery, or radiotherapy for localized prostate cancer. **NEJM 375**:1415-24.

Hampson L, Metcalfe C. (2012). Incorporating prognostic factors into causal estimators: A comparison of methods for randomised controlled trials with a time-to-event outcome. **Statistics in Medicine 31:** 3073-3088.

Lane JA, Donovan JL, Davis M, et al for the ProtecT study group. (2014). The ProtecT (Prostate testing for cancer and Treatment) trial: study design, diagnostic process and baseline results. **Lancet Oncology 15:**1109-1118.

Loeys T, Goetghebeur E. (2003). A causal proportional hazards estimator for the effect of treatment actually received in a randomized trial with all-or-nothing compliance. **Biometrics 59:**100-105

Metcalfe C. (2014). Can the results of the European Randomized Study of Screening for Prostate Cancer be decontaminated? **European Urology 65:**337-338.

Roach M, Hanks G, Thames H, Schellhammer P, Shipley WU, Sokol GH, Sandler H. (2006). Defining biochemical failure following radiotherapy with or without hormonal

therapy in men with clinically localized prostate cancer: recommendations of the RTOG-ASTRO Phoenix consensus conference. **International Journal of Radiation Oncology, Biology & Physics 65:**965-974.

Sun X, Briel M, Walter SD, Guyatt GH. (2010). Is a subgroup effect believable? Updating criteria to evaluate the credibility of subgroup analyses. **BMJ** 340:c117.

Wilt TJ, Vo TN, Langsetmo L, Dahm P, Wheeler T, Aronson WJ, Cooperberg MR, Taylor BC, Brawer MK. (2020). Radical prostatectomy or observation for clinically localised prostate cancer: extended follow-up of the prostate cancer intervention versus observation trial (PIVOT). **European Urology** 77: 713-724.

Summary of changes to the ProtecT Trial Statistical Analysis Plan (SAP) 2001-2020

**Summary of SAPs**
Two separate SAPs were published in 2015 and 2020 before access to the data. Each was developed specifically for the median 10-year and median 15-year analyses respectively. The majority of aspects, including the primary outcome (prostate cancer specific mortality), were unchanged. The following additions were made to the median 15-year follow-up SAP:<br>Secondary clinical and patient-reported outcomes: added initiation of long-term hormone therapy<br>Pre-specified subgroups were added/updated as follows:<br>• Gleason score updated to Gleason Grade Group (Group 1 versus Group 2; Group 2 versus Group 3+).<br>• Added aggregate tumour length in biopsy cores (<4mm versus 4mm+).<br>• Added maximum tumour length in a single biopsy core (<2mm versus 2mm+).<br>• Added D'Amico baseline risk stratification system: low risk versus moderate / high risk.<br>• Added CAPRA baseline risk stratification system: low risk versus moderate / high risk.<br>• Added age and risk stratification at baseline for key PROMs (pad use, nocturia, erectile dysfunction, and bloody stools).
