# Supplementary material for: Long‐term outcomes of cribriform‐positive and cribriform‐negative prostate cancer treated with radical prostatectomy in the ProtecT trial
Source: BJU Int. 2026 Mar 27;137(6):984–91. doi: 10.1111/bju.70261 (PMC13168917; doi:10.1111/bju.70261)
Supplement: Supplementary file 2 — Table S1. Site‐specific distribution of RP specimens that were available for centralised histological review as part of this study. The notably higher representation of specimens from the Cambridge site is attributed to the fact that the centralised review was led by the Cambridge‐based research team. This provided the investigators with direct access to local pathology archives for retrospective retrieval of whole‐mount slides that had not been made available as part of the original centralisation exercise. It should be noted that no historical pathological data on cribriform morphology (ICC or IDC) was available for the unreviewed specimens, as these features were not part of the original trial's pathology proforma conducted according to 2005 ISUP recommendations. Table S2. Distribution of randomisation characteristics between the ProtecT participants included in this analysis and those whose data were missing from this analysis. Note that the biopsy Gleason score provided in this table was derived from the original proforma using the 2005 ISUP guidelines. It should also be noted that no historical pathological data on cribriform morphology (ICC or IDC) was available for the unreviewed specimens, as these features were not part of the original trial pathology proforma completed according to 2005 ISUP recommendations. Table S3. Sensitivity and specificity of systematic biopsies for assessing binary cribriform status compared to ground‐truth RP histopathological assessment. Table S4. Sensitivity and specificity of systematic biopsies for detecting patients with cribriform‐positive, GG ≥3 and/or pT3b disease compared to ground‐truth RP histopathological assessment. Table S5. Prevalence of cribriform‐positive patients in the treatment‐received cohort with ICC, IDC, or both ICC and IDC in their RP specimens. Please note that given the lack of basal cell immunohistochemistry (IHC) in all cases, the distinction between ICC and IDC was made based on morphological haematox [file BJU-137-984-s002.docx]

**Long-term outcomes of cribriform-positive and cribriform-negative prostate cancer treated with surgery in the ProtecT trial**

**Supplement 1**

| **Trial recruitment site** | **Proportion of reviewed surgical specimens (%)** |
| --- | --- |
| Cambridge | 34% |
| Newcastle | 14% |
| Sheffield | 13% |
| Cardiff | 12% |
| Bristol | 10% |
| Edinburgh | 9% |
| Leicester | 6% |
| Leeds | 1% |
| Birmingham | 1% |

**Supplementary Table 1**. **Site-specific distribution of surgical specimens which were available for centralised histological review as part of this study.** The notably higher representation of specimens from the Cambridge site is attributed to the fact that the centralised review was led by the Cambridge-based research team. This provided the investigators with direct access to local pathology archives for retrospective retrieval of whole-mount slides which had not been made available as part of the original centralisation exercise. It should be noted that no historical pathological data on cribriform morphology (ICC or IDC) was available for the unreviewed specimens, as these features were not part of the original trial's pathology proforma conducted according to 2005 ISUP recommendations.

| **Randomisation variable** | **Patients included in this study (n = 480)** | **Patients excluded from this study due to lack of follow-up data or unavailable surgical specimens (n = 281)** |
| --- | --- | --- |
| Age, years  <65  ≥65 | 61%  39% | 59%  41% |
| PSA, ng/mL  <10  ≥10 | 90%  10% | 89%  11% |
| Biopsy Gleason score  6  7  8 | 58%  41%  1% | 59%  40%  1% |

**Supplementary Table 2. Distribution of randomisation characteristics between the ProtecT participants included in this analysis and those whose data were missing from this analysis.** Note that the biopsy Gleason score provided in this table was derived from the original proforma using the 2005 ISUP guidelines. It should also be noted that no historical pathological data on cribriform morphology (ICC or IDC) was available for the unreviewed specimens, as these features were not part of the original trial pathology proforma completed according to 2005 ISUP recommendations.

| **Parameter** | **Value** |
| --- | --- |
| True positive, n | 17 |
| False positive, n | 3 |
| True negative, n | 77 |
| False negative, n | 26 |
| Sensitivity, % (95% CI) | 39.5% (25.0%–55.6%) |
| Specificity, % (95% CI) | 96.3% (89.4%–99.2%) |

**Supplementary Table 3.** **Sensitivity and specificity of systematic biopsies for assessing binary cribriform status compared to ground-truth radical prostatectomy histopathological assessment.**

| **Parameter** | **Value** |
| --- | --- |
| True positive, n | 9 |
| False positive, n | 7 |
| True negative, n | 95 |
| False negative, n | 8 |
| Sensitivity, % (95% CI) | 52.9% (27.8%–77.0%) |
| Specificity, % (95% CI) | 93.1% (86.4%–97.2%) |

**Supplementary Table 4.** **Sensitivity and specificity of systematic biopsies for detecting patients with cribriform-positive, ≥GG3 and/or pT3b disease compared to ground-truth radical prostatectomy histopathological assessment.**

| **Characteristic** | **Number of patients (%)** |
| --- | --- |
| **Treatment-received cohort: all cribriform-positive patients (n = 143 patients)** | |
| ICC only | 77 (53.8%) |
| IDC only | 24 (16.8%) |
| Both ICC and IDC present in the specimen | 42 (29.4%) |
| **Treatment-received cohort: cribriform-positive patients with metastatic or lethal disease (n = 21 patients)** | |
| ICC only | 8 (38.1%) |
| IDC only | 2 (9.5%) |
| Both ICC and IDC present in the specimen | 11 (52.4%) |

**Supplementary Table 5. Prevalence of cribriform-positive patients in the treatment-received cohort with invasive cribriform carcinoma (ICC), intraductal carcinoma (IDC), or both ICC and IDC in their surgical specimens.** Please note that given the lack of basal cell immunohistochemistry (IHC) in all cases, the distinction between ICC and IDC was made based on morphological H&E assessment according to the ISUP consensus document. Given the limitations of this approach, these descriptive and hypothesis-generating results should be interpreted with caution.

**
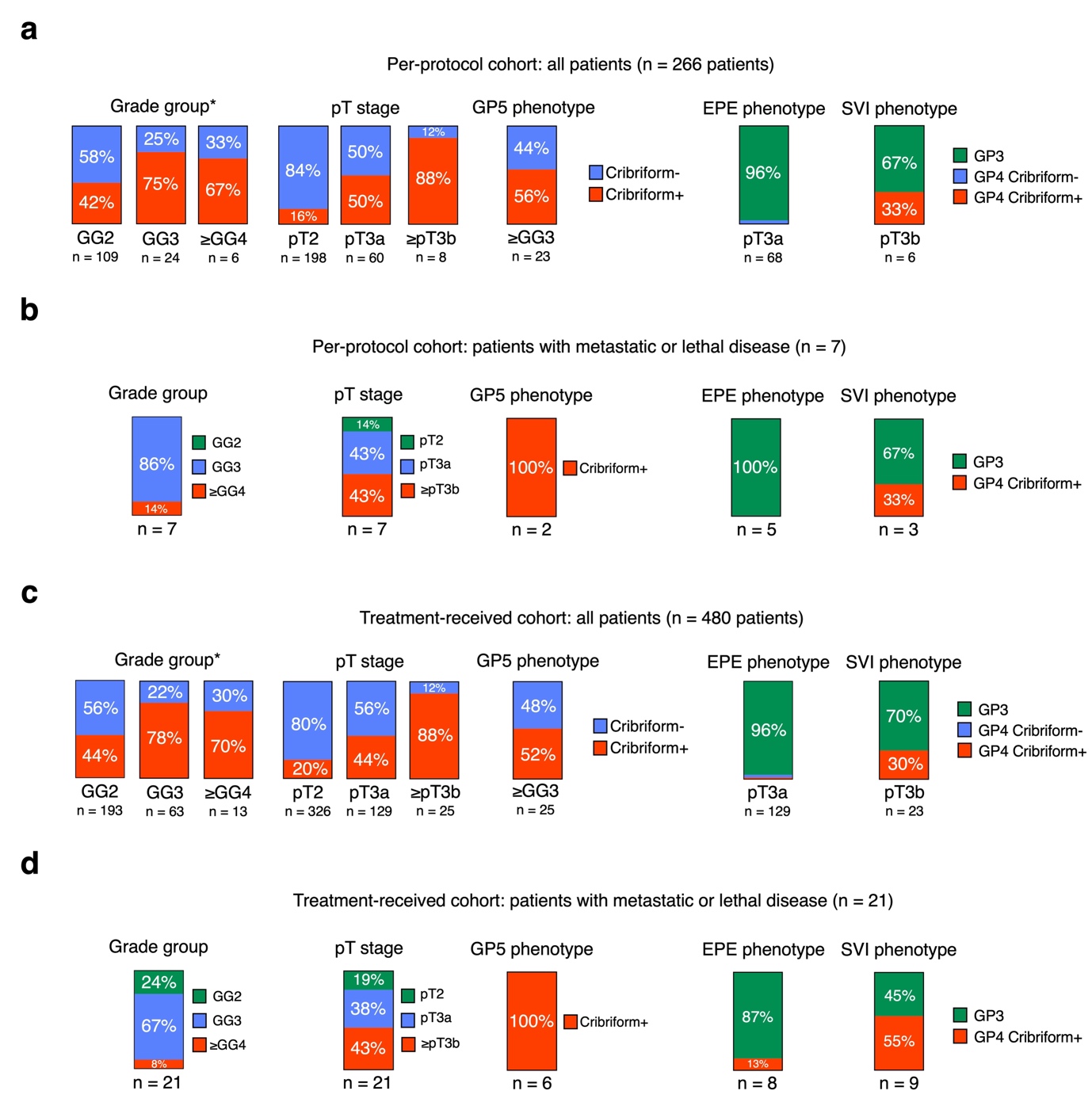
**

**Supplementary Figure 1. Detailed Histopathological Characteristics. (A-B)** Data are from the per-protocol cohort. **(C-D)** Data are from the treatment-received cohort. EPE indicates extraprostatic extension; GG, grade group; GP3-5, Gleason pattern 3-5; SVI, seminal vesicle invasion. * Note that all patients with GG1 disease are cribriform-negative by definition.
